# Structural Basis of 2-Phenylamino-4-phenoxyquinoline Derivatives as Potent HIV-1 Non-Nucleoside Reverse Transcriptase Inhibitors

**DOI:** 10.3390/molecules27020461

**Published:** 2022-01-11

**Authors:** Arthit Makarasen, Suwicha Patnin, Pongsit Vijitphan, Nanthawan Reukngam, Panita Khlaychan, Mayuso Kuno, Pakamas Intachote, Busakorn Saimanee, Suchada Sengsai, Supanna Techasakul

**Affiliations:** 1 Laboratory of Organic Synthesis, Department of Chemistry, Chulabhorn Research Institute, Bangkok 10210, Thailand; arthit@cri.or.th (A.M.); suwicha@cri.or.th (S.P.); pongsit@cri.or.th (P.V.); nanthawan@cri.or.th (N.R.); panita@cri.or.th (P.K.); 2 Department of Chemistry, Faculty of Science, Srinakharinwirot University, Bangkok 10110, Thailand; mayuso@g.swu.ac.th; 3 Biological Activity Test and Screening Unit, Central Facilities, Chulabhorn Research Institute, Bangkok 10210, Thailand; pakamas@cri.or.th (P.I.); busakorn@cri.or.th (B.S.); suchada@cri.or.th (S.S.)

**Keywords:** quinoline, molecular docking, HIV-1, cytotoxic, anti-cancer

## Abstract

New target molecules, namely, 2-phenylamino-4-phenoxyquinoline derivatives, were designed using a molecular hybridization approach, which was accomplished by fusing the pharmacophore structures of three currently available drugs: nevirapine, efavirenz, and rilpivirine. The discovery of disubstituted quinoline indicated that the pyridinylamino substituent at the 2-position of quinoline plays an important role in its inhibitory activity against HIV-1 RT. The highly potent HIV-1 RT inhibitors, namely, 4-(2′,6′-dimethyl-4′-formylphenoxy)-2-(5″-cyanopyridin-2″ylamino)quinoline (**6b**) and 4-(2′,6′-dimethyl-4′-cyanophenoxy)-2-(5″-cyanopyridin-2″ylamino)quinoline (**6d**) exhibited half-maximal inhibitory concentrations (IC50) of 1.93 and 1.22 µM, respectively, which are similar to that of nevirapine (IC50 = 1.05 µM). The molecular docking results for these two compounds showed that both compounds interacted with Lys101, His235, and Pro236 residues through hydrogen bonding and interacted with Tyr188, Trp229, and Tyr318 residues through π–π stacking in HIV-1 RT. Interestingly, **6b** was highly cytotoxic against MOLT-3 (acute lymphoblastic leukemia), HeLA (cervical carcinoma), and HL-60 (promyeloblast) cells with IC50 values of 12.7 ± 1.1, 25.7 ± 0.8, and 20.5 ± 2.1 µM, respectively. However, **6b** and **6d** had very low and no cytotoxicity, respectively, to-ward normal embryonic lung (MRC-5) cells. Therefore, the synthesis and biological evaluation of 2-phenylamino-4-phenoxyquinoline derivatives can serve as an excellent basis for the development of highly effective anti-HIV-1 and anticancer agents in the near future.

## 1. Introduction

Quinoline derivatives are an important class of heterocycles that exist among the principal components of natural products [1,2]. Quinoline is widely used as a dominant compound to synthesize molecules with medical benefits. Quinolines are used as anti-cancer, antimycobacterial, antimicrobial, anticonvulsant, anti-inflammatory, and cardiovascular agents [3,4,5]. HIV-1 reverse transcriptase (RT) is an important enzyme involved in retroviral replication and represents an important target for the development of anti-HIV drugs. Highly active antiretroviral therapy (HAART) has provided substantial progress in the treatment of acquired immunodeficiency syndrome (AIDS). HAART relies on three inhibitors, such as reverse transcriptase (RT) and protease enzymes, to sufficiently control HIV infection [6,7,8]. Non-nucleoside reverse transcriptase inhibitors (NNRTIs) play an important role in HAART because of their unique antiviral activity, high specificity, and low cytotoxicity. Commercially available first-generation NNRTIs, namely, nevirapine (NVP), delavirdine, and efavirenz (EFV), can directly interfere with HIV-1 RT by binding to an allosteric site in similar positions [9,10]; however, the rapid emergence of mutant viral strains has severely compromised the clinical use of these HIV-1 RT inhibitors [11]. Diarylpyrimidine derivatives, such as etravirine (ETR, TMC125) and rilpivirine (RPV, TMC278), are second-generation NNRTIs that have been approved by the FDA for clinical use. Both compounds are highly active against a large panel of HIV-1 mutant strains and exhibit excellent drug resistance profiles [12,13]. Searching for novel agents with higher potency and/or lower toxicity is necessary because of the emergence of drug resistance caused by strain mutation during the long-term use of NNRTIs. Phenylamino-phenoxyquinoline derivatives have been developed using the molecular hybridization approach from currently available drugs, namely, NVP, EFV, and RPV, to generate novel series of quinoline derivatives and explore new anti-HIV agents. Quinoline was designed and used as the core structure resulting from the molecular docking and overlaying between NVP, EFV, and RPV. In addition, EFV and RPV have their substituents with V-shape-like branches; the substituent positions in the quinoline were designed for 4,6 or 2,4-disubstitution [14]. On the discovery of phenylamino-phenoxyquinoline, a previous report concluded that 2-phenylamino-4-phenoxy-quinoline derivatives may have the potential to inhibit HIV-1 RT in a stronger manner than 6-phenylamino-4-phenoxyquinoline derivatives, because 6-phenylamino-4-phenoxy-quinoline derivatives have different conformations and interactions at an allosteric site of HIV-1 RT. Previous studies showed that the intermolecular interaction between 2-phenylamino-4-phenoxyquinoline (**5c**) and transport proteins, namely, bovine serum albumin (BSA) and human serum albumin (HSA), is spontaneous, and the main interaction forces are Van der Waals forces and hydrogen bonding. **5c** bound into the cavity of the transport protein using hydrophobic interaction and hydrogen bonding interaction via the aromatic rings and cyanide groups in the side chain of quinoline [15]. The interactions between serum albumin and drugs play an important physiological role in the transportation, distribution and metabolism of drugs in vivo. Herein, we develop and report the discovery of 2,4-disubstituent-quinolines able to inhibit the properties and cytotoxicity of HIV-1 RT. 2,4-Diphenyloxyquinoline derivatives (**4a**–**4d**), 2-pyridinylamino-4-phenyloxyquinoline derivatives (**6a**–**6d**), 2-phenyloxyquinoline-4-pyridinylamino derivatives (**8a**–**8d**), and other compounds (**7a**, **7b**, **9b**, **10b,** and **11b**) were developed and revealed in this report. Moreover, the cytotoxicity of compounds **5a**–**5d** against breast cancer, liver cancer, multidrug-resistant lung cancer, and promyeloblast cells was recalled to compare with other synthesized compounds. This study found that the substituent positions at 2-phenylamino, 4-phenoxy in the quinoline core structure, and the pyridinylamino group for the 2-phenylamino side chain have an important function in the inhibition of HIV-1 RT. The substituents in the core structure of quinoline gave insights into the important interactions between the NNRTI-binding pocket and the synthesized compounds, which could provide information for structural improvement based on the molecular design of potent NNRTIs from the molecular docking results. 

## 2. Results and Discussion

**Scheme 1:**
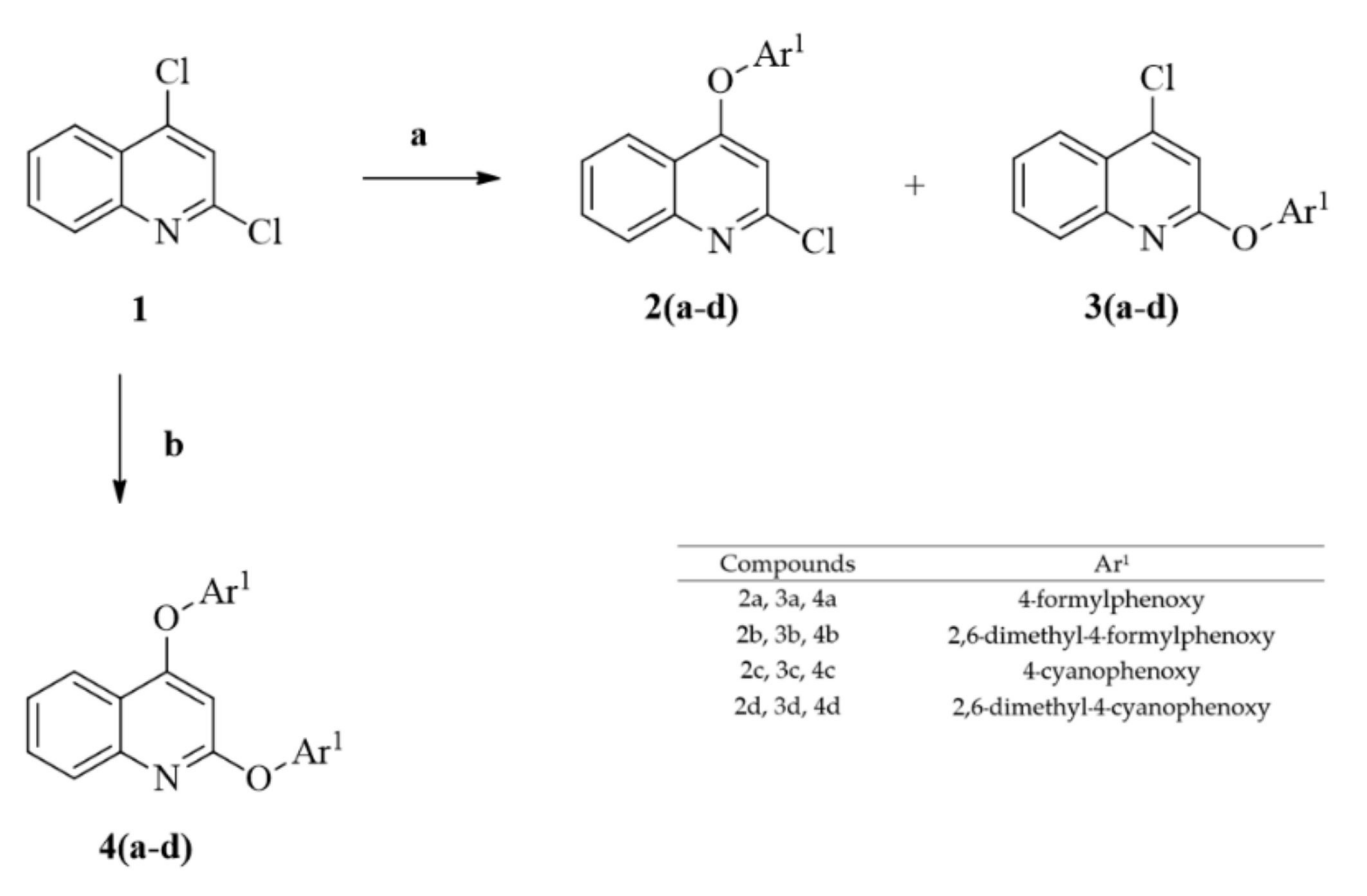
Synthesis of 2-chloro-4-phenoxyquinoline (**2a**–**2d**), 4-chloro-2-phenoxyquinoline (**3a**–**3d**), and 2,4-diphenoxyquinoline (**4a**–**4d**). Reagents and conditions: (**a**) hydroxyl benzene (Ar1–OH, 1.1 eq.), Cs_2_CO_3_ (2.0 eq), DMF, 80 °C sealed tube; (**b**) hydroxyl benzene (Ar1–OH, 2.1 eq.), Cs_2_CO_3_ (2.0 eq.), DMF, 120 °C sealed tube.

2-Phenylamino-4-phenoxyquinoline derivatives were evaluated via the molecular hybridization approach using the chromophores of HIV-1 RT inhibitors, namely, NVP, EFV, and RPV. 2,6-Dimethyl-4-formylphenoxy, 2,6-dimethyl-4-cyanophenoxy, and 4-cynophenyl amino were used as substituents in the core structure of quinoline to compare their inhibitory activity against HIV-RT related to the substituents of RPV and ETR in order to prevent unpredictable activity and confirm the potential of the designed compounds. Molecular docking was used to predict the binding site, binding energy, and the interaction between the designed compounds and HIV-1 RT. The literature revealed that 2-phenylamino-4-phenoxy-quinolines (**5a**–**5d**) bind to HIV-1 RT at an identical location and with low binding energy [14]. The binding interactions between **5a**–**5d** and the amino acid residue (Lys 101) in the active site of HIV-1 RT exhibited hydrogen bonding, similar to that for RPV. According to a previous report, docking analysis of 2,4-disubstituent-quinoline led to a structure-based design approach to the synthesis of the novel inhibitors of HIV-1 RT. In this paper, the discovery of 2-phenylamino-4-phenoxy-quinoline and other 2,4-disubstituted-quinoline derivatives combined with molecular modeling provides key information on the substituent in the core structure of the quinoline compound which corresponds in the binding pocket with amino acid residues of HIV-1 RT. The 2-chloro-4-phenoxyquinolines (**2a**–**2d**), were prepared from commercially available 2,4-dichloroquinoline using nucleophilic substitution under basic conditions. The target compounds, 2-phenylamino-4-phenoxy-quinolines (**5a**–**5d**), were produced via cross-coupling reactions between 2-chloro-4-phenoxyquinolines (**2a**–**2d**) and 4-aminobenzonitrile. According to the previous results, the production efficiency during the synthesis of **2a**–**2d** was moderated. The deeper investigation in the present study showed that 4-chloro-2-phenoxyquinolines (**3a**–**3d**), were also formed as a minor product during the synthesis of 2-chloro-4-phenoxyquinolines (**2a**–**2d**); however, the amounts of minor products depended on the substituent and the reaction temperature. The productivity ratio between the 2-chloro-4-phenoxyquinolines (**2a**–**2d**) and the 4-chloro-2-phenoxyquinolines (**3a**–**3d**) under approximately optimal conditions ranged from 5:1 to 12:1 with 67%–83% overall yield in this step. Moreover, 2,4-diphenoxyquinolines (**4a**–**4d**) were synthesized from 2,4-dichloroquinoline when the reaction temperature was set too high at 120 °C, as shown in Scheme 1. 

**Scheme 2:**
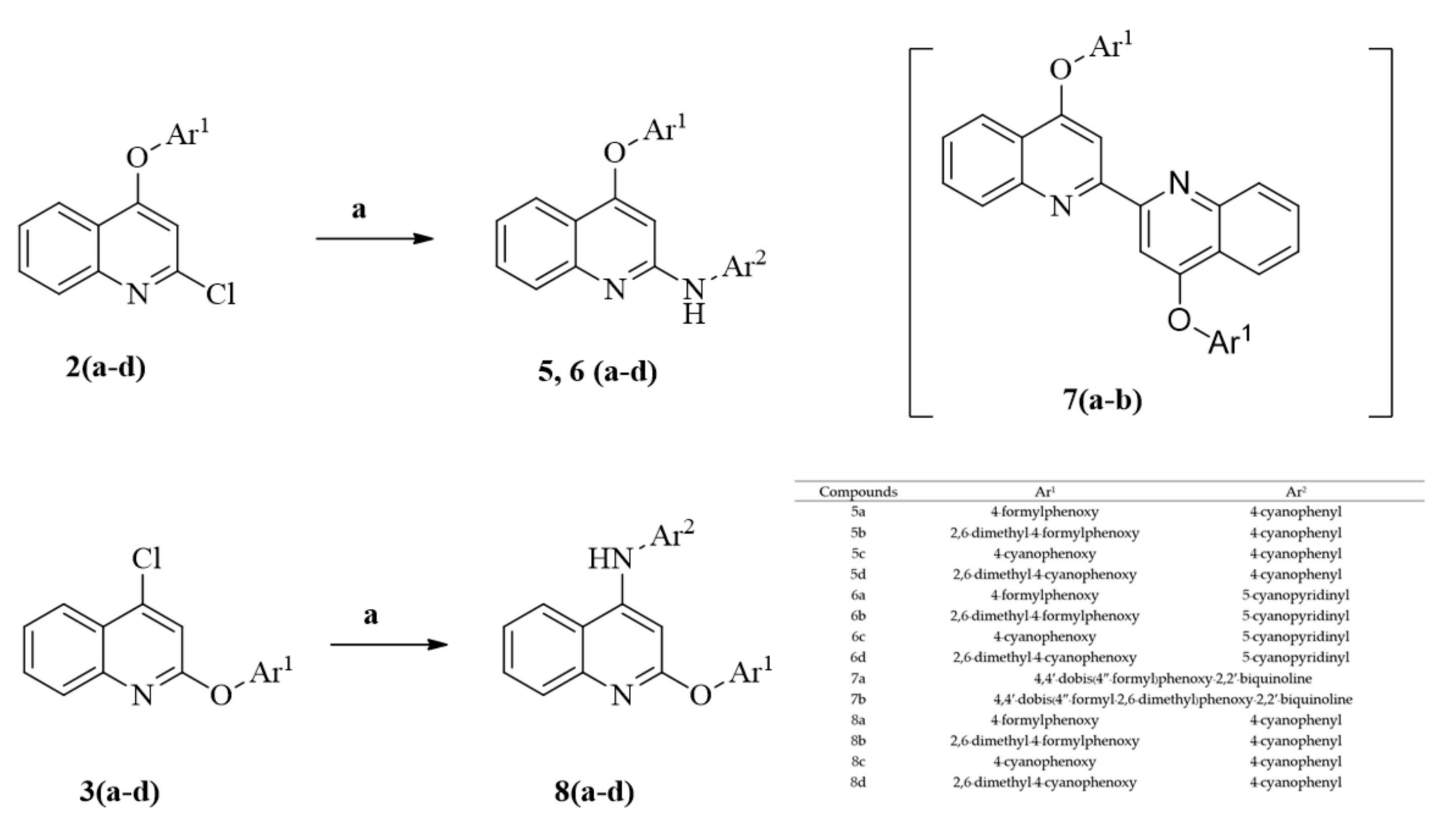
Synthesis of 2-phenylamino-4-phenoxy-quinoline (**5a**–**5d** and **6a**–**6d**), biquinolines (**7a** and **7b**), 2-phenoxy-4-phenylamine-quinoline (**8a**–**8d**). Reagents and conditions: a. 4-aminobenzonitrile (Ar_2_-NH_2_, 1.3 eq.) for **5a**–**5d** and **8a**–**8d** or 2-amino-5-cyanopyridine (Ar_2_-NH_2_) for **6a**–**6d**, Cs_2_CO_3_ (1.5 eq), Pd(OAc)_2_ (5 mol.%), SPhos (5 mol.%), DMF, 120 °C, sealed tube.

**Figure 1:**
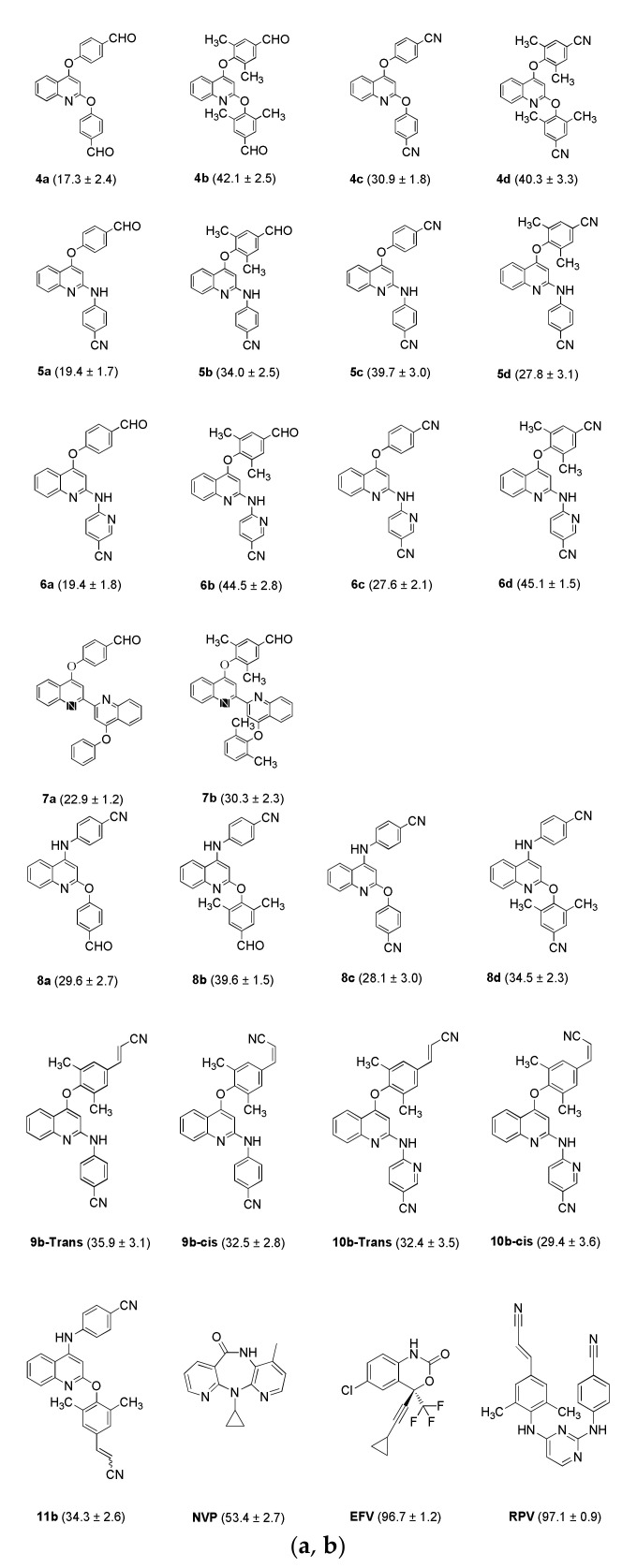
Inhibitory activities (%) of the derivatives and commercially available drugs at 1 µM against HIV-1 RTa. (**a**) The RT kit was commercially available and supplied by Roche, and the data were obtained by standard ELISA. (**b**) Results are expressed as the mean ± standard error of inhibitory activity (%).

**Figure 2:**
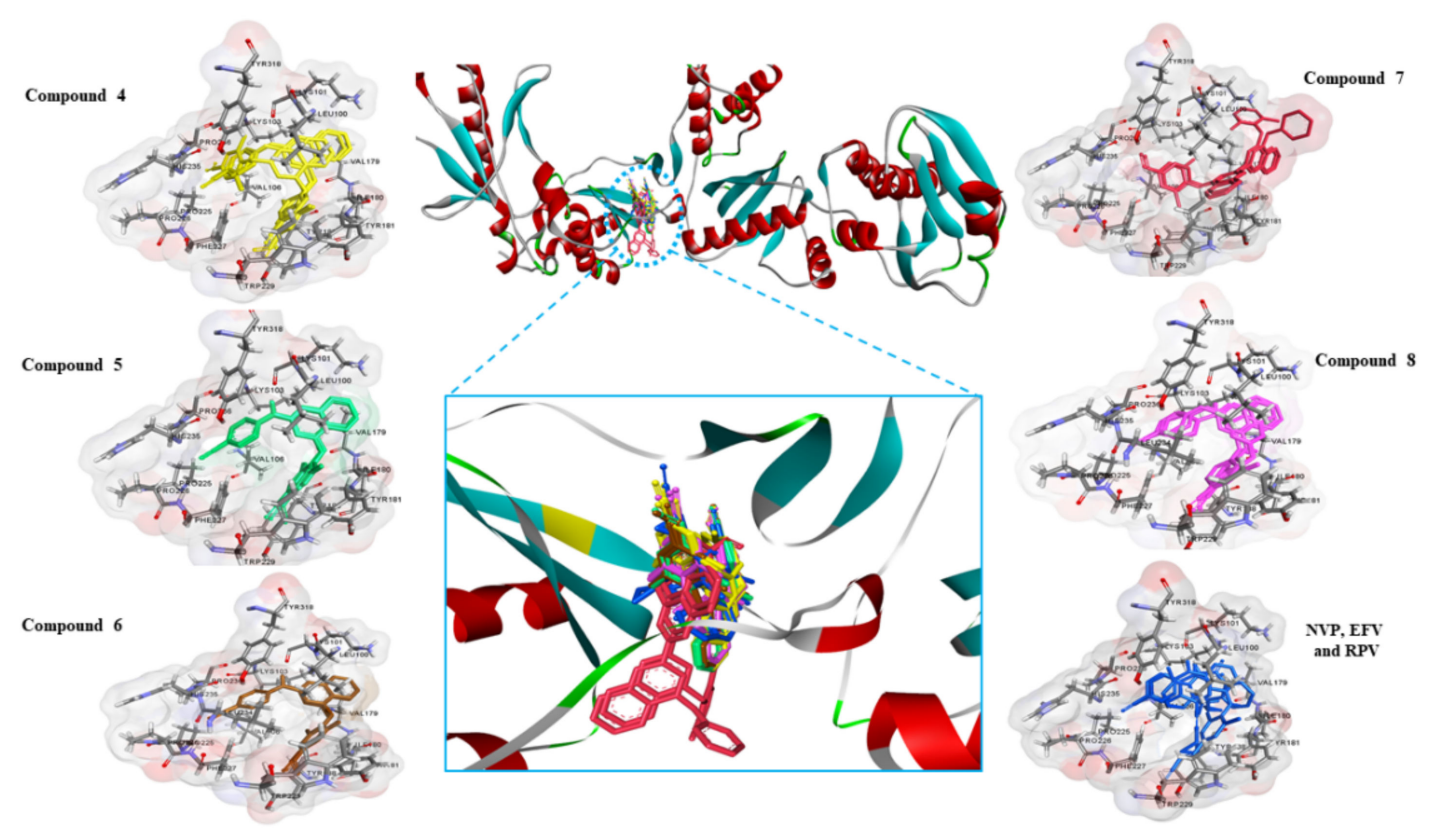
Overlay of the conformations of the derivatives and commercially available drugs in the binding pocket of HIV-1 RT.

Additionally, the 4-chloro-2-phenoxyquinolines (**3a**–**3d**) were coupled with 4-aminobenzonitrile to provide 2-phenoxy-4-phenylaminoquinolines (**8a**–**8d**), according to the reaction conditions as shown in Scheme 2. The percentage inhibitory activities of the synthesized compounds **4a**–**4d** and **8a**–**8d** were tested against HIV-1 RT at 1 µM concentration using reverse transcriptase assay with a colorimetric method. The result is demonstrated in Figure 1. The inhibitory activities (%) of the compounds were similar to those in a previous report for **5a**–**5d** and lower than those of NVP, EFV, and RPV. However, the researcher found that biquinolines, **7a** and **7b**, were formed in tiny amounts during the coupling reactions to synthesize **5a** and **5b** using **2a** and **2b** as the reactants, respectively. This process occurred via dimeric coupling, as shown in Scheme 2. The synthesized compounds **7a** and **7b** were also tested for inhibitory activity against HIV-1 RT at 1 µM concentration. The result was compared with the previously reported values for **5a**–**5d** and is demonstrated in Figure 1. The inhibitory activities (%) of these compounds were lower than those of **5a**–**5d** and NVP, EFV, and RPV. The molecular docking results demonstrated in Table 1 show that biquinolines **7a** and **7b** interacted with the TYR318 residue through hydrogen bonding and interacted with the TYR181 residue through π–π stacking in HIV-1 RT; however, both compounds exhibited a lower number of conformations and were arranged in HIV-1 RT at different positions compared with those for **4a**–**4d**, **5a**–**5d**, and **8a**–**8d**, as shown in Figure 2 (see also Supplementary Materials, Figures S1–S9).

The 2,6-dimethyl group as the substituent in the aromatic side chain at the 4-position of quinoline affected the inhibitory activity compared with other synthesized compounds, namely, **4b**, **4d**, **5b**, **8b**, and **8d**. However, the inhibitory activities (%) implied that the 2,4-diphenoxy substituent of **4a**–**4d** and the 2-phenoxy-4-phenylamino substituent of **8a**–**8d** are not directly related to the effectiveness of their action, because their activities against HIV-1 RT were not much different from the previously reported values for **5a**–**5d**. 

Next, the molecular properties of the ligands were calculated and applied to predict the solubility of the medicated compounds and their possibility of improving the efficiency of action. The lipophilicity of a compound dictates its partition coefficient, which is the value of the tendency of a compound to partition into a nonpolar lipid phase from an aqueous phase. The partition coefficient is an important determinant of medicinal properties and is a rapid and effective tool for assessing initial drug viability [16]. Based on the Lipinski’s rule of five, the compounds can be used as drug candidates if they have a logP value of 0–3, because these values are optimal for the distribution of compounds across cell membranes in the body systems [17,18]. Moreover, the total polar surface area (TPSA) is used to determine the sum of all polar atoms on the surface of molecules, particularly oxygen, nitrogen, and the attached hydrogen atoms, and is applied to predict the optimization of the medicament’s ability to permeate cells. The appropriate molecules that can be used as candidate medicaments have permeability across cell membranes and the blood–brain barrier with TPSA values of less than 140 and 90 Å^2^, respectively [19,20,21]. The calculation result is shown in Table 2. 

**Scheme 3:**
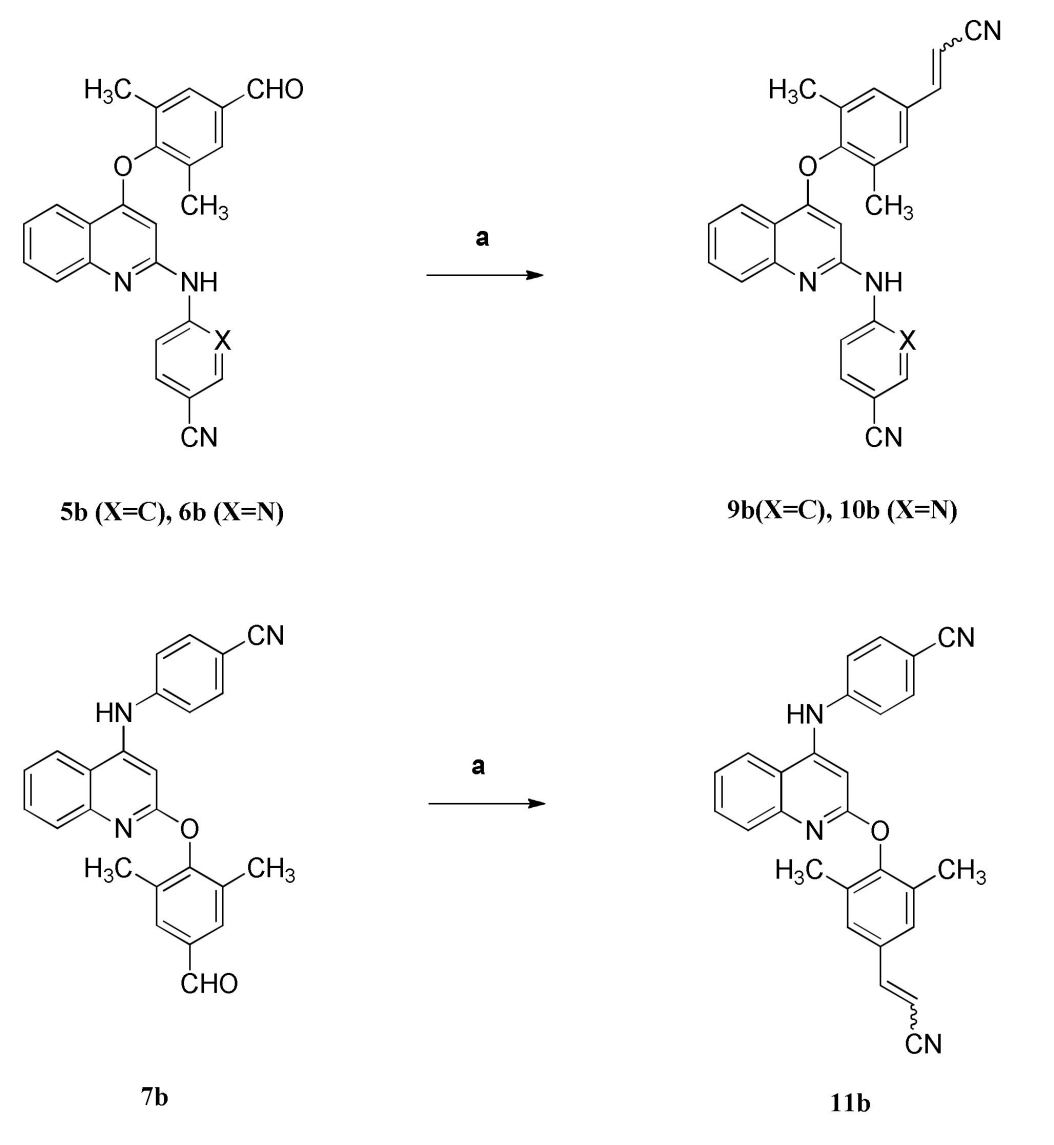
Synthesis of **9b**, **10b**, and **11b**. Reagents and conditions: (a) Potassium tert-butoxide (1.5 eq.), diethyl cyanomethyl phosphonate (1.5 eq.), THF, 0–25 °C.

The results implied that the 2,4-disubstituted quinolines (**4a**–**4d**, **5a**–**5d**, and **8a**–**8d**) and biquinolines (**7a** and **7b**) have logP values higher than 3, which may affect dissolution and absorption. The results of molecular docking analysis revealed that each set of compounds was not completely overlapped, especially **4a**–**4d**, **8a**–**8d**, and **7a**–**7b** (Figure 2, see also Supplementary Materials, Figures S1–S9). Moreover, the number of H-bond donors was 0 for **4a**–**4d**, and the TPSA values of **4a**–**4d**, **8a**–**8d**, **7a**, and **7b** were lower than the previous reported values of **5a**–**5d**. Compounds **4a**–**4d**, **8a**–**8d**, **7a**, and **7b** were not selected for future development because of the evidence mentioned above. At this point, we concluded that these compounds have low hydrophilicity; the logP values were higher than 3 for **4a**–**4d**, **5a**–**5d**, **8a**–**8d**, **7a**, and **7b**, and the TPSA values for **4a**–**4d**, **7a**, and **7b** were lower than 70 Å^2^. Moreover, **5b**, **5c**, and **8b** had moderate activity against HIV-1 RT that was not similar or equal to that of currently available drugs (Figure 1). Next, **6a**–**6d** were designed, and 2-amino-5-cyanopyridine was applied as the substituent at the 2-position instead of 4-aminobenzonitrile in order to increase the number of N atoms in the structure. From the previous study, the molecular docking and interaction results between **5a**–**5d** and HIV-1 RT demonstrated that these compounds aligned in an identical location and bound to HIV-1 RT on Lys101 and His235 residues using hydrogen bonding and on TYR318 residues using π–π stacking in an allosteric site. A study in the literature reported that Lys101 is an amino acid residue with a key importance for the inhibitory activity against HIV-1 RT [22]. The binding energy, the number of conformations, and the interaction between the designed and synthesized compounds and amino acid residues on HIV-1 RT from the molecular docking results (Table 1) revealed similar results for **6a**–**6d** to those for **5a**–**5d**. The calculation results of the pharmacokinetic parameters (Table 2) show that **6a**–**6d** demonstrated logP values in the range of 0–3, lower than those of **4a**–**4d**, **5a**–**5d**, **8a**–**8d**, **7a**, and **7b**, and exhibited TPSA values higher than those of **4a**–**4d**, **5a**–**5d**, **8a**–**8d**, **7a**, and **7b**, which implied that these compounds may tend to perform well in permeating in cell membranes and penetrating in the blood–brain barrier. Moreover, the overlaying of **6a**–**6d** exhibited that these compounds completely overlapped and aligned in the cavity pocket of HIV-1 RT, and the binding area was similar to those of **4a**–**4d**, **5a**–**5d**, and **8a**–**8d** as shown in Figure 2. Then, the 2-pyridinylamino-4-phenyloxyquinoline derivatives **6a**–**6d** were synthesized according to the method for **5a**–**5d**, except that 2-amino-5-cyanopyridine was used for coupling instead of 4-aminobenzonitrile. Compounds **6a**–**6d** were evaluated for their inhibitory activity (%) against HIV-1 RT. Compounds **6b** and **6d** exhibited higher inhibitory activities than **5b** and **5d** when compared with the same substituent function. Compounds **6b** and **6d** exhibited inhibitory activities against HIV-1 RT with inhibition rates of 44.5 ± 2.8 and 45.1 ± 1.5, respectively, at 1 µM concentration; these values were similar to that of NVP (53.4 ± 2.7). Table 1 shows that **6b** and **6c** interacted in the pocket of HIV-1 RT via the LYS101, HIS235, and PRO236 residues using hydrogen bonding and via the TYR188, TRP229, and TYR318 residues using π–π stacking. The numbers of interactions of **6b** and **6d** with HIV-1 RT were higher than those of **4a**–**4d**, **5a**–**5d**, **7a**, **7b**, and **8a**. However, **8a**–**8d** demonstrated moderate efficacy in inhibiting HIV-1 RT and had low productivity in the synthesis process. The IC50 values of **6b** and **6d** were then deter-mined. The results of inhibitory analysis of **6b** and **6d** compared with the currently available drugs are presented in Table 3. Compounds **6b** and **6d** exhibited IC50 values of 1.93 and 1.22 µM, respectively, similar to that of NVP (IC50 = 1.05 µM). RPV is an NNRTI that is usually used for HAART in patients with HIV infection. RPV has a high specificity against HIV-1 RT with low cytotoxicity in humans [23]. In our research, RPV was one of the three structures used in the molecular hybridization approach to evaluate novel compounds as potent NNRTIs. The cyanovinyl substituent, which was the same as the side chain in the structure of RPV, was designed and applied to **5b**, **6b**, and **8b**. Then, the synthesized compounds **9b**, **10b**, and **11b** were obtained from **5b**, **6b**, and **8b**, respectively, using diethyl cyanomethyl phosphonate in basic conditions, as demonstrated in Scheme 3. The products were obtained in 56%–64% yield from **5b**, **6b**, and **8b** and consisted of trans- and cis-forms in a ratio of 7:3. However, the mixture of **11b** could not be separated into each form. Compounds **9b**, **10b**, and **11b** showed lower inhibitory activities against HIV-1 RT compared with **5b**, **6b**, and **8b**. Moreover, the inhibitory activities of the trans- and cis- forms were not much different. In this studies, the binding energy values between designed compounds and HIV-1 RT as shown in Table 1 demonstrated that the calculated binding energy of NVP is found to be higher than that of RPV because the size of the NPV core structure is larger than the binding pocket of RPV in the HIV-1 RT enzyme. This could lead to the repulsive interactions inside the binding pocket and eventually increasing the binding energy. In the case of the de-signed compounds, their quinoline-based core structures are slightly bigger than that of RPV but they still fit in very well with the binding pocket. Besides, the core structure and the side chains of the designed compounds can also interact very well with amino acid residues inside the HIV-1 RT binding pocket. As a result, their binding energy with the enzyme is lower or comparable to that of RPV. Although EFV displays a similar size of core structure to the synthesized compounds, they have different substituents which could lead to different interactions and binding energy. As shown, EFV only exerts one H-bond with the amino acid residue in the HIV-1 RT pocket. Therefore, its binding energy is found to be higher than that of the synthesized compounds. It is worth mentioning that the obtained binding energy is solely predicted based on the calculation via a molecular docking technique. Further examination of the biological activity and the effect of 2,4-disubstituents of the synthesized compounds are still needed. 

Additionally, **4**–**8**(**a**–**d**), (**9**–**11**)**b**, and the three common drugs (NVP, EFV, and RPV) were evaluated for their cytotoxicity against various cancer cell lines, as shown in Table 4 and Table 5. In the literature, NNRTIs, namely, NVP, EFV, and RPV, have been report-ed as being toxic against a wide range of cancer cells in vitro [24,25,26] but only have minor toxicity against normal tissue cells [24]. The toxicity of NNRTIs against cancer cells promoted the idea that these drugs can be used to prevent or even treat HIV-1 infection and cancer. All of the synthesized compounds that showed activity against HIV-1 RT were cytotoxic to cancer cell lines. The results of the cytotoxicity assay revealed that **6b**, EFV, and RPV had strong activities against acute lymphoblastic leukemia (MOLT-3) cells (IC50 = 12.7 ± 1.1, 24.6 ± 1.2, and 4.3 ± 0.5 µM, respectively), cervical carcinoma (HeLA) cells (IC50 = 25.7 ± 0.8, 46.2 ± 1.3, and 11.3 ± 1.2 µM, respectively), and promyeloblast (HL-60) cells (IC50 = 20.5 ± 4.1, 33.7 ± 1.1, and 11.5 ± 0.8 µM, respectively). Compounds **6b**, EFV, and RPV had substantial cytotoxicity against the cancer cell lines, whereas **6b** and RPV had very low cytotoxicity against normal embryonic lung (MRC-5) cells. However, **6d** had high inhibitory activity with a very low IC50 value against HIV-1 RT and had no cytotoxicity against normal embryonic lung (MRC-5) cells compared with EFV and RPV. All of the evidence brought us to the conclusion that the substituents of quinoline at the 4-position, namely, 2′,6′-dimethyl-4′-cyanophenoxy and 2′,6′-dimethyl-4′-formylphenoxy, and the substituent at the 2-position, namely, 5″-cyanopyridin-2″ylamino, had inhibitory activities against HIV-1 RT. Thus, **6b** and **6d** are candidates for the development of HIV treatment in the near future. 

## 3. Materials and Methods

### 3.1. Materials

The starting materials and other reagents for synthesis were purchased from Aldrich Company and Tokyo Chemical Industry and used as received without further purification unless otherwise indicated. The melting points were measured using an SMP3 StuartTM digital melting point apparatus from Bibby Sterlin, Ltd (Stone, United Kingdom). All synthesized compounds were analyzed using nuclear magnetic resonance (NMR) and mass spectrometry to confirm their structures. Proton and carbon NMR spectra were accomplished using the Bruker Avance III HD 300 spectrometer at 75, 100, 300, and 400 MHz. High-resolution mass spectra were measured with a micrOTOF electrospray ionization time-of-flight mass spectrometer (Bruker Daltonics, Germany). HepG2 (hepatocarcinoma), MOLT-3 (acute lymphoblastic leukemia), HuCCA-1 (cholangiocarcinoma), A549 (lung carcinoma), MRC-5 (normal embryonic lung cell), MDA-MB-231 (hormone-independent breast cancer), S102 (Thai liver cancer), HeLA (cervical carcinoma), T47-D (hormone-dependent breast cancer), H69AR (lung cancer, multidrug resistant), and HL-60 cell lines (promyeloblast) were purchased from Hyclone Laboratories. Doxorubicin (purity ≥98%) and etoposide (purity 98%) were purchased from Aldrich Company.

### 3.2. Molecular Docking Studies

The crystal structure of HIV-1 (4G1Q) [27] was obtained from the Protein Data Bank. 4G1Q is a crystal structure of HIV-1 RT in a complex with RPV, which is a commercial NNRTI drug. The protein was prepared by removing the water molecules, ligand, and other unnecessary small molecules from the crystal structure of the ligand–HIV-1 RT complex (PDB code: 4G1Q) before molecular docking. The geometry of quinoline derivatives, **4**–**7**(**a**–**d**) and **8**–**10**(**b**), was fully optimized using the density functional theory at B3LYP/6-31G (d, p) level implemented in Gaussian 09 [28]. The binding inter-actions of 2,4-diphenoxyquinoline (**4**[**a**–**d**]), 2-phenylamino-4-phenoxy-quinoline (**5**–**6**[**a**–**d**]), 4,4′-di-(4′-formylphenoxy)-2,2′-biquinoline (**7a**), 4,4′-di-(2′,6′-dimethyl-4′-formylphenoxy)-2,2′-biquinoline (**7b**), and 2-phenoxy-4-phenylamine-quinoline (**8** [**a**-**d**], **9**–**11**[**b**]) with HIV-1 RT were simulated by molecular docking using AutoDock 4.2 [29]. Default AutoDock settings were used; the population size for the Lamarckian genetic algorithm was set to 150 individuals, and the number of genetic algorithm was set to 200. The maximum number of evaluations was 2,500,000, and the maximum number of generations was 27,000. Blind docking was carried out using a grid box with a size of 80 × 80 × 80 A° along the x, y, and z axes, respectively, to observe the binding sites of inhibitors in HIV-1 RT. The grid center for HIV-RT was located at x = 49.082, y = −28.29, and z = 37.541, and the spacing was 0.375 A°. Discovery Studio 4.0 software was applied to visualize the lowest energy conformation. The identification of ligand binding modes was concluded by iteratively evaluating a number of ligand conformations and estimating the energy of their inter-actions with the target. All experiments were accomplished in triplicate, and the replicates showed similar results. 

### 3.3. Pharmacokinetic Parameter Calculation

Pharmacokinetic parameters are used to predict the various characteristics of the medicament in the body system model. Pharmacokinetics parameters have four main types, namely: absorption, distribution, metabolism, and excretion (ADME) [30,31]. In this study, SwissADME was used to study the pharmacokinetic parameters. 2,4-Diphenoxyquinoline (**4**[**a**–**d**]), 2-phenylamino-4-phenoxy-quinoline (**5**–**6**[**a**–**d**]), 4,4′-di-(4′-formylphenoxy)-2,2′-biquinoline (**7a**), 4,4′-di-(2′,6′-dimethyl-4′-formylphenoxy)-2,2′-biquinoline (**7b**), and 2-phenoxy-4-phenylamine-quinoline (**8**[**a**–**d**], **9**–**11**[**b**]) were considered as ligands. The pharmacokinetic parameters of the ligands were accomplished by SwissADME tool [32]. Drug-likeness and molecular property prediction were analyzed depending on the Lipinski’s rule of five [33]. 

### 3.4. Synthesis

General procedure for the preparation of 2-chloro-4-phenoxyquinoline (**2a–2d**). A mixture of 2,4-dichloroquinoline (1, 10 mmol) and hydroxyl benzene (11 mmol) in di-methylformamide (DMF, 30 mL) with anhydrous cesium carbonate (Cs_2_CO_3_, 20 mmol) was heated in a sealed tube, stirred at 80 °C for 16 h, and cooled. Then, the mixture was poured into ice-water and extracted thrice with ethyl acetate. The combined organic layers were washed with saturated NaCl and dried over Na_2_SO4. The crude product was purified on a silica gel column (eluent: hexane/ethyl acetate) to obtain **2a**–**2d** with 62%–69% yield. However, 4-chloro-2-phenoxyquinoline (**3a**–**3d**) was also found during the reaction progress as a side product with 5%–14% yield.

General procedure for the preparation of 2,4-diphenoxyquinoline (**4a–4d**). A mixture of 1 (10 mmol) and hydroxyl benzene (21 mmol) in DMF (30 mL) with anhydrous Cs_2_CO_3_ (20 mmol) was heated in a sealed tube, stirred at 120 °C for 8–16 h, and cooled. Afterward, the mixture was poured into ice-water and extracted thrice with ethyl acetate. The combined organic layers were washed with saturated NaCl and dried over Na_2_SO_4_. The crude product was purified on a silica gel column (eluent: hexane/ethyl acetate) to obtain **4a**–**4d** with 55%–65% yield. 

General procedure for the preparation of 2-phenylamino-4-phenoxy-quinoline (**5a–5d** and **6a–6d**). A mixture of **2a**–**2d** (0.5 mmol), 4-aminobenzonitrile (0.65 mmol) for the synthesis of **5a**–**5d** or 2-amino-5-cyanopyridine (0.65 mmol) for the synthesis of **6a**–**6d**, Pd(OAc)_2_ (0.05 mmol), SPhos (0.05 mmol), and Cs_2_CO_3_ (0.75 mmol) in DMF (20 mL) was stirred, heated at 120 °C for 5–10 h, and cooled. Then, the corresponding solution was evaporated in vacuo. The residue was purified on a silica gel column (eluent: hexane/ethyl acetate) to obtain 5–6(a–d) with 59%–72% yield. The dimers of biquinoline, namely, 4,4′-di-(4′-formylphenoxy)-2,2′-biquinoline (**7a**) and 4,4′-di-(2′,6′-dimethyl-4′-formylphenoxy)-2,2′-biquinoline (**7b**), were found during the syntheses of **5a**, **5b**, **6a**, and **6b**. 

General procedure for the preparation of 2-phenoxy-4-phenylamine-quinoline (**8a–8d**). A mixture of 4-chloro-2-phenoxyquinoline **3a**–**3d** (0.5 mmol), 4-aminobenzonitrile (0.65 mmol), Pd(OAc)_2_ (0.05 mmol), SPhos (0.05 mmol), and Cs_2_CO_3_ (0.75 mmol) in DMF (20 mL) was stirred, heated at 120 °C for 6 h, and cooled. The corresponding solution was evaporated in vacuo. The residue was purified on a silica gel column (eluent: hexane/ethyl acetate) to obtain **8a**–**8d**) with 57%–66% yield.

General procedure for the preparation of **9b**, **10b**, and **11b**. Potassium tert-butoxide (1.50 mmol) was added to an ice-cooled solution of diethyl cyanomethyl phosphonate (1.50 mmol) in THF (20 mL). The mixture was stirred at 0 °C for 30 min and then at room temperature for another 30 min. A solution of 4-(4′-formylphenoxy)-2-arylamino-quinoline (**5b** and **6b**) or 2-(4′-formylphenoxy)-4-phenylamino-quinoline (**8b**, 1 mmol) in THF (13 mL) was added dropwise to the reaction mixture. The solution was continued for 8–10 h. After the reaction was completed, the corresponding solution was added with water and extracted with ethyl acetate. The organic layer was dried over Na_2_SO_4_ and concentrated under reduced pressure. The residue was purified on a silica gel column (eluent: hexane/ethyl acetate) to obtain **9b**, **10b**, and **11b** with 56%–64% yield. The E:Z isomer ratio for **9b** and **10b** was 7:3, whereas **11b** was inseparable. 

4-(4′-formylphenoxy)-2-chloroquinoline (**2a**) With a 67% yield, the synthesis started with 0.30 g (1.51 mmol) of **1** to obtain 0.29 g of **2a**, which consisted of white solid and m.p. 113.9–114.2 °C. The 1H-NMR (300 MHz, CDCl3): 6.64 (s, 1H, ArH-3), 7.35 (d, 2H, *J* = 8.7 Hz, ArH-2′, 6′), 7.61 (td, 1H, *J* = 7.8, 1.2 Hz, ArH-6), 7.81 (td, 1H, *J* = 7.8, 1.2 Hz, ArH-7), 8.04 (m, 3H, ArH-8, 3′, 5′), and 8.26 (dd, 1H, *J* = 8.4, 0.9 Hz, ArH-5), and 10.1 (s, 1H, CHO). The 13C-NMR (75 MHz, CDCl3): 106.5, 120.4, 121.0, 121.8, 126.9, 128.5, 131.6, 132.3, 134.0, 149.0, 151.0, 159.1, 161.9, and 190.5. Finally, the HRMS (+ESI) was C_16_H_11_ClNO_2_ [M + H] +; it requires 284.0478, but has 284.0465. 

2-(4′-formylphenoxy)-4-chloroquinoline (**3a**) obtained as the side product with an 11% yield (47.2 mg), which consisted of white solid and m.p. 121.7–122.5 °C. The 1H-NMR (300 MHz, CDCl3): 7.26 (s, 1H, ArH-3), 7.41 (dd, 2H, *J* = 8.6, 1.8 Hz, ArH-2′, 6′), 7.53 (td, 1H, *J* = 7.6, 1.2 Hz, ArH-6), 7.68 (td, 1H, *J* = 7.7, 1.4 Hz, ArH-7), 7.79 (dd, 1H, *J* = 8.4, 0.5 Hz, ArH-8), 7.95 (dt, 2H, *J* = 8.6, 2.4 Hz, ArH-3′, 5′), 8.16 (dd, 1H, *J* = 8.3, 1.0 Hz, ArH-5), and 10.0 (s, 1H, CHO). The 13C-NMR (75 MHz, CDCl3): 112.9, 121.6, 124.0, 124.2, 126.1, 128.2, 131.0, 131.4, 133.1, 145.2, 146.4, 158.5, 159.9 and 190.9. Finally, the HRMS (+ESI) was C_16_H_11_ClNO_2_ [M + H] +; it requires 284.0478, but has 284.0468. 

4-(2′,6′-Dimethyl-4′-formylphenoxy)-2-chloroquinoline (**2b**) With a 69% yield, the synthesis started with 1.0 g (5.05 mmol) of **1** to obtain 1.09 g of **2b**, which consisted of a white solid and m.p. 154.6–156.9 °C. The 1H-NMR (300 MHz, CDCl3): 2.23 (s, 6H, ArCH_3_-2′, 6′), 6.20 (s, 1H, ArH-3), 7.65 (td, 1H, *J* = 7.5, 1.2 Hz, ArH-6), 7.75 (s, 2H, ArH-3′, 5′), 7.82 (td, 1H, 7.5, 1.2 Hz, ArH-7), 8.04 (dd, 1H, 8.3, 0.9 Hz, ArH-8), and 8.4 (dd, 1 H, 8.3, 0.9 Hz, ArH-5), and 10.0 (s, 1H, CHO). The 13C-NMR (75 MHz, CDCl3): 16.1, 102.9, 119.5, 121.7, 126.7, 128.5, 131.5, 132.1, 134.4, 148.8, 151.3, 154.6, 161.0, and 191.2. Finally, the HRMS (+ESI) was C_18_H_15_ClNO_2_ [M + H] +; it requires 312.0791, but has 312.0791. 

2-(2′,6′-Dimethyl-4′-formylphenoxy)-4-chloroquinoline (**3b**) obtained as the side product with a 14% yield (0.22 g), which consisted of white solid and m.p. 113.7–114.2 °C. The 1H-NMR (300 MHz, CDCl_3_): 2.21 (s, 6H, ArCH3-2′, 6′), 7.27 (d, 1H, *J* = 5.8 Hz, ArH-3), 7.50 (td, 1H, *J* = 7.4, 1.7 Hz, ArH-6), 7.60-7.70 (m, 4H, ArH-7, 8, 3′, 5′), 8.15 (dd, 1H, *J* = 8.5, 0.8 Hz, ArH-5), and 9.99 (s, 1H, CHO). The 13C-NMR (75 MHz, CDCl_3_): 16.7, 111.5, 123.8, 124.0, 125.6, 128.2, 130.3, 130.9, 132.5, 133.7, 145.2, 146.9, 155.3, 159.7 and 191.7. Finally, the HRMS (+ESI) was C_18_H_15_ClNO_2_ [M + H] +; it requires 312.0791, but has 312.0777. 

4-(4′-cyanophenoxy)-2-chloroquinoline (**2c**) With a 60% yield, the synthesis started with 0.30 g (1.51 mmol) of **1** to obtain 0.26 g of **2c**, which consisted of white solid and m.p. 214.1–215.0 °C. The 1H-NMR (300 MHz, CDCl_3_): 6.63 (s, 1H, ArH-3), 7.32 (d, 2H, *J* = 8.7 Hz, ArH-2′, 6′), 7.60 (t, 1H, *J* = 7.2 Hz, ArH-6), 7.78-7.83 (m, 3H, ArH-7, 3′, 5′), 8.02 (d, 1H, *J* = 8.4 Hz, ArH-8), and 8.22 (d, 1H, *J* = 8.4 Hz, ArH-5). The 13C-NMR (75 MHz, CDCl_3_): 106.4, 109.6, 117.9, 119.6, 120.2, 121.2, 121.6, 126.9, 128.4, 131.5, 134.4, 134.7, 148.8, 150.8, 157.6, and 161.5. Finally, the HRMS (+ESI) was C_16_H_10_ClN_2_O [M + H] +; it requires 281.0482, but has 281.0470. 

2-(4′-cyanophenoxy)-4-chloroquinoline (**3c**) obtained as the side product with a 9% yield (38.3 mg), which consisted of white solid and m.p. 147.0–148.0 °C. The 1H-NMR (300 MHz, CDCl_3_): 7.27 (d, 1H, *J* = 5.7 Hz, ArH-3), 7.39 (dt, 2H, *J* = 8.4, 0.8 Hz, ArH-2′, 6′), 7.56 (td, 1H, *J* = 7.3, 1.3 Hz, ArH-6), 7.65-7.82 (m, 4H, ArH-7, 8, 3′, 5′), and 8.18 (dd, 1H, *J* = 8.1, 0.9 Hz, ArH-5). The 13C-NMR (75 MHz, CDCl_3_): 108.4, 112.8, 118.6, 122.1, 124.1, 124.3, 126.3, 128.2, 131.1, 133.8, 145.4, 146.4, 156.9, and 159.7. Finally, the HRMS (+ESI) was C_16_H_10_ClN_2_O [M + H] +; it requires 281.0482, but has 281.0475. 

4-(2′,6′-Dimethyl-4′-cyanophenoxy)-2-chloroquinoline (**2d**) With a 62% yield, the synthesis started with 0.30 g (1.51 mmol) of **1** to obtain 0.29 g of **2d**, which consisted of white solid and m.p. 184.3–185.0 °C. The 1H-NMR (300 MHz, CDCl3): 2.20 (s, 6H, ArCH3-2′, 6′), 6.20 (s, 1H, ArH-3), 7.53 (s, 2H, ArH-3′, 5′), 7.65 (td, 1H, *J* = 9.2, 1.5 Hz, ArH-6), 7.83 (td, 1H, *J* = 9.2, 1.5 Hz, ArH-7), 8.04 (dd, 1H, *J* = 8.3, 1.2 Hz, ArH-8), and 8.38 (dd, 1H, *J* = 8.3, 1.2 Hz, ArH-5). The 13C-NMR (75 MHz, CDCl3): 15.9, 102.7, 110.4, 118.1, 119.3, 121.6, 126.8, 128.4, 131.5, 132.7, 133.3, 148.8, 151.1, 153.2, and 160.7. Finally, the HRMS (+ESI): C_18_H_14_ClN_2_O [M + H] +; it requires 309.0795, but has 309.0783. 

2-(2′,6′-Dimethyl-4′-cyanophenoxy)-4-chloroquinoline (**3d**) obtained as the side product with a 5% yield (23.4 mg), which consisted of white solid and m.p. 152.2–153.0 °C. The 1H-NMR (300 MHz, CDCl_3_): 2.16 (s, 6H, ArCH3-2′, 6′), 7.29 (s, 1H, ArH-3), 7.44 (s, 2H, ArH-3′, 5′), 7.51 (td, 1H, *J* = 7.3, 2.2 Hz, ArH-6), 7.60–7.69 (m, 2H, ArH-7, 8) and 8.16 (d, 1H, *J* = 8.2 Hz, ArH-5). The 13C-NMR (75 MHz, CDCl_3_): 16.6, 109.1, 111.5, 118.9, 123.9, 124.0, 125.7, 128.1, 130.9, 132.5, 133.0, 145.3, 146.7, 153.9, and 159.4. Finally, the HRMS (+ESI): C_18_H_14_ClN_2_O [M + H] +; it requires 309.0795, but has 309.0787. 

2,4-di-(4′-formylphenoxy)-quinoline (**4a**) With a 60% yield, the synthesis started with 50.0 mg (0.25 mmol) of **1** to obtain 55.9 mg of **4a**, which consisted of white solid and m.p. 164.9–165.5 °C. The 1H-NMR (300 MHz, CDCl_3_): 6.43 (s, 1H, ArH-3), 7.36–7.43 (m, 4H, ArH-2′, 6′, 2″, 6″), 7.51 (td, 1H, *J* = 7.6, 1.3 Hz, ArH-6), 7.71 (td, 1H, *J* = 8.1, 1.5 Hz, ArH-7), 7.80 (dd, 1H, *J* = 8.4, 0.5 Hz, ArH-8), 7.94 (ddd, 2H, *J* = 8.7, 2.0 Hz, ArH-3′, 5′), 8.03 (ddd, 2H, *J* = 8.7, 2.0 Hz, ArH-3″, 5″), 8.22 (dd, 1H, *J* = 8.3, 0.9 Hz, ArH-5), 10.00 (s, 1H, CHO), and 10.04 (s, 1H, CHO). The 13C-NMR (75 MHz, CDCl_3_): 97.2, 119.7, 120.9, 121.5, 121.7, 125.3, 127.9, 131.1, 131.4, 132.2, 132.9, 133.7, 147.3, 158.7, 159.5, 161.3, 163.2, 190.5, and 191.0. Finally, the HRMS (+ESI) was C_23_H_16_NO_4_ [M + H] +; it requires 370.1079, but has 370.1088. 

2,4-di-(2′,6′-Dimethyl-4′-formylphenoxy)-quinoline (**4b**) With a 65% yield, the synthesis started with 50 mg (0.25 mmol) of **1** to obtain 69.7 mg of **4b**, which consisted of white solid and m.p. 120.5–121.0 °C. The 1H-NMR (300 MHz, CDCl3): 2.16 (s, 6H, ArCH_3_-2′, 6′), 2.29 (s, 6H, ArCH_3_-2″, 6″), 5.99 (s, 1H, ArH-3), 7.46–7.51 (m, 1H, ArH-6), 7.63–7.66 (m, 4H, ArH-3′, 5′, 7, 8), 7.76 (s, 2H, ArH-3″, 5″), 8.35 (d, 1H, *J* = 8.1 Hz, ArH-5), 9.95 (s, 1H, CHO) and 10.02 (s, 1H, CHO). The 13C-NMR (75 MHz, CDCl_3_): 16.1, 16.7, 91.7, 118.5, 121.6, 124.5, 127.7, 130.2, 130.7, 130.9, 132.2, 132.4, 133.4, 134.2, 147.5, 155.0, 155.6, 161.3, 162.1, 191.3, and 191.7. Finally, the HRMS (+ESI) was C_27_H_24_NO_4_ [M + H] +; it requires 426.1700, but has 426.1690. 

2,4-di-(4′-cyanophenoxy)-quinoline (**4c**) With a 55% yield, the synthesis started with 50 mg (0.25 mmol) of **1** to obtain 50.4 mg of **4c**, which consisted of white solid and m.p. 220.9–221.5 °C. The 1H-NMR (300 MHz, CDCl_3_): 6.40 (s, 1H, ArH-3), 7.30–7.40 (m, 4H, ArH-2′, 6′, 2″, 6″), 7.51 (td, 1H, *J* = 7.5, 1.3 Hz, ArH-6), 7.66–7.75 (m, 3H, ArH-7, 3′, 5′), 7.75–7.84 (m, 3H, ArH-8, 3″, 5″) and 8.18 (dd, 1H, *J* = 8.0, 0.7 Hz, ArH-5). The 13C-NMR (75 MHz, CDCl3): 97.1, 108.2, 109.4, 118.0, 118.6, 119.6, 121.3, 121.6, 122.0, 125.5, 127.8, 131.3, 133.7, 134.7, 147.2, 157.0, 158.1, 161.0, and 163.0. Finally, the HRMS (+ESI) was C_23_H_14_N_3_O_2_ [M + H] +; it requires 364.1086, but has 364.1067.

2,4-di-(2′,6′-Dimethyl-4′-cyanophenoxy)-quinoline (**4d**) With a 57% yield, the synthesis started with 50 mg (0.25 mmol) of **1** to obtain 60.3 g of **4d**, which consisted of white solid and m.p. 201.8–202.4 °C. The 1H-NMR (300 MHz, CDCl_3_): 2.10 (s, 6H, ArCH_3_-2′, 6′), 2.24 (s, 6H, ArCH_3_-2″, 6″), 5.96 (s, 1H, ArH-3), 7.41 (s, 2H, ArH-3′, 5′), 7.46–7.52 (m, 1H, ArH-6), 7.54 (bs, 2H, ArH-3″, 5″), 7.64–7.66 (m, 2H, ArH-7, 8), and 8.32 (d, 1H, *J* = 8.3 Hz, ArH-5). The 13C-NMR (75 MHz, CDCl_3_): 16.0, 16.5, 91.6, 102.8, 108.9, 110.2, 118.3, 118.4, 119.0, 121.5, 121.6, 124.7, 126.8, 127.8, 128.5, 130.9, 131.6, 132.4, 132.8, 133.0, 133.3, 147.4, 153.7, 154.1, 161.0, and 162.0. Finally, the HRMS (+ESI) was C_27_H_22_N_3_O_2_ [M + H] +; it requires 420.1706, but has 420.1712. 

4-(4′-formylphenoxy)-2-(4″-cyanophenyl)-aminoquinoline (**5a**) With a 62% yield, the synthesis started with 70.0 mg (0.25 mmol) of **2a** to obtain 55.9 mg of **5a**, which consisted of white solid and m.p. 267.1–268.0 °C. **5a** was obtained in 41.5% overall yield (2 steps from **1**). The 1H-NMR (300 MHz, DMSO-d6): 6.42 (s, 1H, ArH-3), 7.43 (t, 1H, *J* = 7.1 Hz, ArH-6), 7.50-7.59 (m, 2H, ArH-2″, 6″), 7.66-7.88 (m, 4H, ArH-3″, 5″, 2′, 6′), 8.04–8.15 (m, 5H, ArH-5, 7, 8, 3′, 5′), 9.80 (bs, 1H, NH), and 10.10 (s, 1H, CHO). The 13C-NMR (75 MHz, DMSO-d6): 97.8, 102.0, 117.6, 117.9, 119.6, 121.2, 123.6, 126.7, 130.7, 132.1, 133.1, 133.5, 145.4, 148.1, 154.1, 159.1, 160.4, and 191.9. Finally, the HRMS (+ESI) was C_23_H_16_N_3_O_2_ [M + H] +; it requires 366.1237, but has 366.1229. 

4-(2′,6′-Dimethyl-4′-formylphenoxy)-2-(4″-cyanophenyl)-aminoquinoline (**5b**) With a 69% yield, the synthesis started with 40.0 mg (0.13 mmol) of **2b** to obtain 34.8 mg of **5b**, which consisted of white solid and m.p. 291.3–292.2 °C. **5b** was obtained in 47.6% overall yield (2 steps from **1**). The 1H-NMR (300 MHz, CDCl_3_): 2.26 (s, 6H, ArCH_3_-2′, 6′), 5.73 (s, 1H, ArH-3), 6.95 (bs, 1H, NH), 7.46 (td, 1H, *J* = 7.6, 1.0 Hz, ArH-6), 7.56 (m, 2H, ArH-2″, 6″), 7.70 (s, 2H, ArH-3′, 5′), 7.74 (m, 1H, ArH-7), 7.87 (m, 2H, ArH-3″, 5″), 7.91 (d, 1H, *J* = 8.6 Hz, ArH-8), 8.30 (dd, 1H, *J* = 8.2, 1.1 Hz, ArH-5), and 9.96 (s, 1H, CHO). The 13C-NMR (75 MHz, CDCl_3_): 16.1, 93.1, 104.0, 117.6, 118.2, 119.5, 121.5, 123.9, 127.3, 130.9, 132.5, 133.2, 134.1, 144.6, 148.7, 153.5, 155.2, 160.7, and 191.4. Finally, the HRMS (+ESI) was C_25_H_20_N_3_O_2_ [M + H] +; it requires 394.1550, but has 394.1559. 

4-(4′-cyanophenoxy)-2-(4″-cyanophenyl)-aminoquinoline (**5c**) With a 59% yield, the synthesis started with 70.0 mg (0.25 mmol) of **2c** to obtain 53.3 mg of **5c**, which consist-ed of white solid and m.p. 278.5–278.7 °C. **5c** was obtained in 35.4% overall yield (2 steps from **1**). The 1H-NMR (300 MHz, CDCl_3_): 6.59 (s, 1H, ArH-3), 7.17-7.31 (m, 4H, ArH-2″, 6″, 2′, 6′), 7.47–7.54 (m, 1H, ArH-6), 7.58–7.80 (m, 7H, ArH-7, 8, 3″, 5″, 3′, 5′, NH), and 8.08 (d, 1H, *J* = 7.9 Hz, ArH-5). The 13C-NMR (75 MHz, CDCl3): 103.9, 108.5, 118.1, 119.8, 119.9, 121.6, 125.9, 126.9, 128.0, 131.1, 133.2, 134.4, 147.8, 148.7, 156.0, 158.7, and 160.2. Finally, the HRMS (+ESI) was C_23_H_15_N_4_O [M + H] +; it requires 363.1240, but has 363.1232. 

4-(2′,6′-Dimethyl-4′-cyanophenoxy)-2-(4″-cyanophenyl)-aminoquinoline (**5d**) With a 60% yield, the synthesis started with 70.0 mg (0.23 mmol) of **2c** to obtain 53.0 mg of **5d**, which consisted of white solid and m.p. 256.5–257.0 °C. **5d** was obtained in 37.2% overall yield (2 steps from **1**). The 1H-NMR (300 MHz, CDCl_3_): 2.23 (s, 6H, ArCH_3_-2′, 6′), 5.81 (s, 1H, ArH-3), 7.41–7.46 (m, 1H, ArH-6), 7.47 (s, 2H, ArH-3′, 5′), 7.56 (dd, 2H, *J* = 7.1, 1.8 Hz, ArH-2″, 6″), 7.71 (m, 2H, ArH-7, NH), 7.87-7.99 (m, 3H, ArH-8, 3″, 5″), and 8.27 (dd, 1H, *J* = 8.2, 1.1 Hz, ArH-5). The 13C-NMR (75 MHz, CDCl_3_): 16.0, 93.3, 103.6, 109.4, 117.4, 118.2, 119.6, 121.3, 123.8, 127.2, 130.9, 133.0, 133.1, 133.2, 144.8,148.6, 153.8, 154.0, and 160.2. Finally, the HRMS (+ESI) was C_25_H_19_N_4_O [M + H] +; it requires 391.1553, but has 391.1548. 

4-(4’-formylphenoxy)-2-(5″-cyanopyridin-2″ylamino)quinoline (**6a**) With a 60% yield, the synthesis started with 70 mg (0.25 mmol) of **2a** to obtain 54.2 mg of **6a**, which consisted of white solid and m.p. 248.0–249.0 °C. **5a** was obtained in 40% overall yield (2 steps from **1**). The 1H-NMR (300 MHz, CDCl_3_): 6.77 (s, 1H, ArH-3), 7.34 (d, 2H, *J* = 8.6 Hz, ArH-2′, 6′), 7.46 (td, 1H, *J* = 7.6, 0.8 Hz, ArH-3″), 7.74 (td, 1H, *J* = 7.7, 1.4 Hz, ArH-6), 7.83–7.95 (m, 3H, ArH-7, 8, NH), 7.97–8.06 (m, 2H, ArH-3′, 5′), 8.14 (dd, 1H, *J* = 8.6, 0.8 Hz, ArH-5), 8.33–8.49 (m, 2H, ArH-4″, 6″), and 10.0 (s, 1H, CHO). The 13C-NMR (75 MHz, CDCl_3_): 98.6, 112.1, 117.5, 120.6, 121.7, 124.7, 127.2, 130.4, 131.3, 132.1, 133.5, 140.8, 151.8, 152.2, 161.7, and 190.5. Finally, the HRMS (+ESI) was C_22_H_15_N_4_O_2_ [M + H] +; it requires 367.1190, but has 367.1176. 

4-(2’,6’-Dimethyl-4’-formylphenoxy)-2-(5″-cyanopyridin-2″ylamino)quinoline **(6b**) With a 65% yield, the synthesis started with 50 mg (0.16 mmol) of **2b** to obtain 41.1 mg of **6b**, which consisted of white solid and m.p. 275.7–276.5 °C. **6b** was obtained in 45% overall yield (2 steps from **1**). The 1H-NMR (300 MHz, DMSO-d6): 2.20 (s, 6H, ArCH_3_-2′, 6′), 6.58 (s, 1H, ArH-3), 7.52 (t, 1H, *J* = 7 Hz, ArH-3″), 7.77 (td, 1H, *J* = 7.8, 16 Hz, ArH-6), 7.82–7.91 (m, 3H, ArH-7, 3′, 5′), 8.14 (dd, 1H, *J* = 8.8, 2.3 Hz, ArH-8), 8.29 (d, 1H, *J* = 7.5 Hz, ArH-5), 8.57 (d, 1H, *J* = 1.9 Hz, ArH-4″), 8.65 (d, 1H, *J* = 8.9 Hz, ArH-6″), 10.03 (s, 1H, CHO), and 10.37 (s, 1H, NH). The 13C-NMR (75 MHz, DMSO-d6): 15.6, 94.8, 97.5, 100.3, 111.8, 117.2, 117.9, 121.3, 124.2, 126.8, 130.7, 130.9, 131.9, 134.1, 141.1, 145.9, 151.9, 153.4, 154.5, 155.9, 159.7, and 160.4. Finally, the HRMS (+ESI) was C_24_H_19_N_4_O_2_ [M + H] +; it requires 395.1502, but has 395.1500. 

4-(4′-cyanophenoxy)- 2-(5″-cyanopyridin-2″ylamino)quinoline (**6c**) With a 60% yield, the synthesis started with 50 mg (0.18 mmol) of **2c** to obtain 38.8 mg of **5c**, which consisted of white solid and m.p. 225.7–225.9 °C. **6c** was obtained in 36% overall yield (2 steps from **1**). The 1H-NMR (300 MHz, CDCl_3_): 6.86 (s, 1H, ArH-3), 7.30 (d, 2H, *J* = 8.8 Hz, ArH-2′, 6′), 7.46 (td, 1H, *J* = 7.6 Hz, ArH-3″), 7.70–7.81 (m, 3H, ArH-6, 3′, 5′), 7.83–7.94 (m, 2H, ArH-7, 8), 8.11 (dd, 1H, *J* = 8.4, 0.7, ArH-5), 8.30 (bs, 2H, ArH-4″, NH), and 8.46 (d, 1H, *J* = 1.7 Hz, ArH-6″). The 13C-NMR (75 MHz, CDCl3): 98.8, 102.0, 112.3, 117.4, 118.1, 120.9, 121.7, 124.9, 131.5, 134.6, 140.8, 140.9, and 151.7. Finally, the HRMS (+ESI) was C_22_H_14_N_5_O [M + H] +; it requires 364.1193, but has 364.1193.

4-(2′,6′-Dimethyl-4′-cyanophenoxy)-2-(5’’-cyanopyridin-2’’ylamino)quinoline (**6d**) With a 72% yield, the synthesis started with 50 mg (0.16 mmol) of **2c** to obtain 45.6 mg of **5d**, which consisted of white solid and m.p. 229.4–230.3 °C. **5d** was obtained in 45% overall yield (2 steps from **1**). The 1H-NMR (300 MHz, CDCl_3_): 2.21 (s, 6H, ArCH_3_-2′, 6′), 6.12 (s, 1H, ArH-3), 7.43–7.57 (m, 3H, ArH-3″, 3′, 5′), 7.75 (td, 1H, *J* = 7.8, 1.4 Hz, ArH-6), 7.82–7.97 (m, 2H, ArH-7, 8), 8.23–8.44 (m, 3H, ArH-5, 4″, NH), and 8.58 (d, 1H, *J* = 8.3 Hz, ArH-6″). The 13C-NMR (75 MHz, CDCl3): 15.9, 93.8, 101.7, 110.0, 112.3, 117.5, 117.7, 118.2, 121.5, 124.5, 127.2, 131.1, 132.9, 133.1, 140.8, 148.3, 151.6, 152.5, 153.6, 155.7, and 160.6. Finally, the HRMS (+ESI) was C_24_H_18_N_5_O [M + H] +; it requires 392.1506, but has 392.1494. 

4,4′-di-(4′-formylphenoxy)-2,2′-biquinoline (**7a**) With the small amount during the synthesis process to produce compound **5a** and **6a**, which consisted of white solid and m.p. 288.7–289.3 °C. The 1H-NMR (300 MHz, CDCl_3_): 7.33–7.42 (m, 4H, 2 × ArH-2′, 6′), 7.59 (td, 2H, *J* = 7.7, 1.1 Hz, 2 × ArH-3), 7.76 (td, 2H, *J* = 7.7, 1.4 Hz, 2 × ArH-6), 7.98–8.06 (m, 4H, 2 × ArH-3′, 7′), 8.11 (d, 2H, *J* = 8.3 Hz, 2 × ArH-8), 8.21–8.30 (m, 4H, 2 × ArH-5, 5′), and 10.0 (s, 2H, 2 × CHO). The 13C-NMR (75 MHz, CDCl_3_): 105.1, 120.1, 121.7, 122.1, 127.2, 129.8, 130.4, 130.5, 132.2, 133.1, 149.6, 157.0, 160.4, 160.7, and 190.8. Finally, the HRMS (+ESI) was C_32_H_21_N_2_O_4_ [M + H] +; it requires 497.1496, but has 497.1491. 

4,4′-di-(2’,6’-Dimethyl-4’-formylphenoxy)-2,2′-biquinoline (**7b**) With the little amount during the synthesis process to produce compound **5b** and **6b**, which consisted of white solid and m.p. 300.0 °C (decomposed). The 1H-NMR (300 MHz, CDCl_3_ + CD_3_OD): 2.27 (s, 12H, 2 × ArCH3-2′, 6′), 7.55 (s, 2H, 2 × ArH-3), 7.59–7.69 (m, 2H, 2 × ArH-6), 7.71–7.86 (m, 6H, 2 × ArH-7, 3′, 5′), 8.04 (d, 2H, *J* = 8.3 Hz, 2 × ArH-8), 8.45 (d, 2H, *J* = 8.1 Hz, 2 × ArH-5), and 10.0 (s, 2H, 2 × CHO). The 13C-NMR (75 MHz, CDCl_3_ + CD_3_OD): 16.1, 100.0, 120.5, 121.4, 126.7, 129.3, 130.3, 130.9, 132.4, 133.9, 149.0, 155.3, 157.1, and 160.1. Finally, the HRMS (+ESI) was C_36_H_29_N_2_O_4_ [M + H] +; it requires 553.2122, but has 553.2116. 

2-(4′-formylphenoxy)-4-(4″-cyanophenyl)-aminoquinoline (**8a**) With a 58% yield, the synthesis started with 50 mg (0.18 mmol) of **3a** to obtain 37.4 mg of **8a**, which consisted of white solid and m.p. 238.5–238.9 °C. **8a** was obtained in a 6% overall yield (2 steps from **1**). The 1H-NMR (400 MHz, CDCl_3_ + CD_3_OD): 6.95 (s, 1H, ArH-3), 7.36–7.40 (m, 2H, ArH-2″, 6″), 7.41–7.45 (m, 3H, ArH-2′, 6′, NH), 7.50 (td, 1H, *J* = 6.0, 2.0 Hz, ArH-6), 7.64–7.71 (m, 3H, ArH-7, 3″, 5″), 7.79 (d, 1H, *J* = 8.0, 2.0 Hz, ArH-8), 7.95 (dt, 2H, *J* = 8.0, 2.0 Hz, ArH-3′, 5′), 8.13 (d, 1H, *J* = 8.0 Hz, ArH-5), and 9.97 (s, 1H, CHO). The 13C-NMR (75 MHz, CDCl_3_ + CD_3_OD): 95.0, 104.6, 113.8, 118.8, 119.2, 119.8, 120.2, 121.1, 124.6, 127.5, 130.3, 131.4, 132.1, 133.4, 145.3, 146.9, 149.5, 159.6, 161.3, and 191.4. Finally, the HRMS (+ESI) was C_23_H_16_N_3_O_2_ [M + H] +; it requires 366.1237, but has 366.1244. 

2-(2′,6′-Dimethyl-4′-formylphenoxy)-4-(4″-cyanophenyl)-aminoquinoline (**8b**) With a 66% yield, the synthesis started with 50 mg (0.16 mmol) of **3b** to obtain 41.6 mg of **8b**, which consisted of white solid and m.p. 200.0–201.3 °C. **8b** was obtained in a 9% overall yield (2 steps from **1**). The 1H-NMR (300 MHz, CDCl_3_): 2.21 (s, 6H, ArCH_3_-2′, 6′), 6.86 (s, 1H, NH), 6.98 (s, 1H, ArH-3), 7.35 (s, 1H, ArH-3′), 7.36 (s, 1H, ArH-5′) 7.42 (td, 1H, *J* = 7.6, 1.5 Hz, ArH-6′), 7.60 (td, 1H, *J* = 7.5, 1.2 Hz, ArH-7), 7.63–7.73 (m, 5H, ArH-8, 2″, 3″, 5″, 6″), 7.85 (d, 1H, *J* = 7.8 Hz, ArH-5), and 9.97 (s, 1H, CHO). The 13C-NMR (75 MHz, CDCl_3_): 16.8, 94.3, 105.9, 118.6, 118.9, 119.7, 119.8, 124.4, 128.9, 130.3, 132.6, 133.5, 133.9, 144.8, 147.7, 147.8, 155.7, 161.3, and 191.8. Finally, the HRMS (+ESI) was C_25_H_20_N_3_O_2_ [M + H] +; it requires 394.1550, but has 394.1542. 

2-(4′-cyanophenoxy)-4-(4″-cyanophenyl)-aminoquinoline (**8c**) With a 57% yield, the synthesis started with 50 mg (0.18 mmol) of **3c** to obtain 36.8 mg of **8c**, which consisted of white solid and m.p. 187.5–188.0 °C. **8c** was obtained in a 5% overall yield (2 steps from **1**). The 1H-NMR (400 MHz, CD_3_OD): 6.87 (s, 1H, ArH-3), 7.37 (d, 2H, *J* = 8.8 Hz, ArH-2″, 6″), 7.45–7.47 (m, 1H, ArH-6), 7.49 (d, 2H, *J* = 8.8 Hz, ArH-2′, 6′), 7.56–7.58 (m, 1H, ArH-7), 7.61–7.65 (m, 1H, ArH-8), 7.76 (d, 2H, *J* = 8.8 Hz, ArH-3″, 5″), 7.85 (d, 2H, *J* = 8.8 Hz, ArH-3′, 5′), 8.25 (d, 1H, *J* = 8.0 Hz, ArH-5), and 9.49 (s, 1H, NH). The 13C-NMR (100 MHz, DMSO-d6): 95.1, 104.4, 107.3, 119.2, 119.7, 119.7, 120.5, 122.6, 122.9, 124.9, 128.2, 131.0, 134.2, 134.5, 146.1, 147.2, 149.7, 158.0, and 161.9. Finally, the HRMS (+ESI) was C_23_H_15_N_4_O [M + H] +; it requires 363.1240, but has 363.1245. 

2-(2′,6′-Dimethyl-4′-cyanophenoxy)-4-(4″-cyanophenyl)-aminoquinoline (**8d**) With a 60% yield, the synthesis started with 50 mg (0.16 mmol) of **3d** to obtain 37.9 mg of **7d**, which consisted of white solid and m.p. 201.3–202.0 °C. **8d** was obtained in a 3% over-all yield (2 steps from **1**). The 1H-NMR (400 MHz, CDCl_3_): The 1H-NMR (400 MHz, CDCl_3_): 2.16 (s, 6H, ArCH3-2′, 6′), 6.98 (s, 1H, ArH-3), 7.02 (bs, 1H, NH), 7.38-7.44 (m, 5H, ArH-6, 2″, 3″, 5″, 6″), 7.58–7.65 (m, 2H, ArH-7, 8), 7.69 (d, 2H, *J* = 12 Hz, ArH-3′, 5′) and 7.89 (d, 1H, *J* = 8.0 Hz, ArH-5). The 13C-NMR (100 MHz, CDCl_3_): 16.0, 94.0, 105.8, 108.7, 114.4, 118.7, 118.9, 119.1, 119.8, 120.0, 124.5, 128.8, 130.3, 132.4, 133.1, 133.8, 133.8, 144.8, 147.5, 148.0, 154.3, and 161.1. Finally, the HRMS (+ESI) was C_25_H_19_N_4_O [M + H] +; it requires 391.1553, but has 391.1561. 

4-(4′-(2″-(E, Z)-cyanovinyl)-2′,6′-dimethyl-phenoxy)-2-(4″-cyanophenyl)-aminoquinoline (**9b**) With a 64% yield (E: Z isomer; 7: 3), the synthesis started with 100 mg (0.25 mmol) of **5b** to obtain 67.9 mg of **9b** which was obtained in a 30% overall yield (3 steps from **1**). In the case of Z-isomer; Z-isomer consisted of white solid and m.p. 217.3–217.9 °C. The 1H-NMR (300 MHz, CDCl_3_): 2.20 (s, 6H, ArCH_3_-2′, 6′), 5.47 (d, 1H, *J* = 12.1, Vinyl-H), 5.74 (s, 1H, ArH-3), 6.78 (bs, 1H, NH), 7.12 (d, 1H, *J* = 12.1 Hz, Vinyl-H), 7.45 (td, 1H, *J* = 7.4, 1.1 Hz, ArH-6), 7.52–7.66 (m, 4H, ArH-3′, 5′, 2″, 6″), 7.71 (td, 1H, *J* = 7.7, 1.4 Hz, ArH-7), 7.81–7.94 (m, 3H, ArH-8, 3″, 5″), and 8.29 (dd, 1H, *J* = 8.3, 1.0 Hz, ArH-5). The 13C-NMR (75 MHz, CDCl_3_): 16.1, 93.0, 95.2, 104.0, 117.3, 117.7, 118.2, 119.5, 121.6, 123.9, 127.2, 128.4, 130.1, 130.9, 131.5, 131.9,133.2, 144.6, 147.9, 148.6, 152.1, 153.5, and 161.1. Finally, the HRMS (+ESI) was C_27_H_21_N_4_O [M + H] + ; it requires 417.1710, but has 417.1712. In the case of E-isomer; E-isomer consisted of white solid and m.p. 214.4–215.1 °C. The 1H-NMR (300 MHz, CDCl_3_ + CD_3_OD): 2.20 (s, 6H, ArCH_3_-2′, 6′), 5.82 (s, 1H, ArH-3), 5.92 (d, 1H, *J* = 16.6 Hz, Vinyl-H), 7.30 (s, 2H, ArH-3′, 5′), 7.41 (d, 1H, *J* = 16.6 Hz, Vinyl-H), 7.40–7.48 (m, 1H, ArH-6), 7.56 (d, 2H, *J* = 8.7 Hz, ArH-2″, 6″), 7.66–7.76 (m, 1H, ArH-7), 7.84–7.94 (m, 3H, ArH-8, 3″, 5″), and 8.28 (dd, 1H, *J* = 8.1, 1.0 Hz, ArH-5). The 13C-NMR (75 MHz, CDCl3 + CD3OD): 15.8, 93.2, 95.9, 102.9, 117.48, 117.9, 119.6, 121.3, 123.5, 126.8, 128.2, 130.6, 131.2, 132.1, 133.0, 145.1, 148.4, 149.8, 152.5, 154.0, and 160.7. Finally, the HRMS (+ESI) was C_27_H_21_N_4_O [M + H] + ; it requires 417.1710, but has 417.1712. 

4-(4′-(2″-(E, Z)-cyanovinyl)-2′,6′-dimethyl-phenoxy)-2-(5″-cyanopyridin-2″ylamino)-aminoquinoline (**10b**) With a 60% yield (E: Z isomer; 7: 3), the synthesis started with 100 mg (0.25 mmol) of **6b** to obtain 63.5 mg of **10b** which was obtained in a 27% overall yield (3 steps from **1**). In the case of Z-isomer; Z-isomer consisted of white solid and m.p. 239.6–239.9 °C. The 1H-NMR (300 MHz, CDCl_3_): 2.20 (s, 6H, ArCH_3_-2′, 6′), 5.49 (d, 1H, *J* = 12.1 Hz, Vinyl-H), 6.11 (s, 1H, ArH-3), 7.13 (d, 1H, *J* = 12.1 Hz, Vinyl-H), 7.49 (t, 1H, *J* = 7.7 Hz, ArH-3″), 7.64 (s, 2H, ArH-3′, 5′), 7.74 (td, 1H, *J* = 7.6, 1.2 Hz, ArH-6), 7.81–7.95 (m, 3H, ArH-5, 7, 8), 8.07 (bs, 1H, NH), 8.27–8.43 (m, 3H, ArH-5, 4″), and 8.58 (d, 1H, *J* = 8.6 Hz, ArH-6″). The 13C-NMR (75 MHz, CDCl_3_): 16.1, 93.9, 95.2, 96.5, 112.2, 117.2, 117.6, 118.0, 121.7, 124.3, 127.2, 128.4, 130.1, 131.0, 131.6, 131.8, 140.7, 147.7, 148.3, 149.5, 151.6, 152.0, 152.5, 155.7, and 161.2. Finally, the HRMS (+ESI) was C_26_H_20_N_5_O [M + H] +; it requires 418.1662, but has 418.1657. In the case of E-isomer; E-isomer consisted of white solid and m.p. 236.6–237.4 °C. The 1H-NMR (300 MHz, CDCl_3_ + CD_3_OD): 2.21 (s, 6H, ArCH_3_-2′, 6′), 5.93 (d, 1H, *J* = 16.7 Hz, Vinyl-H), 6.28 (s, 1H, ArH-3), 7.32 (s, 2H, ArH-3′, 5′), 7.41 (d, 1H, *J* = 16.7 Hz, Vinyl-H), 7.46–7.55 (td, 1H, *J* = 7.6, 1.0 Hz, ArH-3″), 7.69–7.78 (m, 1H, ArH-6), 7.81–7.93 (m, 2H, ArH-7, 8), 8.28–8.39 (m, 2H, ArH-5, 4″), and 8.53 (s, 1H, ArH-6″). The 13C-NMR (75 MHz, CDCl_3_ + CD_3_OD): 15.8, 96.0, 100.9, 117.5, 117.9, 121.5, 124.2, 128.2, 129.1, 130.8, 131.2, 131.9, 140.5, 149.7, 151.3, 152.3, and 160.9. Finally, the HRMS (+ESI) was C_26_H_20_N_5_O [M + H] +; it requires 418.1662, but has 418.1657. 

2-(4′-(2″-(E, Z)-cyanovinyl)-2′,6′-dimethyl-phenoxy)-4-(4″-cyanophenyl)-aminoquinoline (**11b**) With a 56% yield, the synthesis started with 60 mg (0.15 mmol) of **8b** to obtain 35.6 mg of **11b**, which consisted of white solid and m.p. 180.7–182.3 °C. **10b** was obtained in a 5% overall yield (3 steps from **1**). The 1H-NMR (300 MHz, CDCl_3_, E, Z mixture): 2.16 (s, 6H, ArCH_3_-2′, 6′), 5.80 (d, 1H, *J* = 16.5, Vinyl-H), 6.89–6.99 (m, 1H, ArH-3), 7.20 (s, 1H, ArH-3′, 5′), 7.29–7.39 (m, 3H, Vinyl-H, ArH-2″, 6″), 7.54–7.63 (m, 2H, ArH-3″, 5″), 7.63–7.70 (m, 3H, ArH-6, 7, 8), and 7.86 (d, 1H, *J* = 8.3 Hz, ArH-5). The 13C-NMR (75 MHz, CDCl_3_, E, Z mixture): 16.8, 93.8, 94.2, 94.3, 95.1, 105.6, 105.7, 117.6, 118.5, 118.7, 119.0, 119.6, 119.7, 119.9, 124.3, 127.8, 128.8, 129.5, 130.3, 130.4, 130.6, 132.1, 132.3, 133.9, 144.9, 147.7, 148.3, 150.4, 152.6, 153.1, 161.5, and 161.6. Finally, the HRMS (+ESI) was C_27_H_21_N_4_O [M + H] +; it requires 417.1710, but has 417.1701. 

### 3.5. HIV-1 RT Inhibition Assay

The inhibition assay of HIV-1 RT was accomplished using the template/primer hybrid poly(A) × oligo(dT)15, digoxigenin (DIG)- and biotin-labeled nucleotides, an antibody to DIG that was conjugated to peroxidase (anti-DIG-POD), and the peroxidase substrate ABTS. The quantities of DIG- and biotin-labeled dUTP incorporated into DNA represent t HIV-1 RT activity. The HIV-RT inhibition assay was implemented using an RT assay kit (Roche Applied Science, Mannheim, Germany), and the procedures for assaying RT inhibition were performed as described in the kit protocol [34,35]. The tested compounds and three control drugs, namely, NVP, EFV, and RPV, were used at 1 µM concentration to determine the percentage inhibitory values. The reaction mixture consisted of template/primer complex, 2′-deoxy-nucleotide-5′-triphosphates (dNTPs), and RT enzyme in lysis buffer with or without an inhibitor. The reaction mixture was incubated for 1 h at 37 °C and then transferred to streptavidin-coated micro-titer plate (MTP). The biotin-labeled dNTPs that were incorporated in the template bound to streptavidin because of the presence of RT. Unbound dNTPs were washed using a wash buffer, and anti-DIG-POD was added to the MTP. The DIG-labeled dNTPs incorporated in the template bound to the anti-DIG-POD antibody. Unbound anti-DIG-POD was also washed elaborately five times using a wash buffer. Finally, the peroxide substrate (ABST) was added to the MTP. A colored reaction product emerged during the cleavage of the substrate catalyzed by peroxide enzyme. The absorbance of the sample was measured at the optical density (OD) at 490 nm using the MTP (enzyme-linked immunosorbent assay [ELISA]) reader. The percentage inhibitory activities of the RT inhibitors were calculated by comparing with that of the sample without an inhibitor. The resulting color intensity was directly proportional to the RT activity. Percentage inhibitory values were calculated using the following formula: Inhibition (%) = [1–(OD value with RT and inhibitor−OD value without RT and inhibitors)/(OD value without inhibitors with RT−OD value without RT and inhibitors)] × 100. Half maximal inhibitory concentration (IC50) was determined as the concentrations of the compounds of interest and the three control drugs at 50% cell growth inhibition using the plotting of the sigmoid curve between the log of the concentration on the x axis and the inhibition rate on the y axis. 

### 3.6. Cytotoxic Activity

The cell lines were seeded in a 96-well microplate (Costar No. 3599) at a density of 5 × 10^3^ -2 × 10^4^ cells/well (100 μL/well). Background control wells contained the same volume as the complete culture medium. The microplate was incubated for 24 h at 37 °C with 5% CO_2_ and 95% humidity (Shellab). Samples at various concentrations were added to the microplate and incubated for another 48 h. Cell viability was determined by 3(4,5-dimethylthiazol-2-yl)-2-5-diphenyl tetrazolium bromide (MTT) assay (Sig-ma-Aldrich) [36,37]. The reagent was dissolved in phosphate-buffered saline at 5 mg/mL and filtered to sterilize and remove the small amount of insoluble residue present in some batches of MTT. MTT solution (10 µL/100 µL medium) was added to all wells of each assay, and the plates were incubated at 37 °C with 5% CO_2_ and 95% humidity for 2–4 h. Subsequently, dimethyl sulfoxide (100 µL; Merck, Germany) was added to dissolve the resulting formazan by sonication. The plates were read on a microplate reader (Molecular Devices, CA, USA) using a test wavelength of 550 nm and a reference wavelength of 650 nm. XTT assay for suspension cells was used for MOLT-3 cells [36]. The plates were incubated for 4 h after the addition of a 50-µL mixture of 1 mg/mL (5 mL) and 0.383 mg/mL (100 µL) phenazine methosulfate. The absorbance of the orange formazan compounds was measured at the wavelengths of 492 and 690 nm. IC50 values were determined as the drug and sample concentrations at 50% cell growth inhibition. 

## 4. Conclusions

2-Phenylamino-4-phenoxyquinoline derivatives were developed using the molecular hybridization approach from currently available drugs, namely, NVP, EFV, and RPV, to generate a novel series of quinoline derivatives, explore the chemical structures of a range of NNRTIs, and overcome the issue of resistance. The substituents at the 2- and 4-positions of quinoline as the core structure are important. This exploratory study clearly highlights that 2′,6′-dimethyl-4′-cyanophenoxy and 2′,6′-dimethyl-4′-formylphenoxy at the 4-position and 5″-cyanopyridin-2″ylamino at the 2-position of quinoline affect its inhibitory activity against HIV-1 RT. 4-(2′,6′-Dimethyl-4′-formylphenoxy)-2-(5″-cyanopyridin-2″ylamino)quinoline (**6b**) and 4-(2′,6′-dimethyl-4′-cyanophenoxy)-2-(5″-cyanopyridin-2″ylamino)quinoline (**6d**) demonstrated activities against HIV-1 RT with IC50 values of 1.93 and 1.22 µM, respectively, similar to that of NVP (IC50 = 1.05 µM). Furthermore, all of the synthesized compounds exhibited activities against HIV-1 RT and cytotoxicity to cancer cell lines. Compound **6b** showed a strong activity against MOLT-3, HeLA, and HL-60 cells with IC50 values of 12.7 ± 1.1, 25.7 ± 0.8, and 20.5 ± 2.1 µM, respectively, similar to those of EFV and RPV. Compound **6d** had high inhibitory activity with a very low IC50 value against HIV-1 RT and showed no cytotoxicity toward normal embryonic lung (MRC-5) cells compared with EFV and RPV. Therefore, 2-phenylamino-4-phenoxyquinoline derivatives can be further improved for the development of HIV-1 RT inhibitors as a potential treatment for HIV and cancer in one drug. 

## Figures and Tables

**Table 1:** Binding energy and interaction of the derivatives and commercially available drugs with HIV-1 RT based on the molecular docking analysis.

Ligands	Binding Energy (kcal/mol) ^a^	NOC ^b^	Interaction Amino Acids 3 Å (4G1Q)	
			H-Bond	Pi–Pi Stacking
4a	−11.5 ± 0	83 ± 5	HIS235	-
4b	−12.6 ± 0	21 ± 1	-	TYR188 TRP229
4c	−12.3 ± 0	137 ± 3	LYS101	TRP229 TYR318
4d	−13.2 ± 0	62 ± 6	TYR188 TRP229	TYR188 TRP229
5a	−12.1 ± 0	64 ± 7	LYS101 HIS235	TYR318
5b	−13.2 ± 0	81 ± 9	LYS101 HIS235	TYR181 TYR318
5c	−12.5 ± 0	87 ± 7	LYS101 HIS235	TRP229 TYR318
5d	−13.7 ± 0	96 ± 5	LYS101 HIS235	TYR318
6a	−13.7 ± 0	68 ± 6	LYS101 HIS235	TYR188 TYR318
6b	−12.7 ± 0	70 ± 75	LYS101 HIS235 PRO236	TYR188 TRP229 TYR318
6c	−12.0 ± 0	61 ± 4	LYS101 HIS235	TYR188 TYR318
6d	−13.1 ± 0	74 ± 1	LYS101 HIS235 PRO236	TYR188 TRP229 TYR318
7a	−11.7 ± 0	59 ± 3	TYR318	TYR181
7b	−12.1 ± 0	12 ± 2	TYR318	TYR181
8a	−12.0 ± 0	76 ± 5	HIS235	TYR181 TRP229 TYR318
8b	−12.7 ± 0	76 ± 10	LYS101 HIS235 PRO236	TYR181 TYR188 TRP229 TYR318
8c	−12.3 ± 0	125 ± 6	LYS101 HIS235	TYR181 TRP229 TYR318
8d	−13.2 ± 0	84 ± 6	LYS101 HIS235 PRO236	TYR181 TYR188 TRP229 TYR318
9b-trans	−14.8 ± 0	110 ± 6	LYS101	TRP229
9b-cis	−14.0 ± 0	47 ± 2	LYS101 HIS235	TYR318
10b-trans	−14.3 ± 0	90 ± 5	LYS101 HIS235 PRO236	TYR181 TRP229 TYR318
10b-cis	−13.5 ± 0	45 ± 6	LYS101 HIS235	TYR181 TYR318
11b	−14.2 ± 0 (T)	95 ± 7	LYS101 HIS235	TYR181 TYR188 TRP229
	−13.8 ± 0 (C)	45 ± 4	LYS101 PRO236	TYR181 TRP229
NVP	−7.9 ± 0	122 ± 0	LYS101	-
EVF	−9.2 ± 0	132 ± 4	LYS101	-
RVP	−12.5 ± 0	129 ± 9	LYS101	TYR181 TRP229

^a^ Results are expressed as the mean ± standard error of binding energy (kcal/mol). ^b^ Results are expressed as the mean ± standard error of the number of ligand conformations.

**Table 2:** Molecular properties of the derivatives and commercially available drugs calculated by SwissADME software.

Ligands	Number of H-Bond Acceptors	Number of H-Bond Donors	LogP	Number of Rotatable Bonds	Molecular Weight (g/mol)	TPSA (Å^2^)
4a	5	0	2.75	6	369	65.49
4b	5	0	3.58	6	425	65.49
4c	5	0	2.75	4	363	78.93
4d	5	0	3.58	4	419	78.93
5a	5	1	2.75	4	365	75.01
5b	5	1	3.17	4	393	75.01
5c	4	1	2.75	4	362	81.73
5d	4	1	3.17	4	390	81.73
6a	5	1	1.73	5	366	87.90
6b	5	1	2.15	5	394	87.90
6c	5	1	1.73	4	363	94.62
6d	5	1	2.15	4	391	94.62
7a	5	0	3.92	6	468	61.31
7b	5	0	4.65	6	524	61.31
8a	4	1	2.75	5	365	75.01
8b	4	1	3.17	5	393	75.01
8c	4	1	2.75	4	362	81.73
8d	4	1	3.17	4	390	81.73
9 b-trans	4	1	3.51	5	416	81.73
9 b-cis	4	1	3.51	5	416	81.73
10 b-trans	5	1	2.50	5	417	94.62
10 b-cis	5	1	2.50	5	417	94.62
11 b-trans	4	1	3.51	4	416	81.73
11 b-cis	4	1	3.51	4	416	81.73
NVP	1	1	2.14	1	266	63.57
EVF	1	1	3.61	1	315	38.33
RPV	5	3	2.37	5	366	97.42

Lipinski’s rule of five states that molecular weight must be less than 500 Da, lipophilicity (logP) must be less than 5, H-bond donors must be less than 5, H-bond accepter must be less than 10. Other parameters were considered. For example, total polar surface area (TPSA) should be less than 140 Å, and the number of rotatable bonds should be less than 10.

**Table 3:** Inhibitory analysis of **6b**, **6d**, NVP, EFV, and RPV against HIV-1 RT ^a^.

Compounds	IC50 (µM) ^b^
**6b**	1.93
**6d**	1.22
NVP	1.05
EFV	0.06
RPV	0.06

^a^ The RT kit was commercially available and supplied by Roche, and the data were obtained by standard ELISA. ^b^ Results are expressed as the mean ± standard error of inhibitory activity (%).

**Table 4:** In vitro cytotoxicity of the derivatives and commercially available drugs to human cancer cell lines and normal cell line.

Compounds (Molecular Weight)	Cell Lines ^a^ (IC50 [µM]) ^b^				
	HepG2 ^a^	MOLT-3 ^a^	HuCCA-1 ^a^	A549 ^a^	MRC-5 ^a^
4a (369.4)	90.9 ± 2.1	25.1 ± 2.6	48.1 ± 1.7	%C = 40.0 ^c^	84.3 ± 9.6
4b (425.5)	50.6 ± 1.2	61.6 ± 0.7	11.1 ± 0.7	38.5 ± 1.3	60.4 ± 2.0
4c (363.4)	%C = 41.4 ^c^	%C = 11.0 ^c^	%C = 15.0 ^c^	%C = 12.0 ^c^	107.6 ± 6.4
4d (419.5)	92.2 ± 6.8	%C = 13.0 ^c^	83.5 ± 11.1	55.5 ± 5.7	%C = 26.9 ^c^
5a (365.4)	73.2 ± 7.7	15.6 ± 3.6	29.7 ± 2.0	121.7 ± 4.5	107.9 ± 4.7
5b (393.4)	65.9 ± 6.1	11.8 ± 1.6	29.4 ± 2.0	51.2 ± 3.0	69.8 ± 4.3
5c (362.4)	%C = 44.8 ^c^	%C = 6.0 ^c^	%C = 23.0 ^c^	%C = 26.0 ^c^	%C = 12.0 ^c^
5d (390.4)	%C = 38.2 ^c^	48.8 ± 41.0	%C = 3.0 ^c^	%C = 21.0 ^c^	%C = 7.4 ^c^
6a (366.4)	23.7 ± 1.0	5.13 ± 1.5	14.9 ± 1.1	%C = 42.0 ^c^	18.3 ± 3.9
6b (393.4)	92.8 ± 11.8	12.7 ± 1.1	125.8 ± 1.8	107.9 ± 1.2	%C = 26.1 ^c^
6c (363.4)	101.7 ± 0.7	14.1 ± 0.4	22.9 ± 0.9	14.0 ± 2.2	69.2 ± 12.4
6d (391.4)	%C = 0.0 ^c^	%C = 27.0 ^c^	%C = 0.0 ^c^	%C = 8.0 ^c^	%C = 0.0 ^c^
7a (496.5)	69.8 ± 11.8	74.2 ± 6.3	88.2 ± 4.4	69.5 ± 3.3	%C = 33.9 ^c^
7b (552.6)	%C = 0.1 ^c^	%C = 6.0 ^c^	%C = 4.0 ^c^	%C = 15.0 ^c^	%C = 4.2 ^c^
8a (365.4)	85.3 ± 3.5	21.5 ± 3.3	22.7 ± 1.9	111.2 ± 5.6	119.0 ± 5.7
8b (393.4)	105.8 ± 9.7	22.3 ± 1.9	%C = 43.0 ^c^	%C = 37.0 ^c^	%C = 9.7 ^c^
8c (393.4)	%C = 40.3 ^c^	%C = 2.1 ^c^	%C = 25.8 ^c^	%C = 29.0 ^c^	%C = 17.2 ^c^
8d (393.4)	%C = 32.0 ^c^	67.4 ± 3.1	%C = 18.4 ^c^	%C = 29.1 ^c^	%C = 15.5 ^c^
9b-trans (416.5)	%C = 42.9 ^c^	%C = 19.0 ^c^	%C = 8.0 ^c^	%C = 8.0 ^c^	%C = 28.4 ^c^
9b-cis (416.5)	%C = 9.8 ^c^	52.8 ± 2.1	%C = 10.0	%C = 1.0 ^c^	%C = 0.0 ^c^
10b-trans (417.5)	%C = 1.0 ^c^	%C = 2.0 ^c^	%C = 0.0 ^c^	%C = 1.0 ^c^	%C = 0.0 ^c^
10b-cis (417.5)	%C = 0.0 ^c^	%C = 11.0 ^c^	%C = 0.0 ^c^	%C = 0.0 ^c^	%C = 0.0 ^c^
11b (416.5)	%C = 3.7 ^c^	%C = 19.0 ^c^	%C = 0.0 ^c^	%C = 0.0 ^c^	%C = 0.0 ^c^
NVP (266.3)	%C = 15.4 ^c^	%C = 29.0 ^c^	%C = 11.0 ^c^	%C = 18.0 ^c^	%C = 1.0 ^c^
EFV (315.7)	86.6 ± 3.9	24.6 ± 1.2	59.6 ± 2.4	60.2 ± 1.8	95.6 ± 6.0
RPV (366.4)	87.4 ± 0.1	4.3 ± 0.5	61.3 ± 1.7	%C = 42.0 ^c^	%C = 14.4 ^c^
Doxorubicin (543.5)	0.6 ± 0.0	0.02 ± 0.00	0.9 ± 0.0	0.6 ± 0.0	3.1 ± 0.5
Etoposide (588.6)	49.9 ± 1.1	0.03 ± 0.0	-	-	-

^a^ HepG2 (hepatocarcinoma), MOLT-3 (acute lymphoblastic leukemia), HuCCA-1 (cholangiocarcinoma), A549 (lung carcinoma), MRC-5 (normal embryonic lung cell). ^b^ Results are expressed as the mean ± standard error of inhibition perceptual for all cell lines. Doxorubicin and etoposide were used as the positive control. Experiments were performed in triplicate. ^c^ Inactive (IC_50_ ≥ 50 µg/mL); reported in percentage cytotoxicity at the substance concentration of 50 µg/mL.

**Table 5:** In vitro cytotoxicity of the derivatives and commercially available drugs to human cancer cell lines.

Compounds (Molecular Weight)	Cell Lines ^a^ (IC50 [µM]) ^b^					
	MDA-MB-231 ^a^	S102 ^a^	HeLA ^a^	T47-D ^a^	H69AR ^a^	HL-60 ^a^
4a (369.4)	%C = 43.3 ^c^	%C = 0.4 ^c^	103.5 ± 7.9	%C = 34.0 ^c^	%C = 0.0 ^c^	92.4 ± 3.1
4b (425.5)	15.3 ± 1.6	89.4 ± 0.1	5.3 ± 0.2	22.5 ± 3.3	30.9 ± 5.7	5.7 ± 2.0
4c (363.4)	92.5 ± 4.2	%C = 23.7 ^c^	%C = 23.0 ^c^	%C = 27.0 ^c^	%C = 21.0 ^c^	%C = 15.0 ^c^
4d (419.5)	92.5 ± 5.0	%C = 31.5 ^c^	%C = 40.0 ^c^	55.2 ± 1.5	%C = 38.0 ^c^	%C = 23.0 ^c^
5a (365.4)	77.7 ± 5.9	%C = 20.9 ^c^	12.6 ± 1.3	43.0 ± 5.3	%C = 38.0 ^c^	16.6 ± 4.7
5b (393.4)	15.9 ± 0.4	74.0 ± 5.3	3.9 ± 0.6	22.9 ± 0.9	27.6 ± 3.4	4.5 ± 0.2
5c (362.4)	%C = 38.4 ^c^	%C = 0.0 ^c^	%C = 25.0 ^c^	%C = 45.0 ^c^	%C = 26.0 ^c^	%C = 2.0 ^c^
5d (390.4)	%C = 14.9 ^c^	%C = 0.0 ^c^	83.4 ± 5.7	%C = 24.0 ^c^	%C = 0.0 ^c^	33.4 ± 1.7
6a (366.4)	68.0 ± 2.0	%C = 31.7 ^c^	15.4 ± 0.8	46.3 ± 0.2	64.2 ± 2.5	15.0 ± 1.8
6b (393.4)	102.7 ± 1.9	111.5 ± 1.8	25.7 ± 0.8	%C = 38.0 ^c^	%C = 21.0 ^c^	20.5 ± 2.1
6c (363.4)	66.6 ± 3.0	%C = 44.8 ^c^	67.7 ± 0.6	47.1 ± 3.3	66.2 ± 2.4	%C = 9.0 ^c^
6d (391.4)	%C = 8.3 ^c^	%C = 0.0 ^c^	%C = 0.0 ^c^	%C = 1.0 ^c^	%C = 0.0 ^c^	%C = 13.0 ^c^
7a (496.5)	74.1 ± 5.3	%C = 17.6 ^c^	88.0 ± 4.3	96.1 ± 3.3	%C = 20.0 ^c^	%C = 25.0 ^c^
7b (552.6)	%C = 4.6 ^c^	%C = 0.0 ^c^	%C = 13.0 ^c^	%C = 36.0 ^c^	%C = 9.0 ^c^	%C = 15.0 ^c^
8a (365.4)	117.7 ± 5.8	%C = 2.9 ^c^	112.3 ± 3.1	%C = 0.0 ^c^	%C = 8.4 ^c^	106.9 ± 1.4
8b (393.4)	107.0 ± 3.2	%C = 0.0 ^c^	42.3 ± 2.7	%C = 38.0 ^c^	%C = 6.0 ^c^	34.9 ± 5.1
8c (393.4)	%C = 0.0 ^c^	%C = 0.0 ^c^	%C = 0.0 ^c^	%C = 35.0 ^c^	%C = 21.0 ^c^	%C = 1.0 ^c^
8d (393.4)	%C = 32.4 ^c^	%C = 0.0 ^c^	103.8 ± 4.7	%C = 31.0 ^c^	%C = 0.0 ^c^	56.6 ± 4.7
9b-trans (416.5)	%C = 24.9 ^c^	%C = 0.0 ^c^	%C = 38.0 ^c^	%C = 0.0 ^c^	%C = 14.0 ^c^	%C = 17.0 ^c^
9b-cis (416.5)	%C = 10.0 ^c^	%C = 0.0 ^c^	97.2 ± 5.1	%C = 5.0 ^c^	%C = 0.0 ^c^	%C = 42.0 ^c^
10b-trans (417.5)	%C = 17.0 ^c^	%C = 3.1 ^c^	%C = 10.0 ^c^	%C = 0.0 ^c^	%C = 0.0 ^c^	%C = 31.0 ^c^
10b-cis (417.5)	%C = 0.0 ^c^	%C = 0.0 ^c^	%C = 0.0 ^c^	%C = 0.0 ^c^	%C = 0.0 ^c^	%C = 5.0 ^c^
11b (416.5)	%C = 14.1 ^c^	%C = 0.0 ^c^	%C = 2.0 ^c^	%C = 4.0 ^c^	%C = 3.0 ^c^	%C = 0.0 ^c^
NVP (266.3)	%C = 8.9 ^c^	%C = 3.0 ^c^	%C = 34.0 ^c^	%C = 14.0 ^c^	%C = 8.0 ^c^	%C = 3.0 ^c^
EFV (315.7)	77.7 ± 5.6	93.5 ± 4.7	46.2 ± 1.3	59.2 ± 0.8	105.0 ± 3.4	33.7 ± 1.1
RPV (366.4)	25.3 ± 2.9	22.5 ± 1.2	11.3 ± 1.2	15.0 ± 1.3	57.1 ± 2.0	11.5 ± 0.8
Doxorubicin (543.5)	2.5 ± 0.1	2.2 ± 0.0	0.4 ± 0.0	1.2 ± 0.0	34.3 ± 0.1	0.1 ± 0.0
Etoposide (588.6)	-	-	-	-	-	0.8 ± 0.1

^a^ MDA-MB-231 (hormone-independent breast cancer), S102 (Thai liver cancer), HeLA (cervical carcinoma), T47-D (hormone-dependent breast cancer), H69AR (lung cancer, multidrug resistant), HL-60 (promyeloblast). ^b^ Results are expressed as the mean ± standard error of inhibition perceptual for all cell lines. Doxorubicin and etoposide were used as the positive control. Experiments were performed in triplicate. ^c^ Inactive (IC_50_ ≥ 50 µg/mL); reported in percentage cytotoxicity at the substance concentration of 50 µg/mL.

## Data Availability

Not applicable.

## Supplementary Materials

The following are available online, ^1^ H and ^13^ C NMR for all synthesized compounds were revealed in the supplementary materials.

## Sample Availability

The samples of all compounds are available from authors.
