# Supplementary material for: Structural Basis of 2-Phenylamino-4-phenoxyquinoline Derivatives as Potent HIV-1 Non-Nucleoside Reverse Transcriptase Inhibitors
Source: Molecules. 2022 Jan 11;27(2):461. doi: 10.3390/molecules27020461 (PMC8781960; doi:10.3390/molecules27020461)
Supplement: Supplementary file 1 [file molecules-27-00461-s001.zip › molecules-1497757-supplementary.pdf]

Supplementary Materials

Structural basis of 2-phenylamino-4-phenoxyquinoline derivatives as potent HIV-1 non-nucleoside reverse transcriptase inhibitors

Arthit Makarasen[1], Suwicha Patnin[1], Pongsit Vijitphan[1], Nanthawan Reukngam[1], Panita Khlaychan[1], Mayuso Kuno[3], Pakamas Intachote[2], Busakorn Saimanee[2], Suchada Sengsai[2], Supanna Techasakul[1]*

[1] Department of Chemistry, Laboratory of Organic Synthesis, Chulabhorn Research Institute, Laksi, Bangkok 10210, Thailand; suwicha@cri.or.th (S.P.); pongsit@cri.or.th (P.V.); nanthawan@cri.or.th (N.R.); panita@cri.or.th (P.K.);

[2] Biological Activity Test and Screening Unit, Central Facilities, Chulabhorn Research Institute, Laksi, Bangkok 10210, Thailand; pakamas@cri.or.th (P.I.); busakorn@cri.or.th (B.S.); suchada@cri.or.th (S.S.)

[3] Department of Chemistry, Faculty of Science, Srinakharinwirot University, Wattana, Bangkok 10110, Thailand; apinyac@g.swu.ac.th (A.C.); mayuso@g.swu.ac.th (M.K.)

* Correspondence: supanna@cri.or.th (S.T.)

General procedure for the preparation of 2-chloro-4-phenoxyquinoline (2a–2d). A mixture of 2,4-dichloroquinoline (**1**, 10 mmol) and hydroxyl benzene (11 mmol) in dimethylformamide (DMF, 30 mL) with anhydrous cesium carbonate (Cs_2CO_3, 20 mmol) was heated in a sealed tube, stirred at 80 °C for 16 h, and cooled. Then, the mixture was poured into ice-water and extracted thrice with ethyl acetate. The combined organic layers were washed with saturated NaCl and dried over Na_2SO_4. The crude product was purified on a silica gel column (eluent: hexane/ethyl acetate) to obtain **2a**–**2d** with 62%–69% yield. However, 4-chloro-2-phenoxyquinoline (**3a**–**3d**) was also found during the reaction progress as a side product with 5%–14% yield.

4-(4′-formylphenoxy)-2-chloroquinoline (2) With 67% yield, the synthesis started with 0.30 g (1.51 mmol) of **1** to obtain 0.29 g of **2a**, which consisted of white solid and mp. 113.9 °C -114.2 °C. The ^{1}H-NMR (300 MHz, $CDCl_3$): 6.64 (s, 1H, ArH-3), 7.35 (d, 2H, J = 8.7 Hz, ArH-2′, 6′), 7.61 (td, 1H, J = 7.8, 1.2 Hz, ArH-6), 7.81 (td, 1H, J = 7.8, 1.2 Hz, ArH-7), 8.04 (m, 3H, ArH-8, 3′, 5′), and 8.26 (dd, 1H, J = 8.4, 0.9 Hz, ArH-5), and 10.1 (s, 1H, CHO). The ^{13}C-NMR (75 MHz, $CDCl_3$): 106.5, 120.4, 121.0, 121.8, 126.9, 128.5, 131.6, 132.3, 134.0, 149.0, 151.0, 159.1, 161.9, and 190.5. Finally, the HRMS (+ESI) was $C_{16}H_{11}ClNO_2$ $[M+H]^+$; it requires 284.0478, but has 284.0465. **2-(4′-formylphenoxy)-4-chloroquinoline (3a)** obtained as the side product with 11 % yield (47.2 mg), which consisted of white solid and mp. 121.7 °C -122.5 °C. The ^{1}H-NMR (300 MHz, $CDCl_3$): 7.26 (s, 1H, ArH-3), 7.41 (dd, 2H, J = 8.6, 1.8 Hz, ArH-2′, 6′), 7.53 (td, 1H, J = 7.6, 1.2 Hz, ArH-6), 7.68 (td, 1H, J = 7.7, 1.4 Hz, ArH-7), 7.79 (dd, 1H, J = 8.4, 0.5 Hz, ArH-8), 7.95 (dt, 2H, J = 8.6, 2.4 Hz, ArH-3′, 5′), 8.16 (dd, 1H, J = 8.3, 1.0 Hz, ArH-5), and 10.0 (s, 1H, CHO). The ^{13}C-NMR (75 MHz, $CDCl_3$): 112.9, 121.6, 124.0, 124.2, 126.1, 128.2, 131.0, 131.4, 133.1, 145.2, 146.4, 158.5, 159.9 and 190.9. Finally, the HRMS (+ESI) was $C_{16}H_{11}ClNO_2$ $[M+H]^+$; it requires 284.0478, but has 284.0468.

4-(2′,6′-Dimethyl-4′-formylphenoxy)-2-chloroquinoline (2b) With 69% yield, the synthesis started with 1.0 g (5.05 mmol) of **1** to obtain 1.09 g of **2b**, which consisted of white solid and mp. 154.6 °C -156.9 °C. The ^{1}H-NMR (300 MHz, $CDCl_3$): 2.23 (s, 6H, $ArCH_3$-2′, 6′), 6.20 (s, 1H, ArH-3), 7.65 (td, 1H, J = 7.5, 1.2 Hz, ArH-6), 7.75 (s, 2H, ArH-3′, 5′), 7.82 (td, 1H, 7.5, 1.2 Hz, ArH-7), 8.04 (dd, 1H, 8.3, 0.9 Hz, ArH-8), and 8.4 (dd, 1 H, 8.3, 0.9 Hz, ArH-5), and 10.0 (s, 1H, CHO). The ^{13}C-NMR (75 MHz, $CDCl_3$): 16.1, 102.9, 119.5, 121.7, 126.7, 128.5, 131.5, 132.1, 134.4, 148.8, 151.3, 154.6, 161.0, and 191.2. Finally, the HRMS (+ESI) was $C_{18}H_{15}ClNO_2$ $[M+H]^+$; it requires 312.0791, but has 312.0791. **2-(2′,6′-Dimethyl-4′-formylphenoxy)-4-chloroquinoline (3b)** obtained as the side product with 14 % yield (0.22 g), which consisted of white solid and mp. 113.7 °C -114.2 °C. The ^{1}H-NMR (300 MHz, $CDCl_3$): 2.21 (s, 6H, $ArCH_3$-2′, 6′), 7.27 (d, 1H, J = 5.8 Hz, ArH-3), 7.50

(td, 1H, J = 7.4, 1.7 Hz, ArH-6), 7.60-7.70 (m, 4H, ArH-7, 8, 3′, 5′), 8.15 (dd, 1H, J = 8.5, 0.8 Hz, ArH-5), and 9.99 (s, 1H, CHO). The ^{13}C-NMR (75 MHz, $CDCl_3$): 16.7, 111.5, 123.8, 124.0, 125.6, 128.2, 130.3, 130.9, 132.5, 133.7, 145.2, 146.9, 155.3, 159.7 and 191.7. Finally, the HRMS (+ESI) was $C_{18}H_{15}ClNO_2$ $[M+H]^+$; it requires 312.0791, but has 312.0777.

4-(4′-cyanophenoxy)-2-chloroquinoline (2c) With 60% yield, the synthesis started with 0.30 g (1.51 mmol) of **1** to obtain 0.26 g of **2c**, which consisted of white solid and mp. 214.1 °C -215.0 °C. The ^{1}H-NMR (300 MHz, $CDCl_3$): 6.63 (s, 1H, ArH-3), 7.32 (d, 2H, J = 8.7 Hz, ArH-2′, 6′), 7.60 (t, 1H, J = 7.2 Hz, ArH-6), 7.78-7.83 (m, 3H, ArH-7, 3′, 5′), 8.02 (d, 1H, J = 8.4 Hz, ArH-8), and 8.22 (d, 1H, J = 8.4 Hz, ArH-5). The ^{13}C-NMR (75 MHz, $CDCl_3$): 106.4, 109.6, 117.9, 119.6, 120.2, 121.2, 121.6, 126.9, 128.4, 131.5, 134.4, 134.7, 148.8, 150.8, 157.6, 161.5. Finally, the HRMS (+ESI) was $C_{16}H_{10}ClN_2O$ $[M+H]^+$; it requires 281.0482, but has 281.0470. **2-(4′-cyanophenoxy)-4-chloroquinoline (3c)** obtained as the side product with 9 % yield (38.3 mg), which consisted of white solid and mp. 147.0 °C -148.0 °C. The ^{1}H-NMR (300 MHz, $CDCl_3$): 7.27 (d, 1H, J = 5.7 Hz, ArH-3), 7.39 (dt, 2H, J = 8.4, 0.8 Hz, ArH-2′, 6′), 7.56 (td, 1H, J = 7.3, 1.3 Hz, ArH-6), 7.65-7.82 (m, 4H, ArH-7, 8, 3′, 5′), and 8.18 (dd, 1H, J = 8.1, 0.9 Hz, ArH-5). The ^{13}C-NMR (75 MHz, $CDCl_3$): 108.4, 112.8, 118.6, 122.1, 124.1, 124.3, 126.3, 128.2, 131.1, 133.8, 145.4, 146.4, 156.9 and 159.7. Finally, the HRMS (+ESI) was $C_{16}H_{10}ClN_2O$ $[M+H]^+$; it requires 281.0482, but has 281.0475.

4-(2′,6′-Dimethyl-4′-cyanophenoxy)-2-chloroquinoline (2d) With 62% yield, the synthesis started with 0.30 g (1.51 mmol) of **1** to obtain 0.29 g of **2d**, which consisted of white solid and mp. 184.3 °C -185.0 °C. The ^{1}H-NMR (300 MHz, $CDCl_3$): 2.20 (s, 6H, $ArCH_3$-2′, 6′), 6.20 (s, 1H, ArH-3), 7.53 (s, 2H, ArH-3′, 5′), 7.65 (td, 1H, J = 9.2, 1.5 Hz, ArH-6), 7.83 (td, 1H, J = 9.2, 1.5 Hz, ArH-7), 8.04 (dd, 1H, J = 8.3, 1.2 Hz, ArH-8), and 8.38 (dd, 1H, J = 8.3, 1.2 Hz, ArH-5). The ^{13}C-NMR (75 MHz, $CDCl_3$): 15.9, 102.7, 110.4, 118.1, 119.3, 121.6, 126.8, 128.4, 131.5, 132.7, 133.3, 148.8, 151.1, 153.2, and 160.7. Finally, the HRMS (+ESI) was $C_{18}H_{14}ClN_2O$ $[M+H]^+$; it requires 309.0795, but has 309.0783. **2-(2′,6′-Dimethyl-4′-cyanophenoxy)-4-chloroquinoline (3d)** obtained as the side product with 5 % yield (23.4 mg), which consisted of white solid and mp. 152.2 °C -153.0 °C. The ^{1}H-NMR (300 MHz, $CDCl_3$): 2.16 (s, 6H, $ArCH_3$-2′, 6′), 7.29 (s, 1H, ArH-3), 7.44 (s, 2H, ArH-3′, 5′), 7.51 (td, 1H, J = 7.3, 2.2 Hz, ArH-6), 7.60-7.69 (m, 2H, ArH-7, 8) and 8.16 (d, 1H, J = 8.2 Hz, ArH-5). The ^{13}C-NMR (75 MHz, $CDCl_3$): 16.6, 109.1, 111.5, 118.9, 123.9, 124.0, 125.7, 128.1, 130.9, 132.5, 133.0, 145.3, 146.7, 153.9 and 159.4. Finally, the HRMS (+ESI) was $C_{18}H_{14}ClN_2O$ $[M+H]^+$; it requires 309.0795, but has 309.0787.

General procedure for the preparation of 2,4-diphenoxyquinoline (4a–4d). A mixture of **1** (10 mmol) and hydroxyl benzene (21 mmol) in DMF (30 mL) with anhydrous Cs_2CO_3

(20 mmol) was heated in a sealed tube, stirred at 120 °C for 8–16 h, and cooled. Afterward, the mixture was poured into ice-water and extracted thrice with ethyl acetate. The combined organic layers were washed with saturated NaCl and dried over Na_2SO_4. The crude product was purified on a silica gel column (eluent: hexane/ethyl acetate) to obtain **4a**–**4d** with 55%–65% yield.

2,4-di-(4′-formylphenoxy)-quinoline (4a) With 60% yield, the synthesis started with 50.0 mg (0.25 mmol) of **1** to obtain 55.9 mg of **4a**, which consisted of white solid and mp. 164.9 °C - 165.5 °C. The ^{1}H-NMR (300 MHz, $CDCl_3$): 6.43 (s, 1H, ArH-3), 7.36-7.43 (m, 4H, ArH-2′, 6′, 2″, 6″), 7.51 (td, 1H, J = 7.6, 1.3 Hz, ArH-6), 7.71 (td, 1H, J = 8.1, 1.5 Hz, ArH-7), 7.80 (dd, 1H, J = 8.4, 0.5 Hz, ArH-8), 7.94 (ddd, 2H, J = 8.7, 2.0 Hz, ArH-3′, 5′), 8.03 (ddd, 2H, J = 8.7, 2.0 Hz, ArH-3″, 5″), 8.22 (dd, 1H, J = 8.3, 0.9 Hz, ArH-5), 10.00 (s, 1H, CHO) and 10.04 (s, 1H, CHO). The ^{13}C-NMR (75 MHz, $CDCl_3$): 97.2, 119.7, 120.9, 121.5, 121.7, 125.3, 127.9, 131.1, 131.4, 132.2, 132.9, 133.7, 147.3, 158.7, 159.5, 161.3, 163.2, 190.5 and 191.0. Finally, the HRMS (+ESI) was $C_{23}H_{16}NO_4$ $[M+H]^+$; it requires 370.1079, but has 370.1088.

2,4-di-(2′,6′-Dimethyl-4′-formylphenoxy)-quinoline (4b) With 65 % yield, the synthesis started with 50 mg (0.25 mmol) of **1** to obtain 69.7 mg of **4b**, which consisted of white solid and mp. 120.5 °C - 121.0 °C. The ^{1}H-NMR (300 MHz, $CDCl_3$): 2.16 (s, 6H, $ArCH_3$-2′, 6′), 2.29 (s, 6H, $ArCH_3$-2″, 6″), 5.99 (s, 1H, ArH-3), 7.46-7.51 (m, 1H, ArH-6), 7.63-7.66 (m, 4H, ArH-3′, 5′, 7, 8), 7.76 (s, 2H, ArH-3″, 5″), 8.35 (d, 1H, J = 8.1 Hz, ArH-5), 9.95 (s, 1H, CHO) and 10.02 (s, 1H, CHO). The ^{13}C-NMR (75 MHz, $CDCl_3$): 16.1, 16.7, 91.7, 118.5, 121.6, 124.5, 127.7, 130.2, 130.7, 130.9, 132.2, 132.4, 133.4, 134.2, 147.5, 155.0, 155.6, 161.3, 162.1, 191.3 and 191.7. Finally, the HRMS (+ESI) was $C_{27}H_{24}NO_4$ $[M+H]^+$; it requires 426.1700, but has 426.1690.

2,4-di-(4′-cyanophenoxy)-quinoline (4c) With 55 % yield, the synthesis started with 50 mg (0.25 mmol) of **1** to obtain 50.4 mg of **4c**, which consisted of white solid and mp. 220.9 °C – 221.5 °C. The ^{1}H-NMR (300 MHz, $CDCl_3$): 6.40 (s, 1H, ArH-3), 7.30-7.40 (m, 4H, ArH-2′, 6′, 2″, 6″), 7.51 (td, 1H, J = 7.5, 1.3 Hz, ArH-6), 7.66-7.75 (m, 3H, ArH-7, 3′, 5′), 7.75-7.84 (m, 3H, ArH-8, 3″, 5″) and 8.18 (dd, 1H, J = 8.0, 0.7 Hz, ArH-5). The ^{13}C-NMR (75 MHz, $CDCl_3$): 97.1, 108.2, 109.4, 118.0, 118.6, 119.6, 121.3, 121.6, 122.0, 125.5, 127.8, 131.3, 133.7, 134.7, 147.2, 157.0, 158.1, 161.0 and 163.0. Finally, the HRMS (+ESI) was $C_{23}H_{14}N_3O_2$ $[M+H]^+$; it requires 364.1086, but has 364.1067.

2,4-di-(2′,6′-Dimethyl-4′-cyanophenoxy)-quinoline (4d) With 57 % yield, the synthesis started with 50 mg (0.25 mmol) of **1** to obtain 60.3 g of **4d**, which consisted of white solid and mp. 201.8 °C - 202.4 °C. The ^{1}H-NMR (300 MHz, $CDCl_3$): 2.10 (s, 6H, $ArCH_3$-2′,

6′), 2.24 (s, 6H, ArCH3-2″, 6″), 5.96 (s, 1H, ArH-3), 7.41 (s, 2H, ArH-3′, 5′), 7.46-7.52 (m, 1H, ArH-6), 7.54 (bs, 2H, ArH-3″, 5″), 7.64-7.66 (m, 2H, ArH-7, 8) and 8.32 (d, 1H, J = 8.3 Hz, ArH-5). The ^{13}C-NMR (75 MHz, $CDCl_3$): 16.0, 16.5, 91.6, 102.8, 108.9, 110.2, 118.3, 118.4, 119.0, 121.5, 121.6, 124.7, 126.8, 127.8, 128.5, 130.9, 131.6, 132.4, 132.8, 133.0, 133.3, 147.4, 153.7, 154.1, 161.0 and 162.0 Finally, the HRMS (+ESI) was $C_{27}H_{22}N_3O_2$ $[M+H]^+$; it requires 420.1706, but has 420.1712.

General procedure for the preparation of 2-phenylamino-4-phenoxy-quinoline (**5a–5d and 6a–6d**). A mixture of **2a–2d** (0.5 mmol), 4-aminobenzonitrile (0.65 mmol) for the synthesis of **5a–5d** or 2-amino-5-cyanopyridine (0.65 mmol) for the synthesis of **6a–6d**, $Pd(OAc)_2$ (0.05 mmol), SPhos (0.05 mmol), and Cs_2CO_3 (0.75 mmol) in DMF (20 mL) was stirred, heated at 120 °C for 5–10 h, and cooled. Then, the corresponding solution was evaporated in vacuo. The residue was purified on a silica gel column (eluent: hexane/ethyl acetate) to obtain **5–6**(**a–d**) with 59%–72% yield. The dimers of biquinoline, namely, 4,4′-di-(4′-formylphenoxy)-2,2′-biquinoline (**7a**) and 4,4′-di-(2′,6′-dimethyl-4′-formylphenoxy)-2,2′-biquinoline (**7b**), were found during the syntheses of **5a**, **5b**, **6a**, and **6b**.

4-(4′-formylphenoxy)-2-(4′′-cyanophenyl)-aminoquinoline (**5a**). With 62% yield, the synthesis started with 70.0 mg (0.25 mmol) of **2a** to obtain 55.9 mg of **5a**, which consisted of white solid and mp. 267.1 °C - 268.0 °C. **5a** was obtained in 41.5 % overall yield (2 steps from **1**). The ^{1}H-NMR (300 MHz, DMSO-d6): 6.42 (s, 1H, ArH-3), 7.43 (t, 1H, J = 7.1 Hz, ArH-6), 7.50-7.59 (m, 2H, ArH-2″, 6″), 7.66-7.88 (m, 4H, ArH-3″, 5″, 2′, 6′), 8.04-8.15 (m, 5H, ArH-5, 7, 8, 3′, 5′), 9.80 (bs, 1H, NH), and 10.10 (s, 1H, CHO). The ^{13}C-NMR (75 MHz, DMSO-d6): 97.8, 102.0, 117.6, 117.9, 119.6, 121.2, 123.6, 126.7, 130.7, 132.1, 133.1, 133.5, 145.4, 148.1, 154.1, 159.1, 160.4 and 191.9. Finally, the HRMS (+ESI) was $C_{23}H_{16}N_3O_2$ $[M+H]^+$; it requires 366.1237, but has 366.1229.

4-(2′,6′-Dimethyl-4′-formylphenoxy)-2-(4′′-cyanophenyl)-aminoquinoline (**5b**). With 69% yield, the synthesis started with 40.0 mg (0.13 mmol) of **2b** to obtain 34.8 mg of **5b**, which consisted of white solid and mp. 291.3 °C - 292.2 °C. **5b** was obtained in 47.6 % overall yield (2 steps from **1**). The ^{1}H-NMR (300 MHz, $CDCl_3$): 2.26 (s, 6H, ArCH3-2′, 6′), 5.73 (s, 1H, ArH-3), 6.95 (bs, 1H, NH), 7.46 (td, 1H, J = 7.6, 1.0 Hz, ArH-6), 7.56 (m, 2H, ArH-2″, 6″), 7.70 (s, 2H, ArH-3′, 5′), 7.74 (m, 1H, ArH-7), 7.87 (m, 2H, ArH-3″, 5″), 7.91 (d, 1H, J = 8.6 Hz, ArH-8), 8.30 (dd, 1H, J = 8.2, 1.1 Hz, ArH-5), and 9.96 (s, 1H, CHO). The ^{13}C-NMR (75 MHz, $CDCl_3$): 16.1, 93.1, 104.0, 117.6, 118.2, 119.5, 121.5, 123.9, 127.3, 130.9, 132.5, 133.2, 134.1, 144.6, 148.7, 153.5, 155.2, 160.7, and 191.4. Finally, the HRMS (+ESI) was $C_{25}H_{20}N_3O_2$ $[M+H]^+$; it requires 394.1550, but has 394.1559.

4-(4′-cyanophenoxy)-2-(4′′-cyanophenyl)-aminoquinoline (5c). With 59% yield, the synthesis started with 70.0 mg (0.25 mmol) of **2c** to obtain 53.3 mg of **5c**, which consisted of white solid and mp. 278.5 °C - 278.7 °C. **5c** was obtained in 35.4 % overall yield (2 steps from **1**). The ^{1}H-NMR (300 MHz, $CDCl_3$): 6.59 (s, 1H, ArH-3), 7.17-7.31 (m, 4H, ArH-2′′, 6′′, 2′, 6′), 7.47-7.54 (m, 1H, ArH-6), 7.58-7.80 (m, 7H, ArH-7, 8, 3′′, 5′′, 3′, 5′, NH) and 8.08 (d, 1H, J = 7.9 Hz, ArH-5). The ^{13}C-NMR (75 MHz, $CDCl_3$): 103.9, 108.5, 118.1, 119.8, 119.9, 121.6, 125.9, 126.9, 128.0, 131.1, 133.2, 134.4, 147.8, 148.7, 156.0, 158.7, and 160.2. Finally, the HRMS (+ESI) was $C_{23}H_{15}N_4O$ $[M+H]^+$; it requires 363.1240, but has 363.1232.

4-(2′,6′-Dimethyl-4′-cyanophenoxy)-2-(4′′-cyanophenyl)-aminoquinoline (5d). With 60% yield, the synthesis started with 70.0 mg (0.23 mmol) of **2c** to obtain 53.0 mg of **5d**, which consisted of white solid and mp. 256.5 °C -257.0 °C. **5d** was obtained in 37.2 % overall yield (2 steps from **1**). The ^{1}H-NMR (300 MHz, $CDCl_3$): 2.23 (s, 6H, $ArCH_3$-2′, 6′), 5.81 (s, 1H, ArH-3), 7.41-7.46 (m, 1H, ArH-6), 7.47 (s, 2H, ArH-3′, 5′), 7.56 (dd, 2H, J = 7.1, 1.8 Hz, ArH-2′′, 6′′), 7.71 (m, 2H, ArH-7, NH), 7.87-7.99 (m, 3H, ArH-8, 3′′, 5′′), and 8.27 (dd, 1H, J = 8.2, 1.1 Hz, ArH-5). The ^{13}C-NMR (75 MHz, $CDCl_3$): 16.0, 93.3, 103.6, 109.4, 117.4, 118.2, 119.6, 121.3, 123.8, 127.2, 130.9, 133.0, 133.1, 133.2, 144.8,148.6, 153.8, 154.0, and 160.2. Finally, the HRMS (+ESI) was $C_{25}H_{19}N_4O$ $[M+H]^+$; it requires 391.1553, but has 391.1548.

4-(4'-formylphenoxy)-2-(5''-cyanopyridin-2''ylamino)quinoline (6a). With 60 % yield, the synthesis started with 70 mg (0.25 mmol) of **2a** to obtain 54.2 mg of **6a**, which consisted of white solid and mp. 248.0 °C – 249.0 °C. **5a** was obtained in 40 % overall yield (2 steps from **1**). The ^{1}H-NMR (300 MHz, $CDCl_3$): 6.77 (s, 1H, ArH-3), 7.34 (d, 2H, J = 8.6 Hz, ArH-2′, 6′), 7.46 (td, 1H, J = 7.6, 0.8 Hz, ArH-3′′), 7.74 (td, 1H, J = 7.7, 1.4 Hz, ArH-6), 7.83-7.95 (m, 3H, ArH-7, 8, NH), 7.97-8.06 (m, 2H, ArH-3′, 5′), 8.14 (dd, 1H, J = 8.6, 0.8 Hz, ArH-5), 8.33-8.49 (m, 2H, ArH-4′′, 6′′)and 10.0 (s, 1H, CHO). The ^{13}C-NMR (75 MHz, $CDCl_3$): 98.6, 112.1, 117.5, 120.6, 121.7, 124.7, 127.2, 130.4, 131.3, 132.1, 133.5, 140.8, 151.8, 152.2, 161.7 and 190.5. Finally, the HRMS (+ESI) was $C_{22}H_{15}N_4O_2$ $[M+H]^+$; it requires 367.1190, but has 367.1176.

4-(2',6'-Dimethyl-4'-formylphenoxy)-2-(5''-cyanopyridin-2''ylamino)quinoline (6b). With 65 % yield, the synthesis started with 50 mg (0.16 mmol) of **2b** to obtain 41.1 mg of **6b**, which consisted of white solid and mp. 275.7 °C – 276.5 °C. **6b** was obtained in 45 % overall yield (2 steps from **1**). The ^{1}H-NMR (300 MHz, DMSO-d6): 2.20 (s, 6H, $ArCH_3$-2′, 6′), 6.58 (s, 1H, ArH-3), 7.52 (t, 1H, J = 7 Hz, ArH-3′′), 7.77 (td, 1H, J = 7.8, 16 Hz, ArH-6), 7.82-7.91 (m, 3H, ArH-7, 3′, 5′), 8.14 (dd, 1H, J = 8.8, 2.3 Hz, ArH-8), 8.29 (d, 1H, J = 7.5 Hz, ArH-5), 8.57 (d, 1H, J = 1.9 Hz, ArH-4′′), 8.65 (d, 1H, J = 8.9 Hz, ArH-6′′), 10.03 (s, 1H, CHO)

and 10.37 (s, 1H, NH). The ^{13}C-NMR (75 MHz, DMSO-d6): 15.6, 94.8, 97.5, 100.3, 111.8, 117.2, 117.9, 121.3, 124.2, 126.8, 130.7, 130.9, 131.9, 134.1, 141.1, 145.9, 151.9, 153.4, 154.5, 155.9, 159.7 and 160.4. Finally, the HRMS (+ESI) was $C_{24}H_{19}N_4O_2$ [M+H]$^+$; it requires 395.1502, but has 395.1500.

4-(4′-cyanophenoxy)- 2-(5''-cyanopyridin-2''ylamino)quinoline (**6c**). With 60 % yield, the synthesis started with 50 mg (0.18 mmol) of **2c** to obtain 38.8 mg of **5c**, which consisted of white solid and mp. 225.7 °C – 225.9 °C. **6c** was obtained in 36 % overall yield (2 steps from **1**). The ^{1}H-NMR (300 MHz, $CDCl_3$): 6.86 (s, 1H, ArH-3), 7.30 (d, 2H, J = 8.8 Hz, ArH-2′, 6′), 7.46 (td, 1H, J = 7.6 Hz, ArH-3′′), 7.70-7.81 (m, 3H, ArH-6, 3′, 5′), 7.83-7.94 (m, 2H, ArH-7, 8), 8.11 (dd, 1H, J = 8.4, 0.7, ArH-5), 8.30 (bs, 2H, ArH-4′′, NH) and 8.46 (d, 1H, J = 1.7 Hz, ArH-6′′). The ^{13}C-NMR (75 MHz, $CDCl_3$): 98.8, 102.0, 112.3, 117.4, 118.1, 120.9, 121.7, 124.9, 131.5, 134.6, 140.8, 140.9 and 151.7. Finally, the HRMS (+ESI) was $C_{22}H_{14}N_5O$ [M+H]$^+$; it requires 364.1193, but has 364.1193.

4-(2′,6′-Dimethyl-4′-cyanophenoxy)-2-(5''-cyanopyridin-2''ylamino)quinoline (**6d**). With 72% yield, the synthesis started with 50 mg (0.16 mmol) of **2c** to obtain 45.6 mg of **5d**, which consisted of white solid and mp. 229.4 °C - 230.3 °C. **5d** was obtained in 45 % overall yield (2 steps from **1**). The ^{1}H-NMR (300 MHz, $CDCl_3$): 2.21 (s, 6H, $ArCH_3$-2′, 6′), 6.12 (s, 1H, ArH-3), 7.43-7.57 (m, 3H, ArH-3′′, 3′, 5′), 7.75 (td, 1H, J = 7.8, 1.4 Hz, ArH-6), 7.82-7.97 (m, 2H, ArH-7, 8), 8.23-8.44 (m, 3H, ArH-5, 4′′, NH) and 8.58 (d, 1H, J = 8.3 Hz, ArH-6′′). The ^{13}C-NMR (75 MHz, $CDCl_3$): 15.9, 93.8, 101.7, 110.0, 112.3, 117.5, 117.7, 118.2, 121.5, 124.5, 127.2, 131.1, 132.9, 133.1, 140.8, 148.3, 151.6, 152.5, 153.6, 155.7 and 160.6. Finally, the HRMS (+ESI) was $C_{24}H_{18}N_5O$ [M+H]$^+$; it requires 392.1506, but has 392.1494.

4,4′-di-(4′-formylphenoxy)-2,2′-biquinoline (**7a**) With the little amount during the synthesis process to produce compound **5a** and **6a**, which consisted of white solid and mp. 288.7 °C – 289.3 °C. The ^{1}H-NMR (300 MHz, $CDCl_3$): 7.33-7.42 (m, 4H, 2×ArH-2′, 6′), 7.59 (td, 2H, J = 7.7, 1.1 Hz, 2×ArH-3), 7.76 (td, 2H, J = 7.7, 1.4 Hz, 2×ArH-6), 7.98-8.06 (m, 4H, 2×ArH-3′, 7′), 8.11 (d, 2H, J = 8.3 Hz, 2×ArH-8), 8.21-8.30 (m, 4H, 2×ArH-5, 5′) and 10.0 (s, 2H, 2×CHO). The ^{13}C-NMR (75 MHz, $CDCl_3$): 105.1, 120.1, 121.7, 122.1, 127.2, 129.8, 130.4, 130.5, 132.2, 133.1, 149.6, 157.0, 160.4, 160.7 and 190.8. Finally, the HRMS (+ESI) was $C_{32}H_{21}N_2O_4$ [M+H]$^+$; it requires 497.1496, but has 497.1491.

4,4′-di-(2',6'-Dimethyl-4'-formylphenoxy)-2,2′-biquinoline (**7b**) With the little amount during the synthesis process to produce compound **5b** and **6b**, which consisted of white solid and mp. 300.0 °C (decomposed). The ^{1}H-NMR (300 MHz, $CDCl_3$ + CD_3OD): 2.27 (s, 12H, 2×$ArCH_3$-2′, 6′), 7.55 (s, 2H, 2×ArH-3), 7.59-7.69 (m, 2H, 2×ArH-6), 7.71-7.86 (m, 6H, 2×ArH-7, 3′, 5′), 8.04 (d, 2H, J = 8.3 Hz, 2×ArH-8), 8.45 (d, 2H, J = 8.1 Hz, 2×ArH-5)

and 10.0 (s, 2H, 2×CHO). The ^{13}C-NMR (75 MHz, $CDCl_3$ + CD_3OD): 16.1, 100.0, 120.5, 121.4, 126.7, 129.3, 130.3, 130.9, 132.4, 133.9, 149.0, 155.3, 157.1 and 160.1. Finally, the HRMS (+ESI) was $C_{36}H_{29}N_2O_4$ [M+H]$^+$; it requires 553.2122, but has 553.2116.

General procedure for the preparation of 2-phenoxy-4-phenylamine-quinoline (8a–8d). A mixture of 4-chloro-2-phenoxyquinoline **3a**–**3d** (0.5 mmol), 4-aminobenzonitrile (0.65 mmol), $Pd(OAc)_2$ (0.05 mmol), SPhos (0.05 mmol), and Cs_2CO_3 (0.75 mmol) in DMF (20 mL) was stirred, heated at 120 °C for 6 h, and cooled. The corresponding solution was evaporated in vacuo. The residue was purified on a silica gel column (eluent: hexane/ethyl acetate) to obtain **8a**–**8d**) with 57%–66% yield.

2-(4′-formylphenoxy)-4-(4′′-cyanophenyl)-aminoquinoline (8a). With 58% yield, the synthesis started with 50 mg (0.18 mmol) of **3a** to obtain 37.4 mg of **8a**, which consisted of white solid and mp. 238.5 °C – 238.9 °C. **8a** was obtained in 6 % overall yield (2 steps from **1**). The ^{1}H-NMR (400 MHz, $CDCl_3$ + CD_3OD): 6.95 (s, 1H, ArH-3), 7.36-7.40 (m, 2H, ArH-2″, 6″), 7.41-7.45 (m, 3H, ArH-2′, 6′, NH), 7.50 (td, 1H, J = 6.0, 2.0 Hz, ArH-6), 7.64-7.71 (m, 3H, ArH-7, 3″, 5″), 7.79 (d, 1H, J = 8.0, 2.0 Hz, ArH-8), 7.95 (dt, 2H, J = 8.0, 2.0 Hz, ArH-3′, 5′), 8.13 (d, 1H, J = 8.0 Hz, ArH-5), and 9.97 (s, 1H, CHO). The ^{13}C-NMR (75 MHz, $CDCl_3$ + CD_3OD): 95.0, 104.6, 113.8, 118.8, 119.2, 119.8, 120.2, 121.1, 124.6, 127.5, 130.3, 131.4, 132.1, 133.4, 145.3, 146.9, 149.5, 159.6, 161.3 and 191.4. Finally, the HRMS (+ESI) was $C_{23}H_{16}N_3O_2$ [M+H]$^+$; it requires 366.1237, but has 366.1244.

2-(2′,6′-Dimethyl-4′-formylphenoxy)-4-(4′′-cyanophenyl)-aminoquinoline (8b). With 66% yield, the synthesis started with 50 mg (0.16 mmol) of **3b** to obtain 41.6 mg of **8b**, which consisted of white solid and mp. 200.0 °C – 201.3 °C. **8b** was obtained in 9 % overall yield (2 steps from **1**). The ^{1}H-NMR (300 MHz, $CDCl_3$): 2.21 (s, 6H, $ArCH_3$-2′, 6′), 6.86 (s, 1H, NH), 6.98 (s, 1H, ArH-3), 7.35 (s, 1H, ArH-3′), 7.36 (s, 1H, ArH-5′) 7.42 (td, 1H, J = 7.6, 1.5 Hz, ArH-6′), 7.60 (td, 1H, J = 7.5, 1.2 Hz, ArH-7), 7.63-7.73 (m, 5H, ArH-8, 2″, 3″, 5″, 6″), 7.85 (d, 1H, J = 7.8 Hz, ArH-5), 9.97 (s, 1H, CHO). The ^{13}C-NMR (75 MHz, $CDCl_3$): 16.8, 94.3, 105.9, 118.6, 118.9, 119.7, 119.8, 124.4, 128.9, 130.3, 132.6, 133.5, 133.9, 144.8, 147.7, 147.8, 155.7, 161.3 and 191.8. Finally, the HRMS (+ESI) was $C_{25}H_{20}N_3O_2$ [M+H]$^+$; it requires 394.1550, but has 394.1542.

2-(4′-cyanophenoxy)-4-(4′′-cyanophenyl)-aminoquinoline (8c). With 57% yield, the synthesis started with 50 mg (0.18 mmol) of **3c** to obtain 36.8 mg of **8c**, which consisted of white solid and mp. 187.5 °C – 188.0 °C. **8c** was obtained in 5 % overall yield (2 steps from **1**). The ^{1}H-NMR (400 MHz, CD_3OD): 6.87 (s, 1H, ArH-3), 7.37 (d, 2H, J = 8.8 Hz, ArH-2″, 6″), 7.45–7.47 (m, 1H, ArH-6), 7.49 (d, 2H, J = 8.8 Hz, ArH-2′, 6′), 7.56–7.58 (m, 1H, ArH-7), 7.61–7.65 (m, 1H, ArH-8), 7.76 (d, 2H, J = 8.8 Hz, ArH-3′′, 5′′), 7.85 (d, 2H, J = 8.8 Hz,

ArH-3′, 5′), 8.25 (d, 1H, J = 8.0 Hz, ArH-5) and 9.49 (s, 1H, NH). The ^{13}C-NMR (100 MHz, DMSO-d6): 95.1, 104.4, 107.3, 119.2, 119.7, 119.7, 120.5, 122.6, 122.9, 124.9, 128.2, 131.0, 134.2, 134.5, 146.1, 147.2, 149.7, 158.0 and 161.9 Finally, the HRMS (+ESI) was $C_{23}H_{15}N_4O$ $[M+H]^+$; it requires 363.1240, but has 363.1245.

2-(2′,6′-Dimethyl-4′-cyanophenoxy)-4-(4″-cyanophenyl)-aminoquinoline (8d). With 60% yield, the synthesis started with 50 mg (0.16 mmol) of **3d** to obtain 37.9 mg of **7d**, which consisted of white solid and mp. 201.3 °C – 202.0 °C. **8d** was obtained in 3 % overall yield (2 steps from **1**). The ^{1}H-NMR (400 MHz, $CDCl_3$): 2.16 (s, 6H, $ArCH_3$-2′, 6′), 6.98 (s, 1H, ArH-3), 7.02 (bs, 1H, NH), 7.38-7.44 (m, 5H, ArH-6, 2″, 3″, 5″, 6″), 7.58-7.65 (m, 2H, ArH-7, 8), 7.69 (d, 2H, J = 12 Hz, ArH-3′, 5′) and 7.89 (d, 1H, J = 8.0 Hz, ArH-5). The ^{13}C-NMR (100 MHz, $CDCl_3$): 16.0, 94.0, 105.8, 108.7, 114.4, 118.7, 118.9, 119.1, 119.8, 120.0, 124.5, 128.8, 130.3, 132.4, 133.1, 133.8, 133.8, 144.8, 147.5, 148.0, 154.3, and 161.1. Finally, the HRMS (+ESI) was $C_{25}H_{19}N_4O$ $[M+H]^+$; it requires 391.1553, but has 391.1561.

General procedure for the preparation of 9b, 10b, and 11b. Potassium tert-butoxide (1.50 mmol) was added to an ice-cooled solution of diethyl cyanomethyl phosphonate (1.50 mmol) in THF (20 mL). The mixture was stirred at 0 °C for 30 min and then at room temperature for another 30 min. A solution of 4-(4′-formylphenoxy)-2-arylamino-quinoline (**5b** and **6b**) or 2-(4′-formylphenoxy)-4-phenylamino-quinoline (**8b**, 1 mmol) in THF (13 mL) was added dropwise to the reaction mixture. The solution was continued for 8–10 h. After the reaction was completed, the corresponding solution was added with water and extracted with ethyl acetate. The organic layer was dried over Na_2SO_4 and concentrated under reduced pressure. The residue was purified on a silica gel column (eluent: hexane/ethyl acetate) to obtain **9b**, **10b**, and **11b** with 56%–64% yield. The E:Z isomer ratio for **9b** and **10b** was 7:3, whereas **11b** was inseparable.

4-(4′-(2′′-(E, Z)-cyanovinyl)-2′,6′-dimethyl-phenoxy)-2-(4′′-cyanophenyl)-aminoquinoline (9b). With 64% yield (E: Z isomer; 7: 3), the synthesis started with 100 mg (0.25 mmol) of **5b** to obtain 67.9 mg of **9b** which was obtained in 30 % overall yield (3 steps from **1**).

In the case of Z-isomer; Z-isomer consisted of white solid and mp. 217.3 °C – 217.9 °C. The ^{1}H-NMR (300 MHz, $CDCl_3$): 2.20 (s, 6H, $ArCH_3$-2′, 6′), 5.47 (d, 1H, J = 12.1, Vinyl-H), 5.74 (s, 1H, ArH-3), 6.78 (bs, 1H, NH), 7.12 (d, 1H, J = 12.1 Hz, Vinyl-H), 7.45 (td, 1H, J = 7.4, 1.1 Hz, ArH-6), 7.52-7.66 (m, 4H, ArH-3′, 5′, 2″, 6″), 7.71 (td, 1H, J = 7.7, 1.4 Hz, ArH-7), 7.81-7.94 (m, 3H, ArH-8, 3″, 5″) and 8.29 (dd, 1H, J = 8.3, 1.0 Hz, ArH-5). The ^{13}C-NMR (75 MHz, $CDCl_3$): 16.1, 93.0, 95.2, 104.0, 117.3, 117.7, 118.2, 119.5, 121.6, 123.9, 127.2, 128.4,

130.1, 130.9, 131.5, 131.9,133.2, 144.6, 147.9, 148.6, 152.1, 153.5 and 161.1 Finally, the HRMS (+ESI) was $C_{27}H_{21}N_4O$ $[M+H]^+$; it requires 417.1710, but has 417.1712.

In the case of E-isomer; E-isomer consisted of white solid and mp. 214.4 °C – 215.1 °C. The ^{1}H-NMR (300 MHz, $CDCl_3+CD_3OD$): 2.20 (s, 6H, $ArCH_3$-2′, 6′), 5.82 (s, 1H, ArH-3), 5.92 (d, 1H, J = 16.6 Hz, Vinyl-H), 7.30 (s, 2H, ArH-3′, 5′), 7.41 (d, 1H, J = 16.6 Hz, Vinyl-H), 7.40-7.48 (m, 1H, ArH-6), 7.56 (d, 2H, J = 8.7 Hz, ArH-2″, 6″), 7.66-7.76 (m, 1H, ArH-7), 7.84-7.94 (m, 3H, ArH-8, 3″, 5″) and 8.28 (dd, 1H, J = 8.1, 1.0 Hz, ArH-5). The ^{13}C-NMR (75 MHz, $CDCl_3+CD_3OD$): 15.8, 93.2, 95.9, 102.9, 117.48, 117.9, 119.6, 121.3, 123.5, 126.8, 128.2, 130.6, 131.2, 132.1, 133.0, 145.1, 148.4, 149.8, 152.5, 154.0 and 160.7. Finally, the HRMS (+ESI) was $C_{27}H_{21}N_4O$ $[M+H]^+$; it requires 417.1710, but has 417.1712.

4-(4′-(2″-(E, Z)-cyanovinyl)-2′,6′-dimethyl-phenoxy)-2-(5″-cyanopyridin-2″ylamino)-aminoquinoline (10b). With 60% yield (E: Z isomer; 7: 3), the synthesis started with 100 mg (0.25 mmol) of **6b** to obtain 63.5 mg of **10b** which was obtained in 27 % overall yield (3 steps from **1**).

In the case of Z-isomer; Z-isomer consisted of white solid and mp. 239.6 °C – 239.9 °C. The ^{1}H-NMR (300 MHz, $CDCl_3$): 2.20 (s, 6H, $ArCH_3$-2′, 6′), 5.49 (d, 1H, J = 12.1 Hz, Vinyl-H), 6.11 (s, 1H, ArH-3), 7.13 (d, 1H, J = 12.1 Hz, Vinyl-H), 7.49 (t, 1H, J = 7.7 Hz, ArH-3″), 7.64 (s, 2H, ArH-3′, 5′), 7.74 (td, 1H, J = 7.6, 1.2 Hz, ArH-6), 7.81-7.95 (m, 3H, ArH-5, 7, 8), 8.07 (bs, 1H, NH), 8.27-8.43 (m, 3H, ArH-5, 4″) and 8.58 (d, 1H, J = 8.6 Hz, ArH-6″). The ^{13}C-NMR (75 MHz, $CDCl_3$): 16.1, 93.9, 95.2, 96.5, 112.2, 117.2, 117.6, 118.0, 121.7, 124.3, 127.2, 128.4, 130.1, 131.0, 131.6, 131.8, 140.7, 147.7, 148.3, 149.5, 151.6, 152.0, 152.5, 155.7 and 161.2. Finally, the HRMS (+ESI) was $C_{26}H_{20}N_5O$ $[M+H]^+$; it requires 418.1662, but has 418.1657.

In the case of E-isomer; E-isomer consisted of white solid and mp. 236.6 °C – 237.4 °C. The ^{1}H-NMR (300 MHz, $CDCl_3+CD_3OD$): 2.21 (s, 6H, $ArCH_3$-2′, 6′), 5.93 (d, 1H, J = 16.7 Hz, Vinyl-H), 6.28 (s, 1H, ArH-3), 7.32 (s, 2H, ArH-3′, 5′), 7.41 (d, 1H, J = 16.7 Hz, Vinyl-H), 7.46-7.55 (td, 1H, J = 7.6, 1.0 Hz, ArH-3″), 7.69-7.78 (m, 1H, ArH-6), 7.81-7.93 (m, 2H, ArH-7, 8), 8.28-8.39 (m, 2H, ArH-5, 4″) and 8.53 (s, 1H, ArH-6″). The ^{13}C-NMR (75 MHz, $CDCl_3+CD_3OD$): 15.8, 96.0, 100.9, 117.5, 117.9, 121.5, 124.2, 128.2, 129.1, 130.8, 131.2, 131.9, 140.5, 149.7, 151.3, 152.3 and 160.9. Finally, the HRMS (+ESI) was $C_{26}H_{20}N_5O$ $[M+H]^+$; it requires 418.1662, but has 418.1657.

2-(4′-(2″-(E, Z)-cyanovinyl)-2′,6′-dimethyl- phenoxy)-4-(4″-cyanophenyl)-aminoquinoline (11b). With 56 % yield, the synthesis started with 60 mg (0.15 mmol) of **8b** to obtain 35.6 mg of **11b**, which consisted of white solid and mp. 180.7 °C – 182.3 °C. **10b** was obtained in 5 % overall yield (3 steps from **1**). The ^{1}H-NMR (300 MHz, $CDCl_3$,

E, Z mixture): 2.16 (s, 6H, ArCH$_3$-2′, 6′), 5.80 (d, 1H, J = 16.5, Vinyl-H), 6.89-6.99 (m, 1H, ArH-3), 7.20 (s, 1H, ArH-3′, 5′), 7.29-7.39 (m, 3H, Vinyl-H, ArH-2″, 6″), 7.54-7.63 (m, 2H, ArH-3″, 5″), 7.63-7.70 (m, 3H, ArH-6, 7, 8) and 7.86 (d, 1H, J = 8.3 Hz, ArH-5). The ^{13}C-NMR (75 MHz, CDCl$_3$, E, Z mixture): 16.8, 93.8, 94.2, 94.3, 95.1, 105.6, 105.7, 117.6, 118.5, 118.7, 119.0, 119.6, 119.7, 119.9, 124.3, 127.8, 128.8, 129.5, 130.3, 130.4, 130.6, 132.1, 132.3, 133.9, 144.9, 147.7, 148.3, 150.4, 152.6, 153.1, 161.5 and 161.6. Finally, the HRMS (+ESI) was $C_{27}H_{21}N_4O$ [M+H]$^+$; it requires 417.1710, but has 417.1701.

Scheme 1

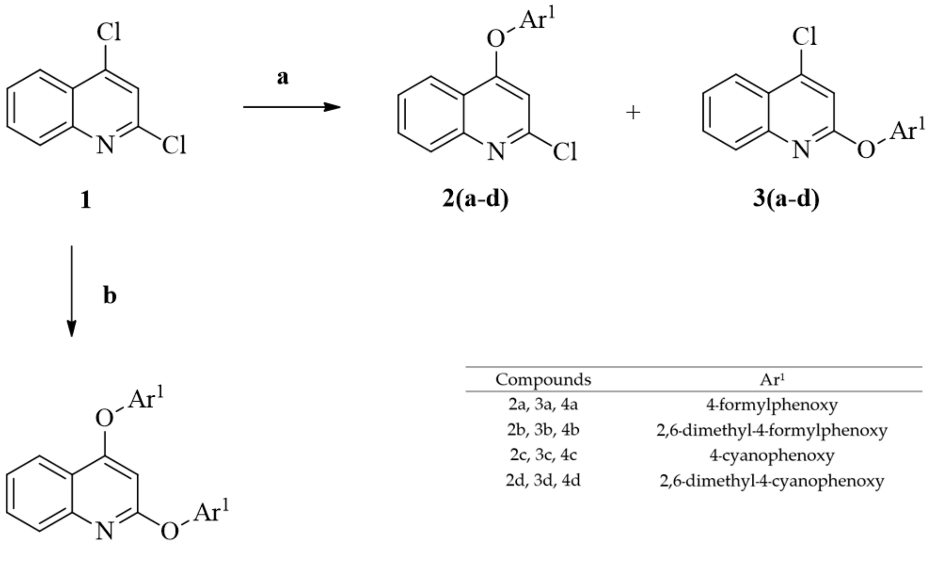

Compounds	Ar^1
2a, 3a, 4a	4-formylphenoxy
2b, 3b, 4b	2,6-dimethyl-4-formylphenoxy
2c, 3c, 4c	4-cyanophenoxy
2d, 3d, 4d	2,6-dimethyl-4-cyanophenoxy

Synthesis of 2-chloro-4-phenoxyquinoline (**2a**–**2d**), 4-chloro-2-phenoxyquinoline (**3a**–**3d**), 2,4-diphenoxyquinoline (**4a**–**4d**). Reagents and conditions: a. hydroxyl benzene (Ar^1–OH, 1.1 eq.), Cs_2CO_3 (2.0 eq), DMF, 80 °C sealed tube; b. hydroxyl benzene (Ar^1–OH, 2.1 eq.), Cs_2CO_3 (2.0 eq.), DMF, 120 °C sealed tube.

4-(4′-formylphenoxy)-2-chloroquinoline (2a)

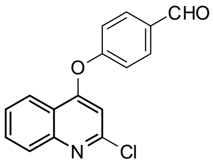

Analysis:

Mp. 113.9-114.2 °C

^{1}H-NMR (300 MHz, $CDCl_3$): 6.64 (s, 1H, ArH-3), 7.35 (d, 2H, J = 8.7 Hz, ArH-2′, 6′), 7.61 (td, 1H, J = 7.8, 1.2 Hz, ArH-6), 7.81 (td, 1H, J = 7.8, 1.2 Hz, ArH-7), 8.04 (m, 3H, ArH-8, 3′, 5′), and 8.26 (dd, 1H, J = 8.4, 0.9 Hz, ArH-5), and 10.1 (s, 1H, CHO)

^{13}C-NMR (75 MHz, $CDCl_3$): 106.5, 120.4, 121.0, 121.8, 126.9, 128.5, 131.6, 132.3, 134.0, 149.0, 151.0, 159.1, 161.9, 190.5

HRMS (+ESI): $C_{16}H_{11}ClNO_2$ $[M+H]^+$ requires 284.0478, found 284.0465

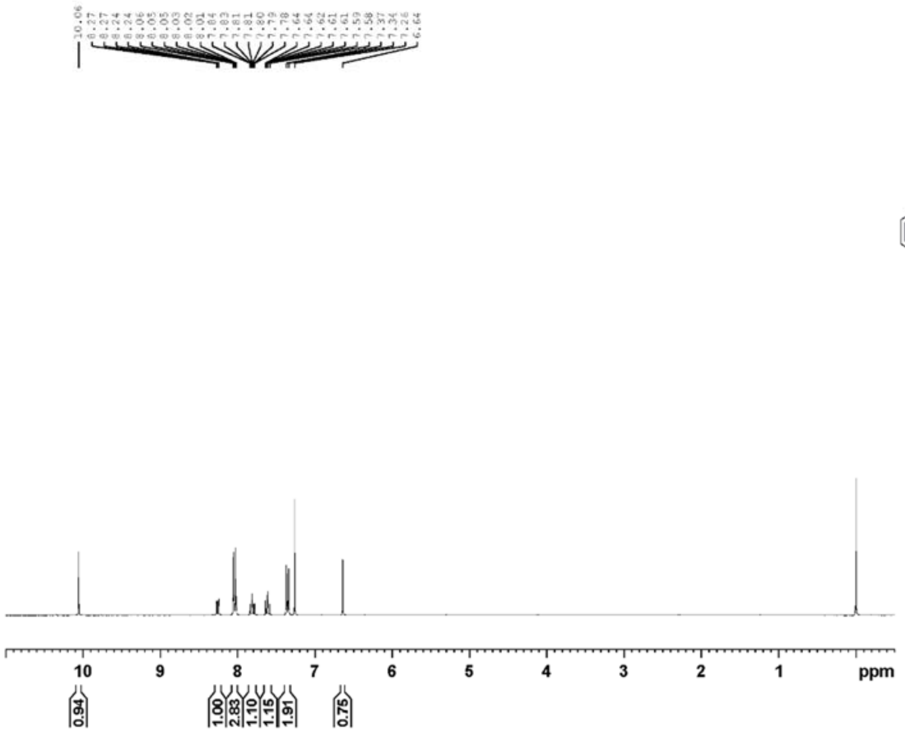

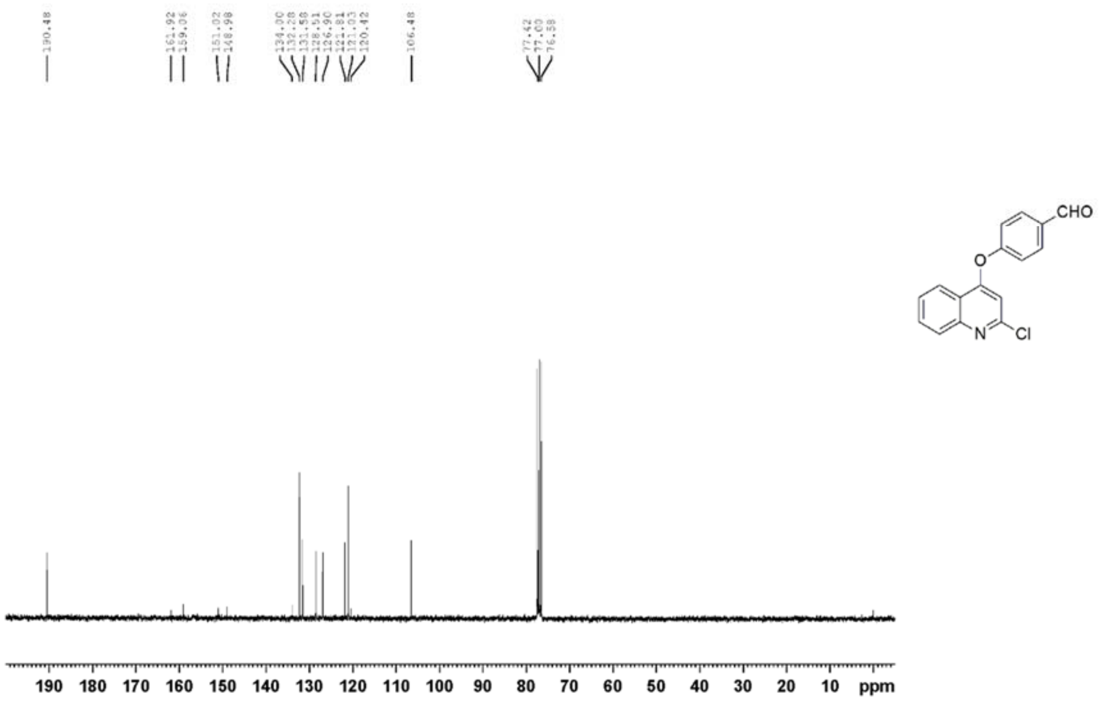

190.48
161.92
159.06
151.02
148.98
134.00
132.28
131.58
128.51
126.90
121.81
121.03
120.42
106.48
77.42
77.00
76.58
CHO
O
N
Cl
190 180 170 160 150 140 130 120 110 100 90 80 70 60 50 40 30 20 10 ppm

4-(2′,6′-Dimethyl-4′-formylphenoxy)-2-chloroquinoline (2b)

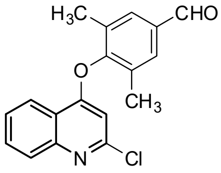

Analysis:

Mp. 154.6-156.9 °C

^{1}H-NMR (300 MHz, $CDCl_3$): 2.23 (s, 6H, $ArCH_3$-2′, 6′), 6.20 (s, 1H, ArH-3), 7.65 (td, 1H, J = 7.5, 1.2 Hz, ArH-6), 7.75 (s, 2H, ArH-3′, 5′), 7.82 (td, 1H, 7.5, 1.2 Hz, ArH-7), 8.04 (dd, 1H, 8.3, 0.9 Hz, ArH-8), and 8.4 (dd, 1 H, 8.3, 0.9 Hz, ArH-5), and 10.0 (s, 1H, CHO)

^{13}C-NMR (75 MHz, $CDCl_3$): 16.1, 102.9, 119.5, 121.7, 126.7, 128.5, 131.5, 132.1, 134.4, 148.8, 151.3, 154.6, 161.0, 191.2

HRMS (+ESI): $C_{18}H_{15}ClNO_2$ $[M+H]^+$ requires 312.0791, found 312.0791

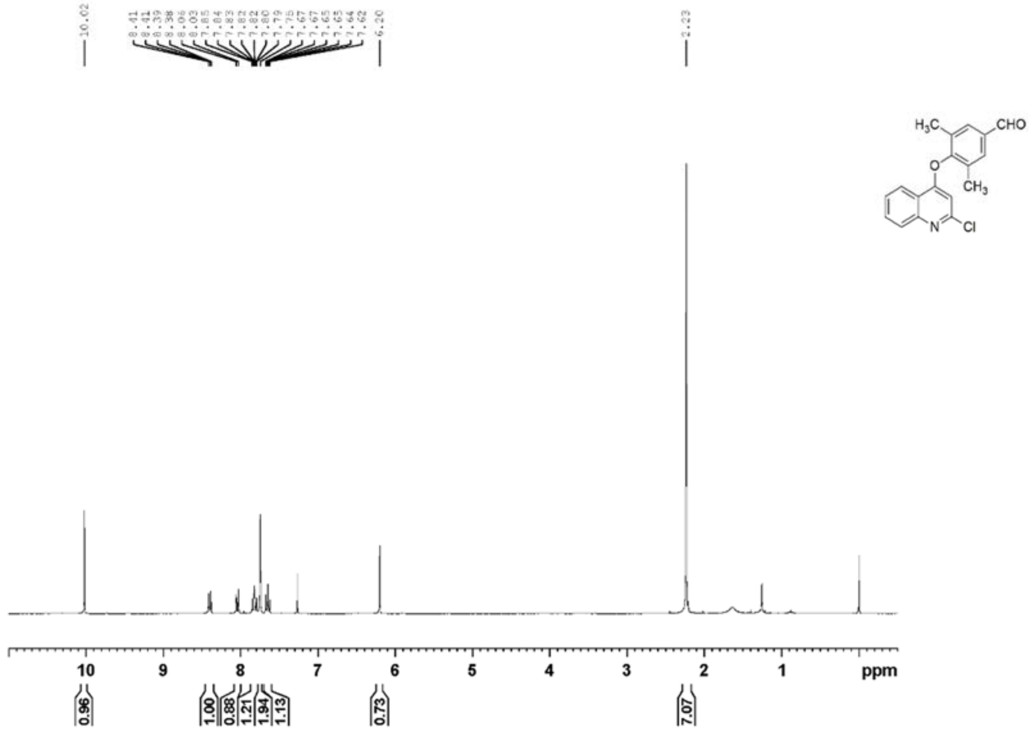

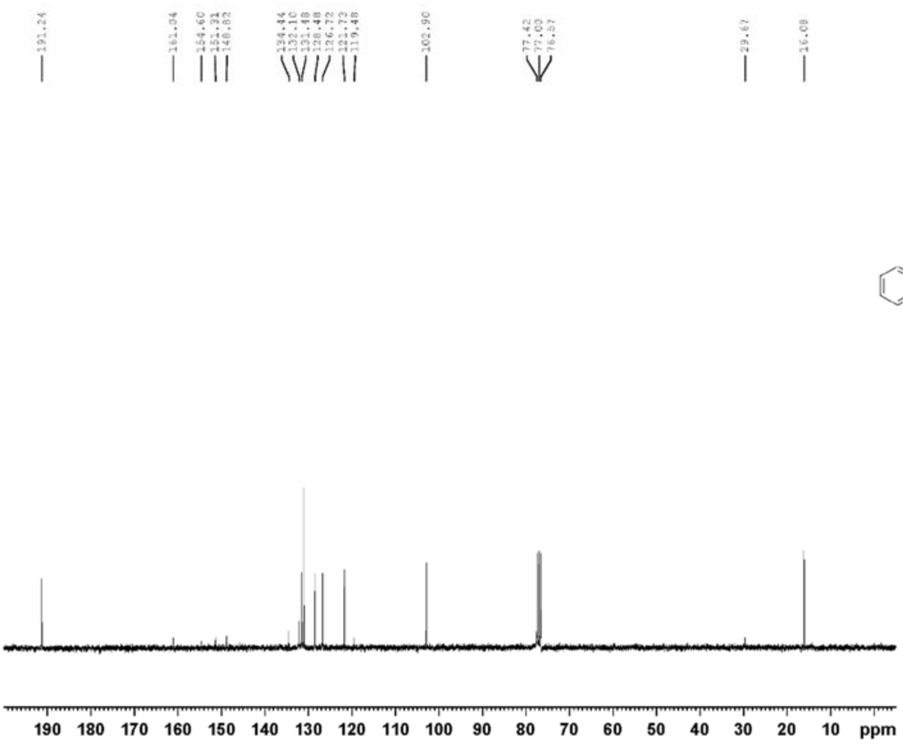

191.24
161.04
154.60
151.31
148.82
134.44
132.10
131.48
128.48
126.72
121.73
119.48
102.90
77.42
77.03
76.57
29.67
16.08
190 180 170 160 150 140 130 120 110 100 90 80 70 60 50 40 30 20 10 ppm

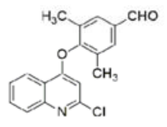

H_3C
CHO
O
CH_3
N
Cl

4-(4′-cyanophenoxy)-2-chloroquinoline (2c)

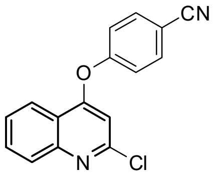

Analysis:

Mp. 214.1-215.0 °C

^{1}H-NMR (300 MHz, $CDCl_3$): 6.63 (s, 1H, ArH-3), 7.32 (d, 2H, J = 8.7 Hz, ArH-2′, 6′), 7.60 (t, 1H, J = 7.2 Hz, ArH-6), 7.78-7.83 (m, 3H, ArH-7, 3′, 5′), 8.02 (d, 1H, J = 8.4 Hz, ArH-8), and 8.22 (d, 1H, J = 8.4 Hz, ArH-5)

^{13}C-NMR (75 MHz, $CDCl_3$): 106.4, 109.6, 117.9, 119.6, 120.2, 121.2, 121.6, 126.9, 128.4, 131.5, 134.4, 134.7, 148.8, 150.8, 157.6, 161.5

HRMS (+ESI): $C_{16}H_{10}ClN_2O$ $[M+H]^+$ requires 281.0482, found 281.0470

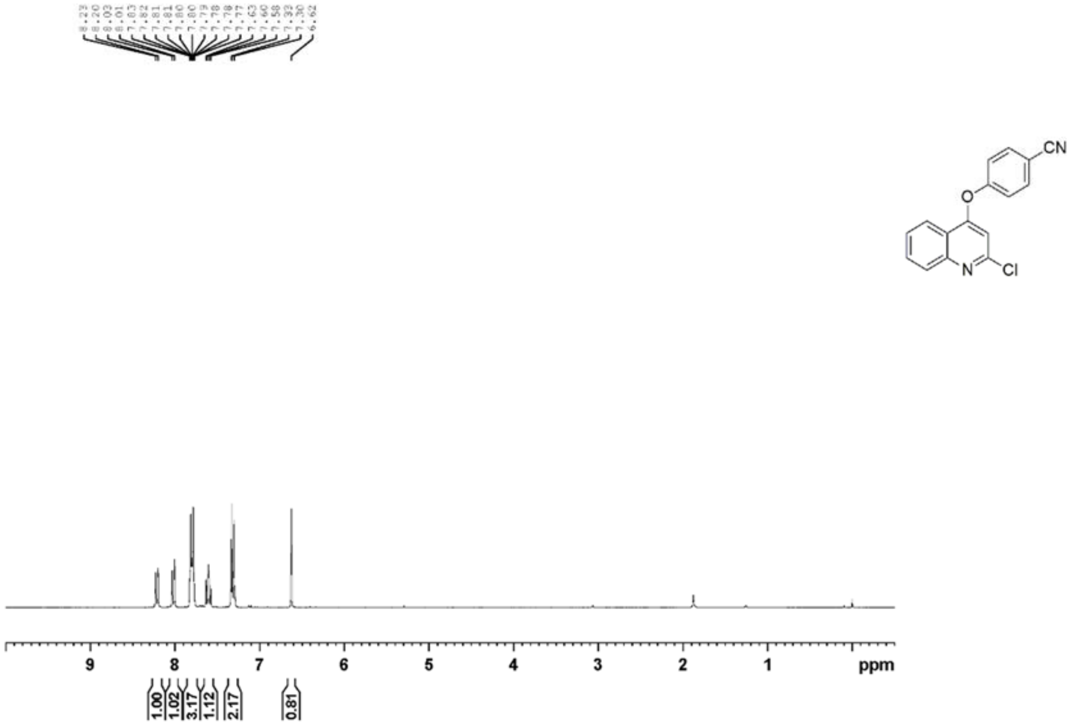

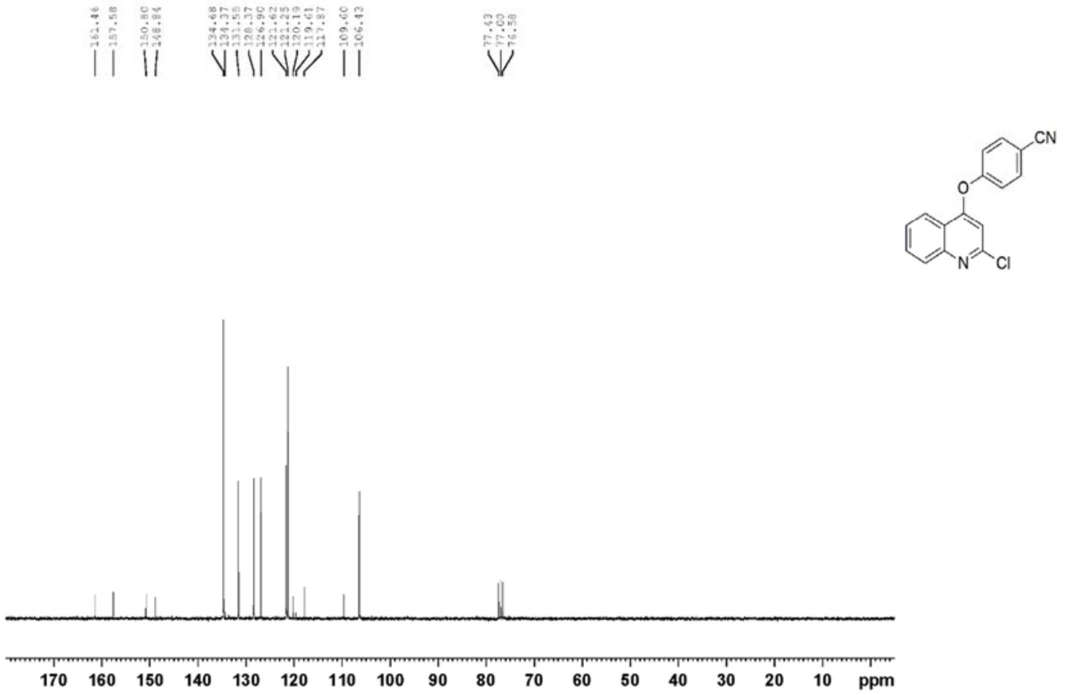

4-(2′,6′-Dimethyl-4′-cyanophenoxy)-2-chloroquinoline (2d)

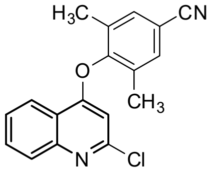

Analysis:

Mp. 184.3-185.0 °C

^{1}H-NMR (300 MHz, $CDCl_3$): 2.20 (s, 6H, $ArCH_3$-2′, 6′), 6.20 (s, 1H, ArH-3), 7.53 (s, 2H, ArH-3′, 5′), 7.65 (td, 1H, J = 9.2, 1.5 Hz, ArH-6), 7.83 (td, 1H, J = 9.2, 1.5 Hz, ArH-7), 8.04 (dd, 1H, J = 8.3, 1.2 Hz, ArH-8), and 8.38 (dd, 1H, J = 8.3, 1.2 Hz, ArH-5)

^{13}C-NMR (75 MHz, $CDCl_3$): 15.9, 102.7, 110.4, 118.1, 119.3, 121.6, 126.8, 128.4, 131.5, 132.7, 133.3, 148.8, 151.1, 153.2, 160.7

HRMS (+ESI): $C_{18}H_{14}ClN_2O$ $[M+H]^+$ requires 309.0795, found 309.0783

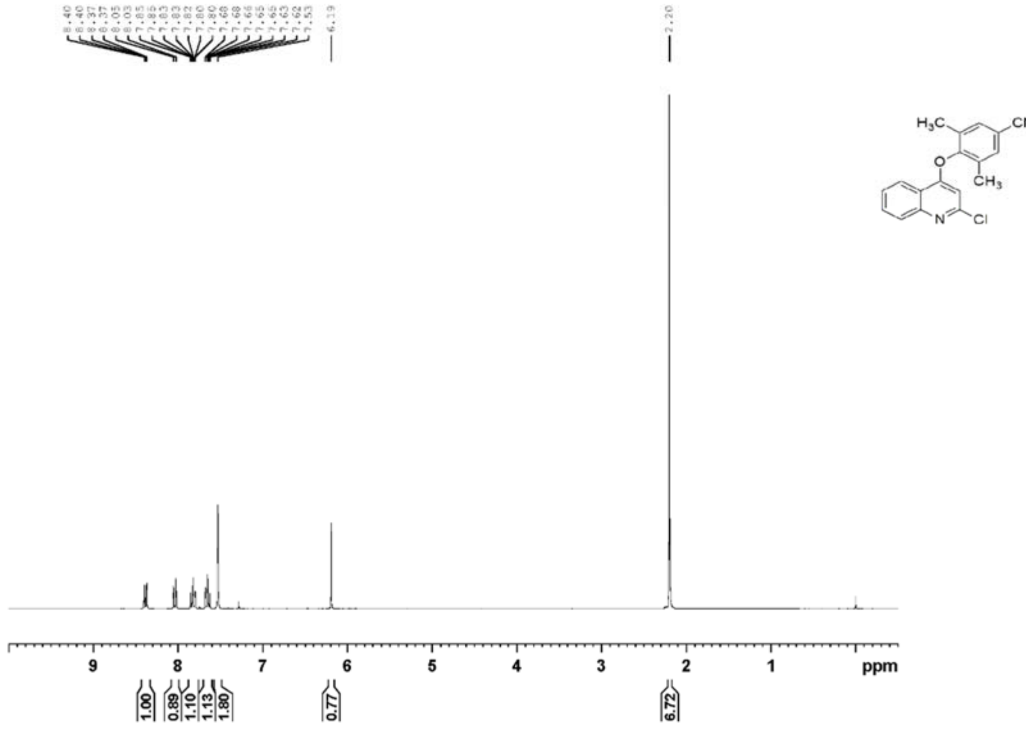

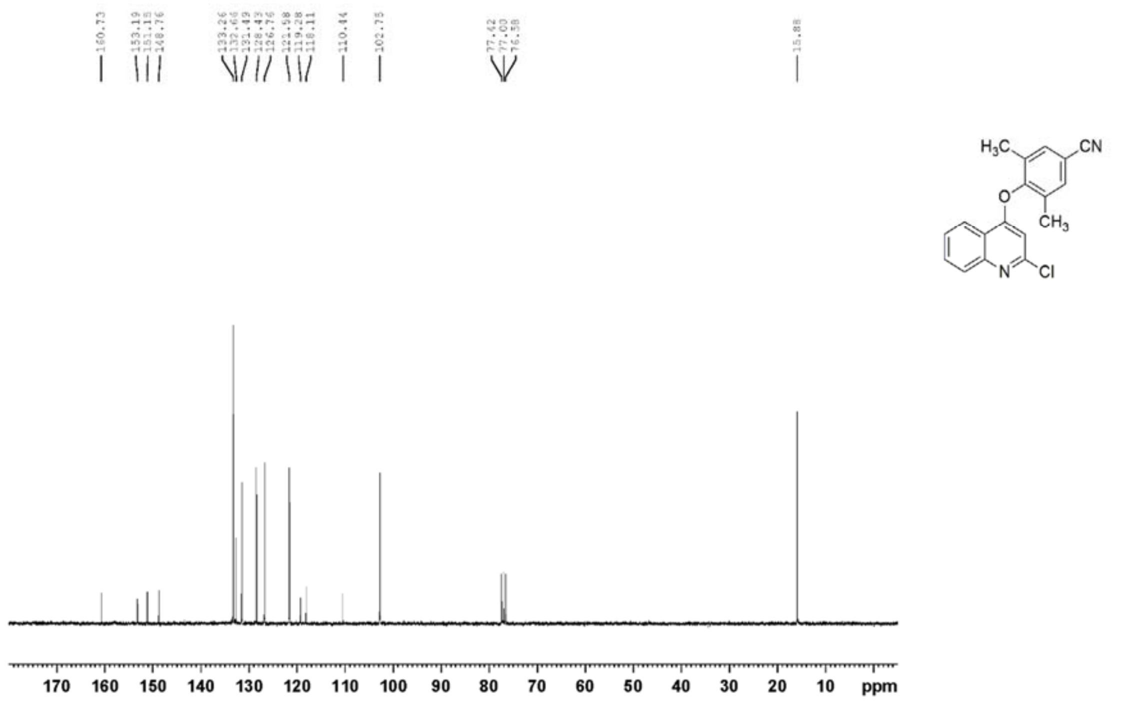

160.73
153.19
151.15
148.76
133.26
132.66
131.49
128.43
126.76
121.58
119.28
118.11
110.44
102.75
77.42
77.00
76.58
15.88
H_3C
CN
O
CH_3
N
Cl
170 160 150 140 130 120 110 100 90 80 70 60 50 40 30 20 10 ppm

2-(4′-formylphenoxy)-4-chloroquinoline (3a)

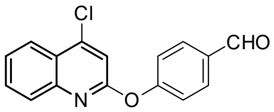

Analysis:

Mp. 121.7-122.5 °C

^{1}H-NMR (300 MHz, $CDCl_3$): 7.26 (s, 1H, ArH-3), 7.41 (dd, 2H, J = 8.6, 1.8 Hz, ArH-2′, 6′), 7.53 (td, 1H, J = 7.6, 1.2 Hz, ArH-6), 7.68 (td, 1H, J = 7.7, 1.4 Hz, ArH-7), 7.79 (dd, 1H, J = 8.4, 0.5 Hz, ArH-8), 7.95 (dt, 2H, J = 8.6, 2.4 Hz, ArH-3′, 5′), 8.16 (dd, 1H, J = 8.3, 1.0 Hz, ArH-5), and 10.0 (s, 1H, CHO)

^{13}C-NMR (75 MHz, $CDCl_3$): 112.9, 121.6, 124.0, 124.2, 126.1, 128.2, 131.0, 131.4, 133.1, 145.2, 146.4, 158.5, 159.9 and 190.9

HRMS (+ESI): $C_{16}H_{11}ClNO_2$ $[M+H]^+$ requires 284.0478, found 284.0468

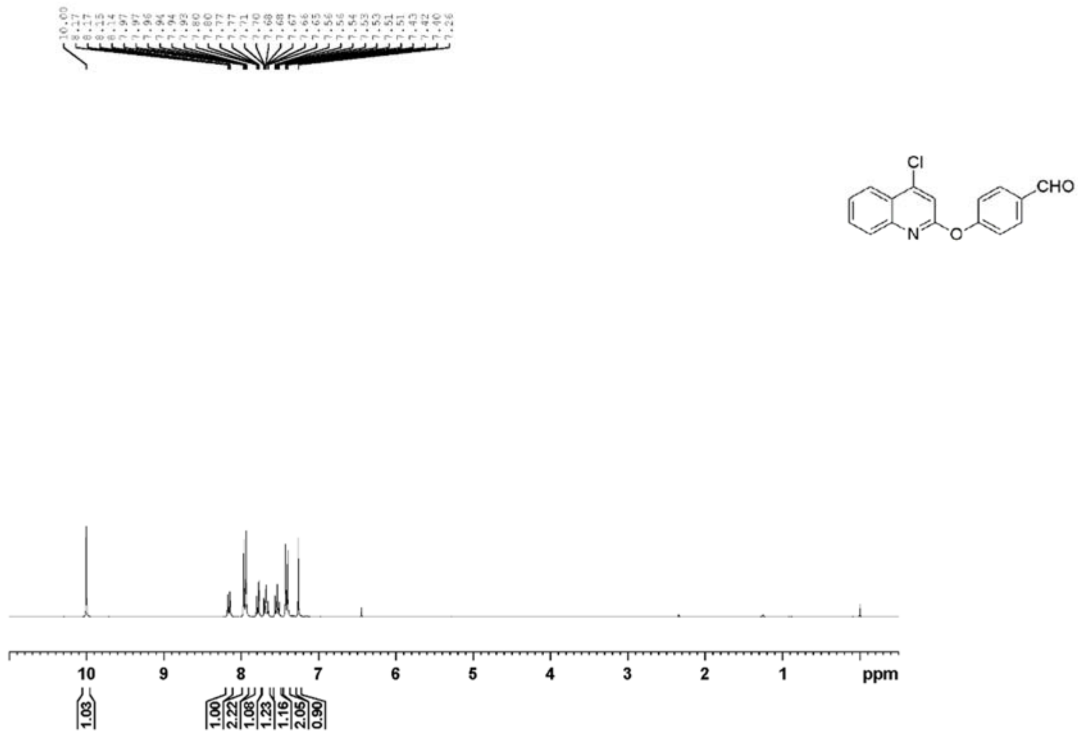

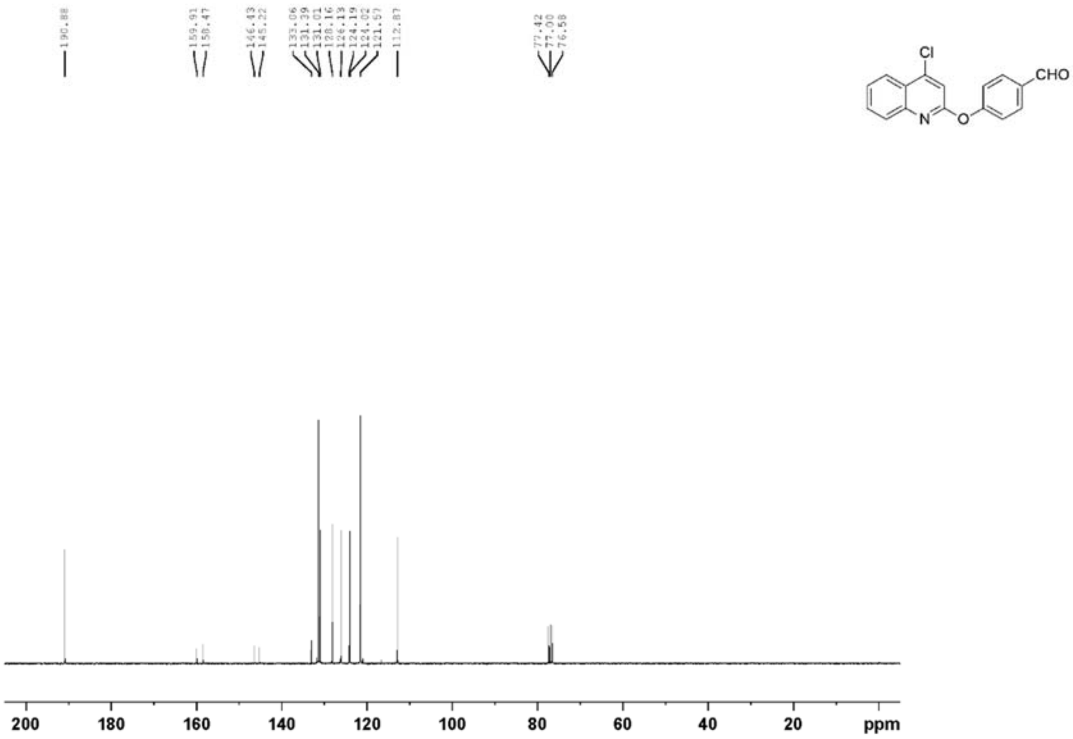
190.88
159.91
158.47
146.43
145.22
133.06
131.39
131.01
128.16
126.13
124.19
124.02
121.57
112.87
77.42
77.00
76.58
Cl
N
O
CHO
200
180
160
140
120
100
80
60
40
20
ppm

2-(2′,6′-Dimethyl-4′-formylphenoxy)-4-chloroquinoline (3b)

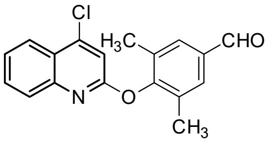

Analysis:

Mp. 113.7-114.2 °C

^{1}H-NMR (300 MHz, $CDCl_3$): 2.21 (s, 6H, $ArCH_3$-2′, 6′), 7.27 (d, 1H, J = 5.8 Hz, ArH-3), 7.50 (td, 1H, J = 7.4, 1.7 Hz, ArH-6), 7.60-7.70 (m, 4H, ArH-7, 8, 3′, 5′), 8.15 (dd, 1H, J = 8.5, 0.8 Hz, ArH-5), and 9.99 (s, 1H, CHO)

^{13}C-NMR (75 MHz, $CDCl_3$): 16.7, 111.5, 123.8, 124.0, 125.6, 128.2, 130.3, 130.9, 132.5, 133.7, 145.2, 146.9, 155.3, 159.7 and 191.7

HRMS (+ESI): $C_{18}H_{15}ClNO_2$ $[M+H]^+$ requires 312.0791, found 312.0777

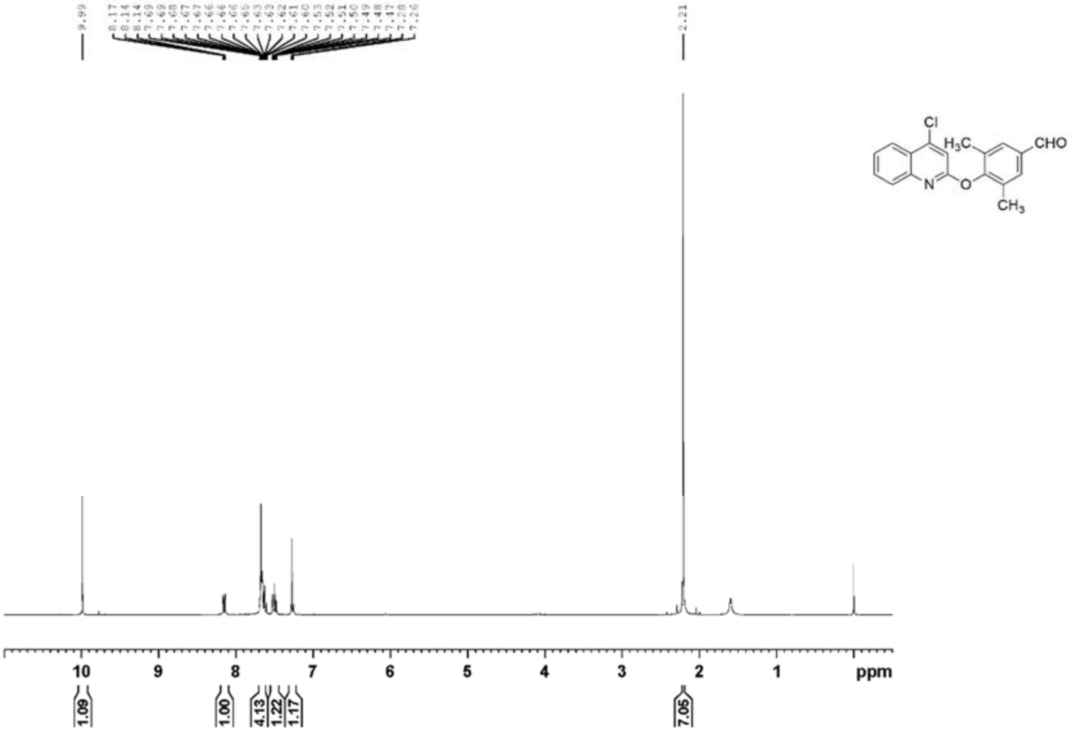

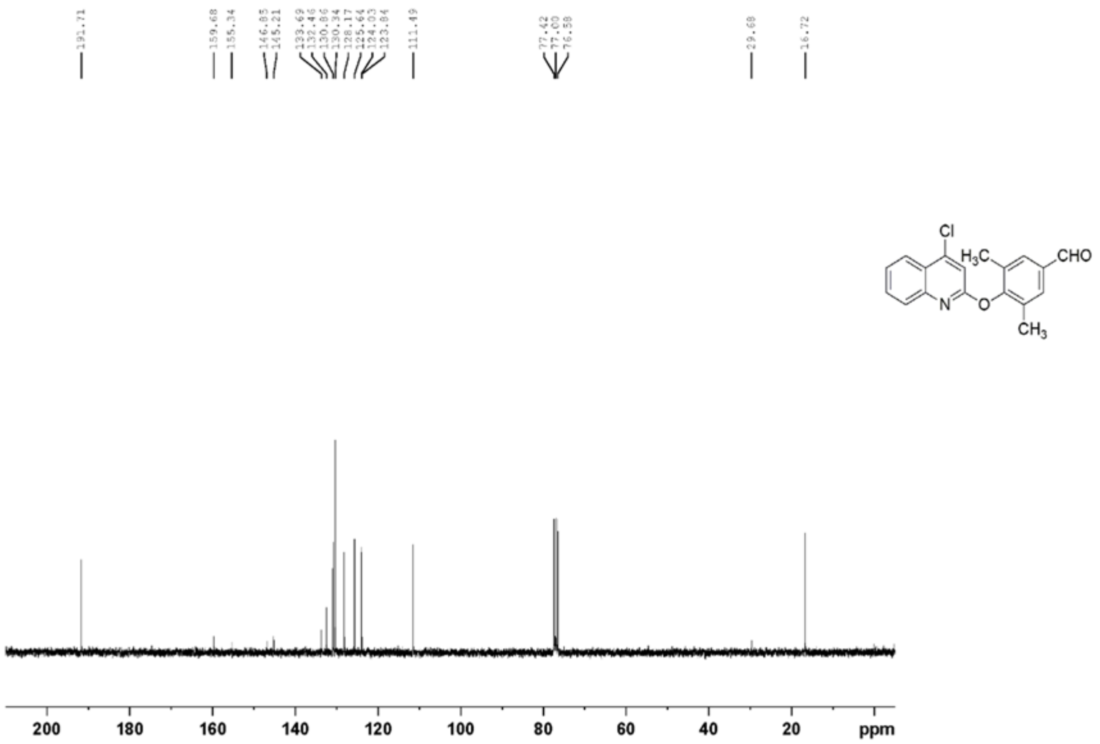
191.71
159.68
155.34
146.85
145.21
133.69
132.46
130.86
130.34
128.17
125.64
124.03
123.84
111.49
77.42
77.00
76.58
29.68
16.72
Cl
H3C
CHO
N
O
CH3
200
180
160
140
120
100
80
60
40
20
ppm

2-(4′-cyanophenoxy)-4-chloroquinoline (3c)

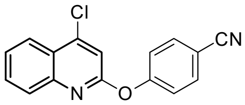

Analysis:

Mp. 147.0-148 °C

^{1}H-NMR (300 MHz, $CDCl_3$): 7.27 (d, 1H, J = 5.7 Hz, ArH-3), 7.39 (dt, 2H, J = 8.4, 0.8 Hz, ArH-2′, 6′), 7.56 (td, 1H, J = 7.3, 1.3 Hz, ArH-6), 7.65-7.82 (m, 4H, ArH-7, 8, 3′, 5′), and 8.18 (dd, 1H, J = 8.1, 0.9 Hz, ArH-5)

^{13}C-NMR (75 MHz, $CDCl_3$): 108.4, 112.8, 118.6, 122.1, 124.1, 124.3, 126.3, 128.2, 131.1, 133.8, 145.4, 146.4, 156.9 and 159.7

HRMS (+ESI): $C_{18}H_{15}ClNO_2$ $[M+H]^+$ requires 281.0482, found 281.0475

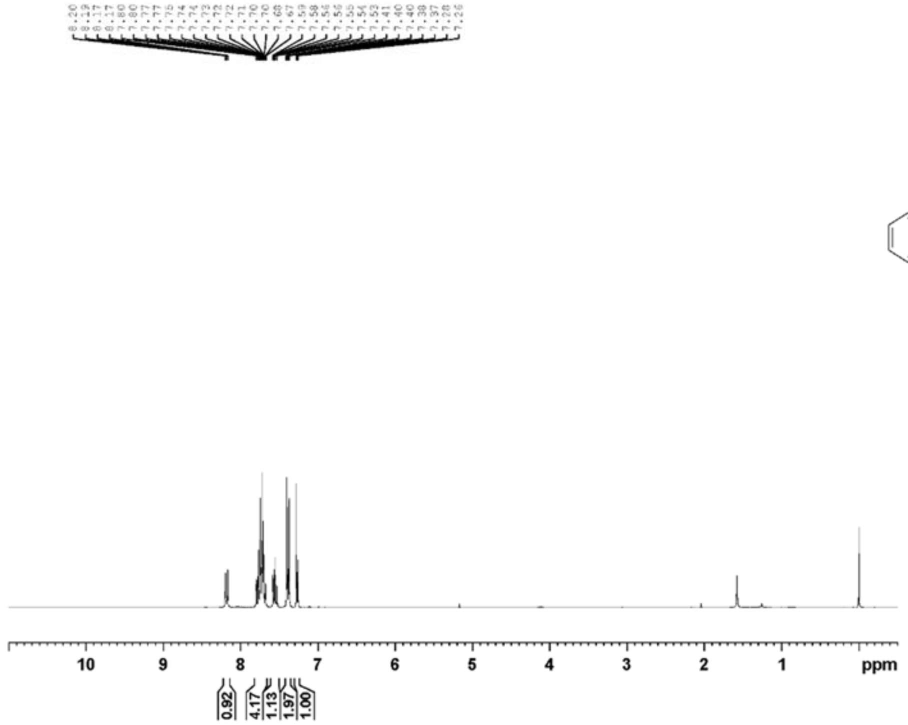

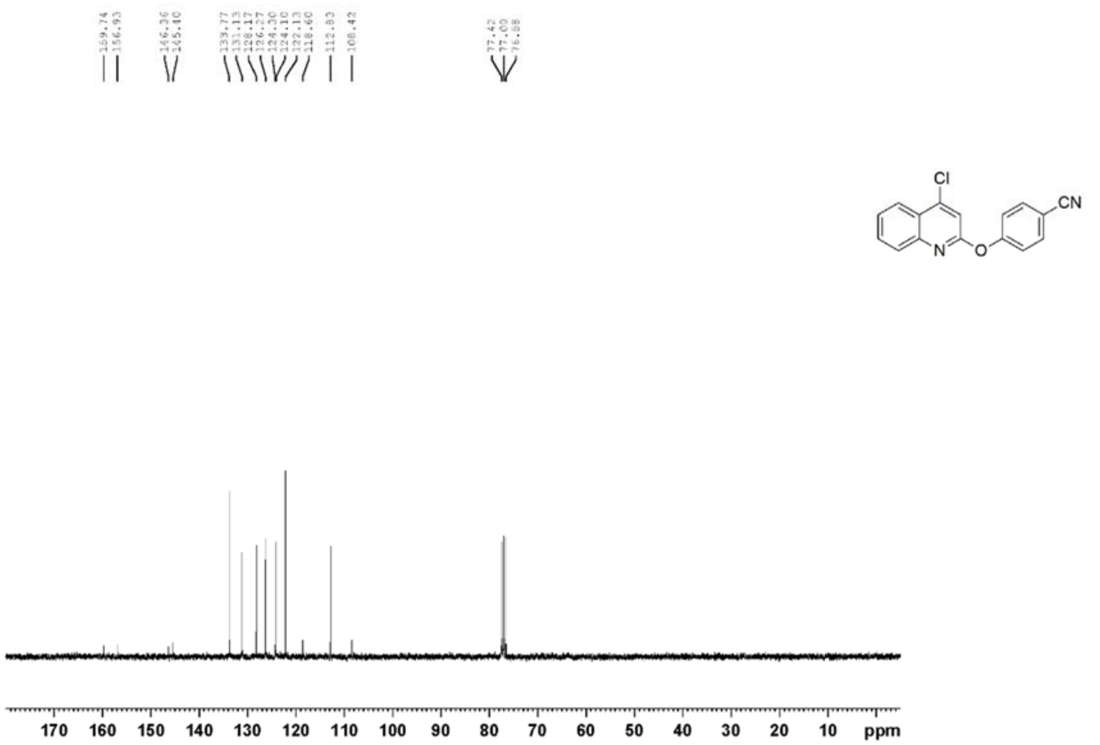
159.74
156.93
146.36
145.40
133.77
131.13
128.17
126.27
124.20
124.10
122.13
118.60
112.83
108.42
77.42
77.00
76.58
Cl
N
O
CN
170 160 150 140 130 120 110 100 90 80 70 60 50 40 30 20 10 ppm

2-(2′,6′-Dimethyl-4′-cyanophenoxy)-4-chloroquinoline (3d)

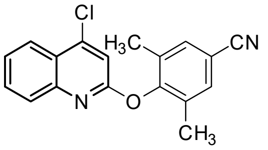

Analysis:

Mp. 152.2-153.0 °C

^{1}H-NMR (300 MHz, $CDCl_3$): 2.16 (s, 6H, $ArCH_3$-2′, 6′), 7.29 (s, 1H, ArH-3), 7.44 (s, 2H, ArH-3′, 5′), 7.51 (td, 1H, J = 7.3, 2.2 Hz, ArH-6), 7.60-7.69 (m, 2H, ArH-7, 8) and 8.16 (d, 1H, J = 8.2 Hz, ArH-5)

^{13}C-NMR (75 MHz, $CDCl_3$): 16.6, 109.1, 111.5, 118.9, 123.9, 124.0, 125.7, 128.1, 130.9, 132.5, 133.0, 145.3, 146.7, 153.9 and 159.4

HRMS (+ESI): $C_{18}H_{14}ClN_2O$ $[M+H]^+$ requires 309.0795, found 309.0787

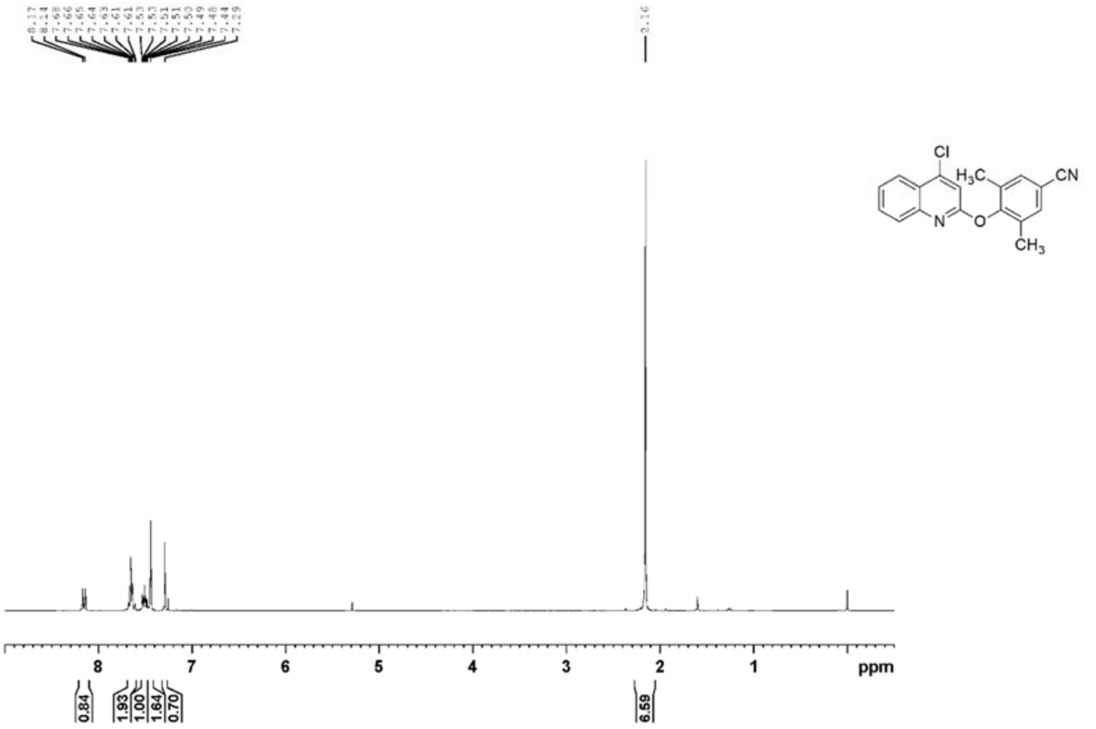

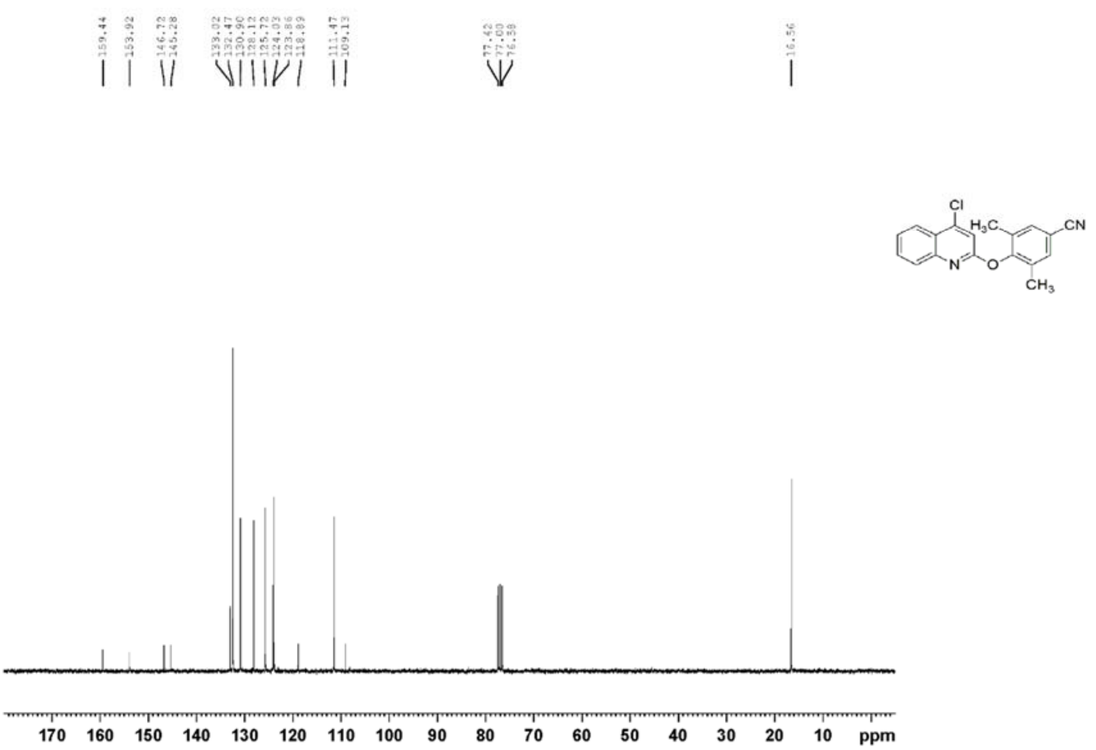

2,4-di-(4′-formylphenoxy)-quinoline (4a)

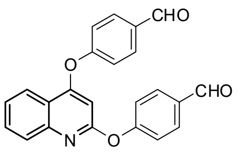

Analysis:

mp: 164.9-165.5 °C

^{1}H-NMR (300 MHz, $CDCl_3$): 6.43 (s, 1H, ArH-3), 7.36-7.43 (m, 4H, ArH-2′, 6′, 2″, 6″), 7.51 (td, 1H, J = 7.6, 1.3 Hz, ArH-6), 7.71 (td, 1H, J = 8.1, 1.5 Hz, ArH-7), 7.80 (dd, 1H, J = 8.4, 0.5 Hz, ArH-8), 7.94 (ddd, 2H, J = 8.7, 2.0 Hz, ArH-3′, 5′), 8.03 (ddd, 2H, J = 8.7, 2.0 Hz, ArH-3″, 5″), 8.22 (dd, 1H, J = 8.3, 0.9 Hz, ArH-5), 10.00 (s, 1H, CHO) and 10.04 (s, 1H, CHO)

^{13}C-NMR (75 MHz, $CDCl_3$): 97.2, 119.7, 120.9, 121.5, 121.7, 125.3, 127.9, 131.1, 131.4, 132.2, 132.9, 133.7, 147.3, 158.7, 159.5, 161.3, 163.2, 190.5 and 191.0

HRMS (+ESI): $C_{23}H_{16}NO_4$ $[M+H]^+$ requires 370.1079, found 370.1088.

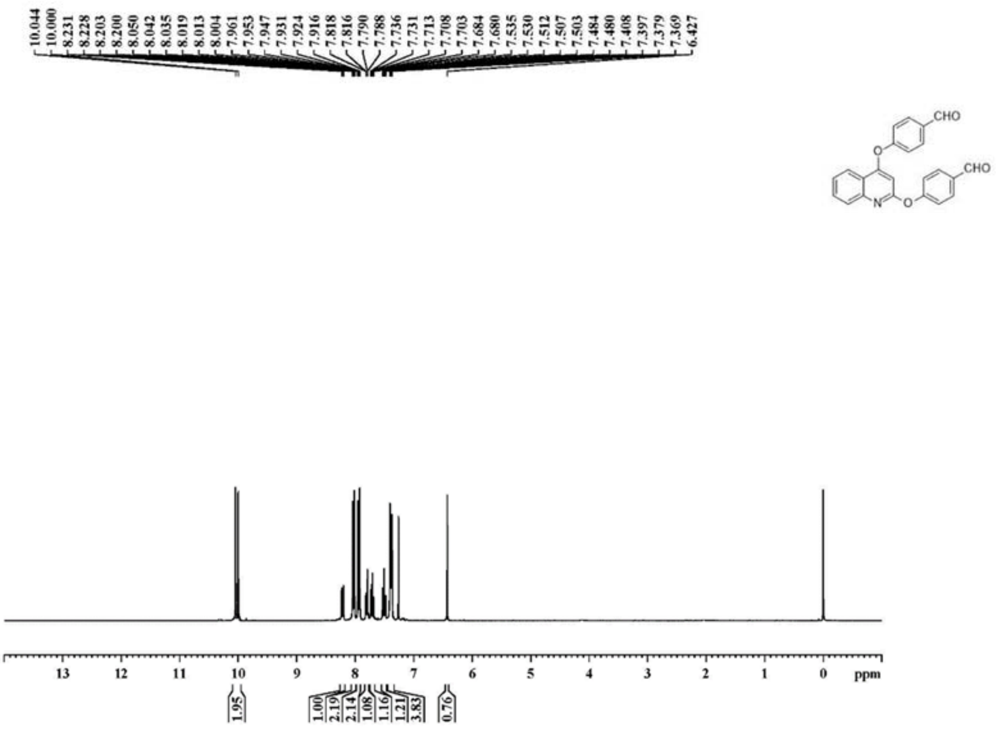

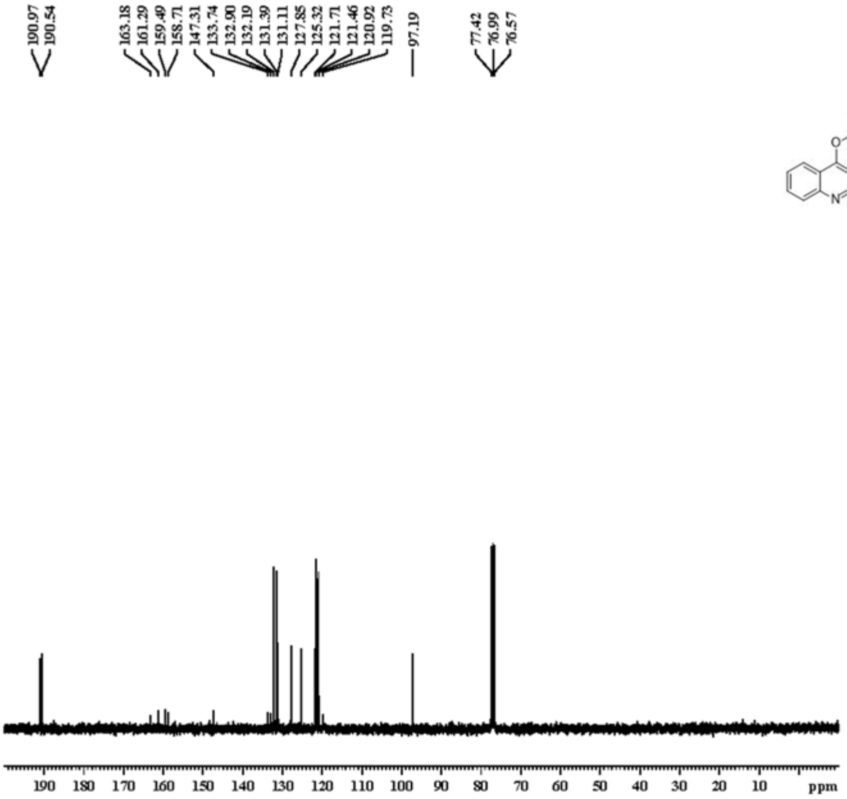
190.97
190.54
163.18
161.29
159.49
158.71
147.31
133.74
132.90
132.19
131.39
131.11
127.85
125.32
121.71
121.46
120.92
119.73
97.19
77.42
76.99
76.57
190 180 170 160 150 140 130 120 110 100 90 80 70 60 50 40 30 20 10 ppm

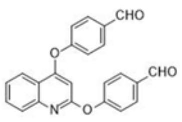
CHO
O
CHO
N
O

2,4-di-(2′,6′-Dimethyl-4′-formylphenoxy)-quinoline (4b)

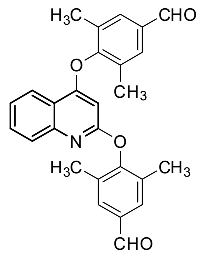

Analysis:

mp. 120.5-121.0 °C

^{1}H-NMR (300 MHz, $CDCl_3$): 2.16 (s, 6H, $ArCH_3$-2′, 6′), 2.29 (s, 6H, $ArCH_3$-2″, 6″), 5.99 (s, 1H, ArH-3), 7.46-7.51 (m, 1H, ArH-6), 7.63-7.66 (m, 4H, ArH-3′, 5′, 7, 8), 7.76 (s, 2H, ArH-3″, 5″), 8.35 (d, 1H, J = 8.1 Hz, ArH-5), 9.95 (s, 1H, CHO) and 10.02 (s, 1H, CHO)

^{13}C-NMR (75 MHz, $CDCl_3$): 16.1, 16.7, 91.7, 118.5, 121.6, 124.5, 127.7, 130.2, 130.7, 130.9, 132.2, 132.4, 133.4, 134.2, 147.5, 155.0, 155.6, 161.3, 162.1, 191.3 and 191.7

HRMS (+ESI): $C_{27}H_{24}NO_4$ $[M+H]^+$ requires 426.1700, found 426.1690.

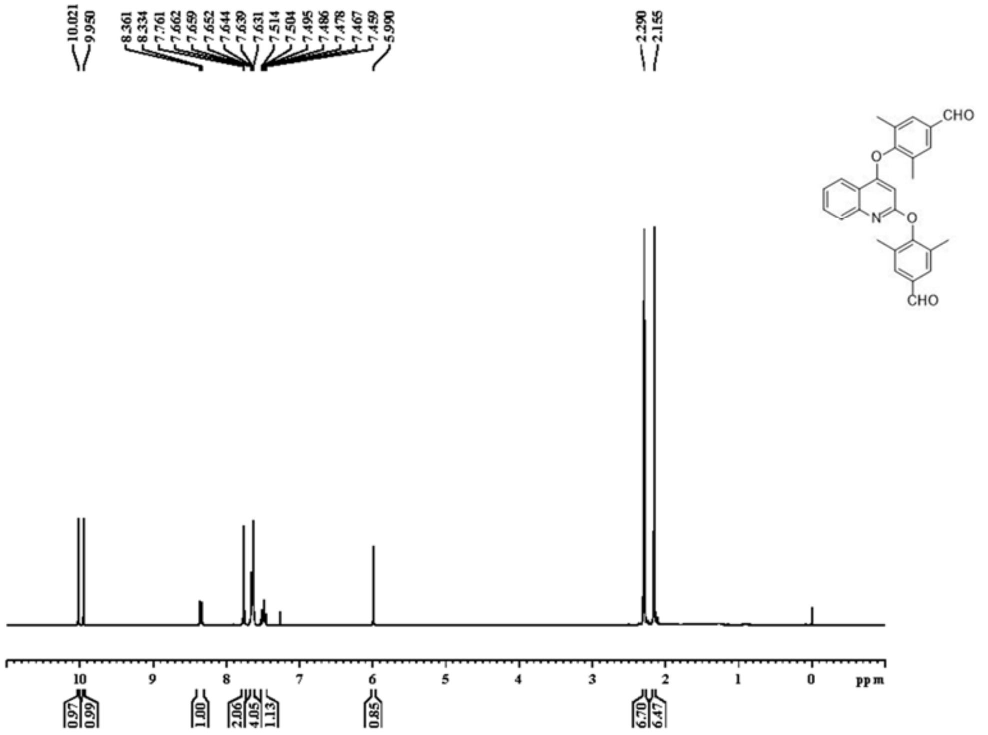

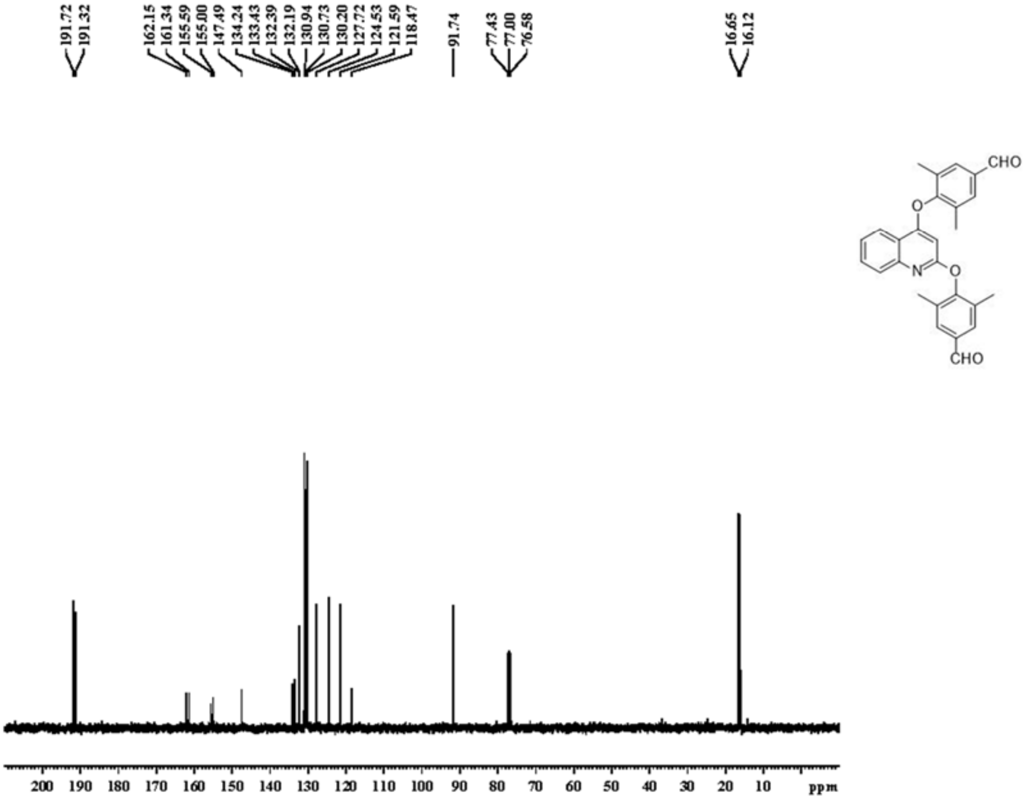
191.72
191.32
162.15
161.34
155.59
155.00
147.49
134.24
133.43
132.39
132.19
130.94
130.73
130.20
127.72
124.53
121.59
118.47
91.74
77.43
77.00
76.58
16.65
16.12
CHO
O
N
O
CHO
200 190 180 170 160 150 140 130 120 110 100 90 80 70 60 50 40 30 20 10 ppm

2,4-di-(4′-cyanophenoxy)-quinoline (4c)

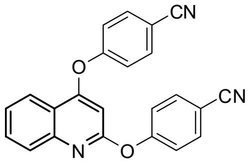

Analysis:

Mp. 220.9-221.5 °C

^{1}H-NMR (300 MHz, $CDCl_3$): 6.40 (s, 1H, ArH-3), 7.30-7.40 (m, 4H, ArH-2′, 6′, 2″, 6″), 7.51 (td, 1H, J = 7.5, 1.3 Hz, ArH-6), 7.66-7.75 (m, 3H, ArH-7, 3′, 5′), 7.75-7.84 (m, 3H, ArH-8, 3″, 5″) and 8.18 (dd, 1H, J = 8.0, 0.7 Hz, ArH-5)

^{13}C-NMR (75 MHz, $CDCl_3$): 97.1, 108.2, 109.4, 118.0, 118.6, 119.6, 121.3, 121.6, 122.0, 125.5, 127.8, 131.3, 133.7, 134.7, 147.2, 157.0, 158.1, 161.0 and 163.0

HRMS (+ESI): $C_{23}H_{14}N_3O_2$ $[M+H]^+$ requires 364.1086, found 364.1067

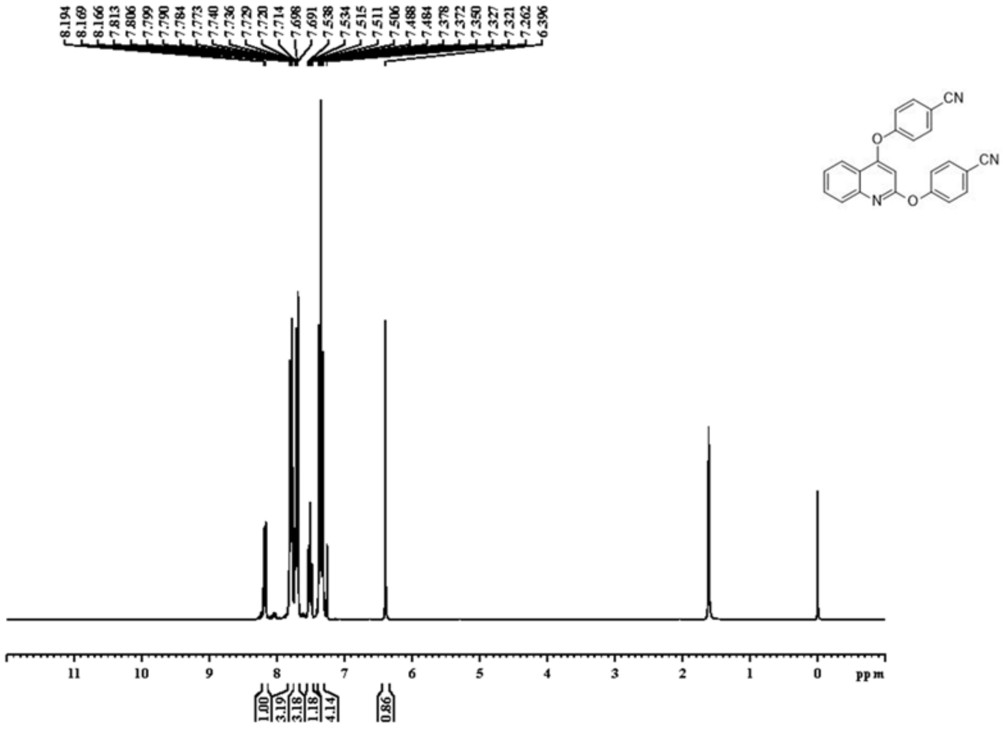

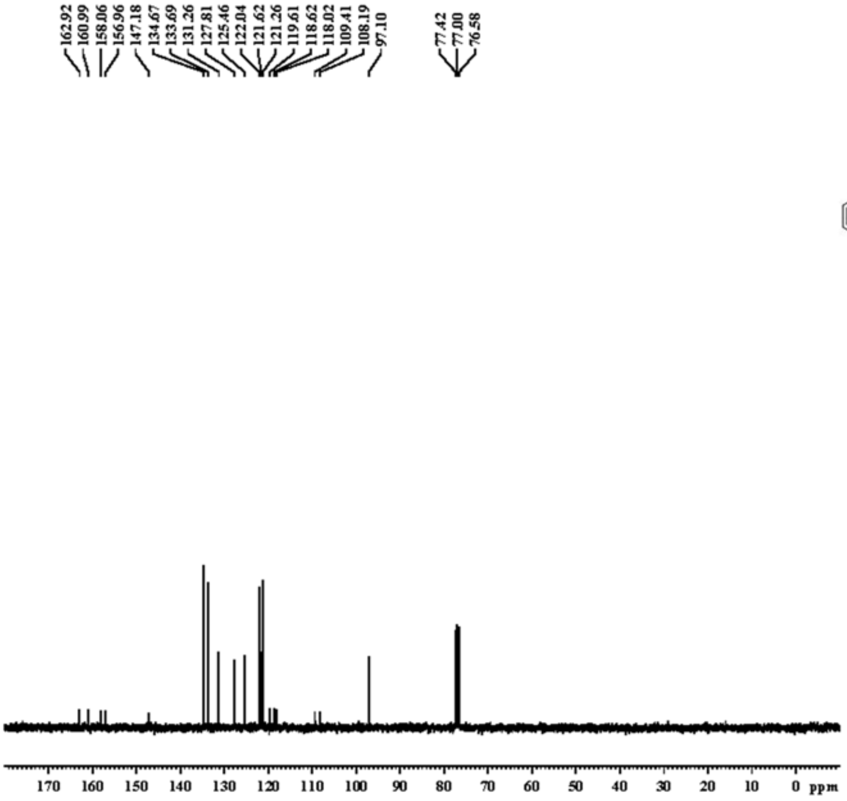
162.92
160.99
158.06
156.96
147.18
134.67
133.69
131.26
127.81
125.46
122.04
121.62
121.26
119.61
118.62
118.02
109.41
108.19
97.10
77.42
77.00
76.58
170 160 150 140 130 120 110 100 90 80 70 60 50 40 30 20 10 0 ppm

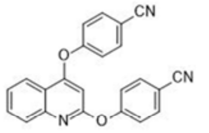
CN
O
CN
N
O

2,4-di-(2′,6′-Dimethyl-4′-cyanophenoxy)-quinoline (4d)

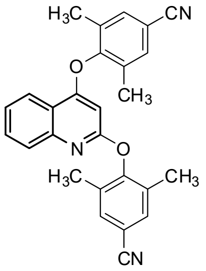

Analysis:

mp: 201.8-202.4 °C

^{1}H-NMR (300 MHz, CDCl3): 2.10 (s, 6H, ArCH3-2′, 6′), 2.24 (s, 6H, ArCH3-2″, 6″), 5.96 (s, 1H, ArH-3), 7.41 (s, 2H, ArH-3′, 5′), 7.46-7.52 (m, 1H, ArH-6), 7.54 (bs, 2H, ArH-3″, 5″), 7.64-7.66 (m, 2H, ArH-7, 8) and 8.32 (d, 1H, J = 8.3 Hz, ArH-5)

^{13}C-NMR (75 MHz, CDCl3): 16.0, 16.5, 91.6, 102.8, 108.9, 110.2, 118.3, 118.4, 119.0, 121.5, 121.6, 124.7, 126.8, 127.8, 128.5, 130.9, 131.6, 132.4, 132.8, 133.0, 133.3, 147.4, 153.7, 154.1, 161.0 and 162.0

HRMS (+ESI): $C_{27}H_{22}N_3O_2$ $[M+H]^+$ requires 420.1706, found 420.1712.

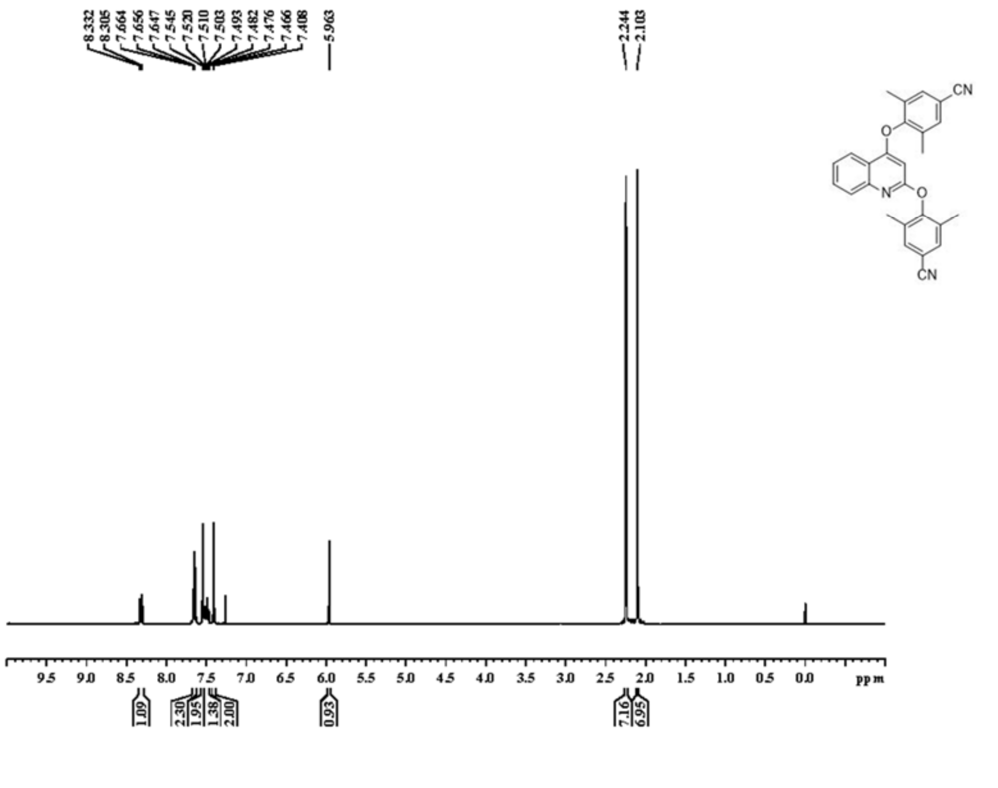

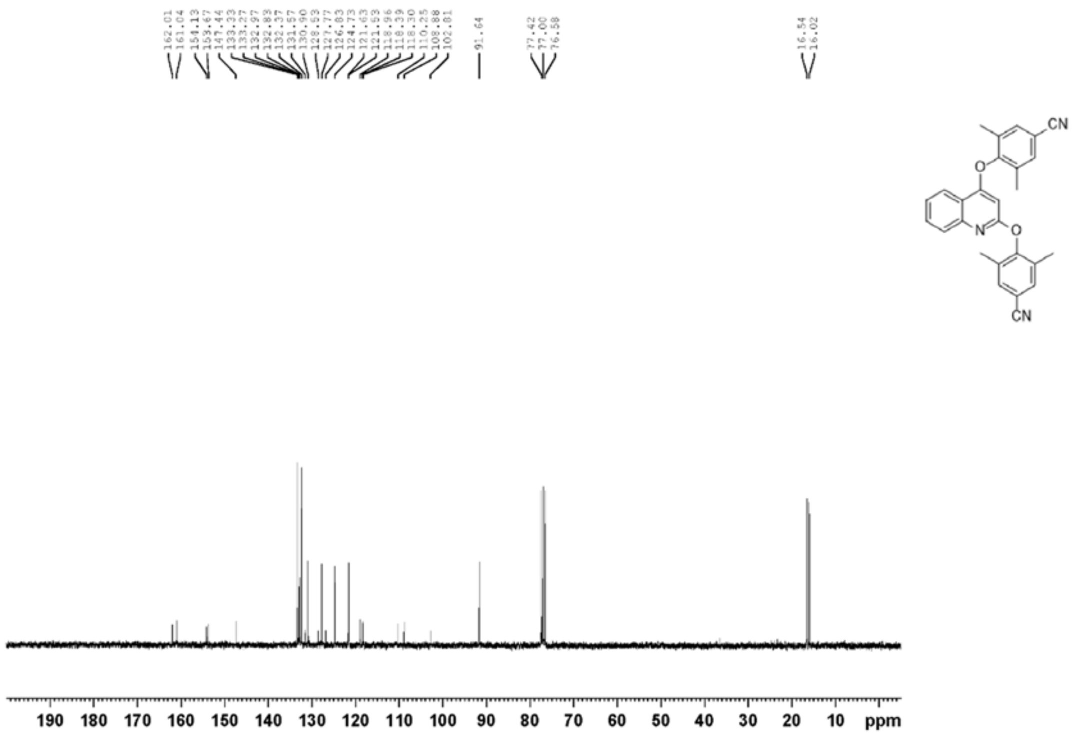

162.01
161.04
154.13
153.67
147.44
133.33
133.27
132.97
132.83
132.37
131.57
130.90
128.53
127.77
126.83
124.73
121.63
121.53
118.96
118.39
118.30
110.25
108.88
102.81
91.64
77.42
77.00
76.58
16.54
16.02
CN
O
N
O
CN
190 180 170 160 150 140 130 120 110 100 90 80 70 60 50 40 30 20 10 ppm

Scheme 2

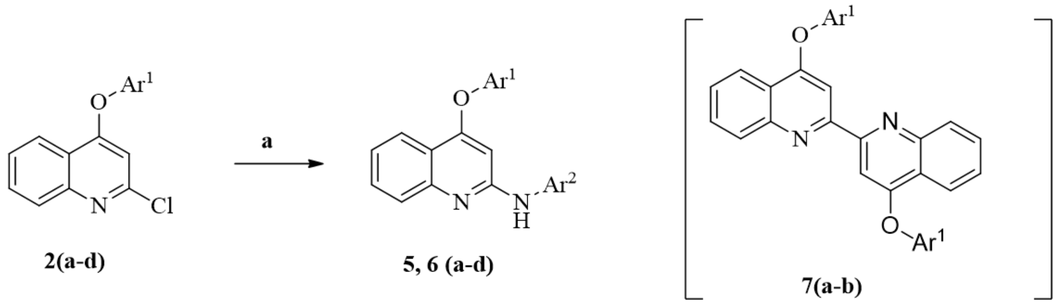

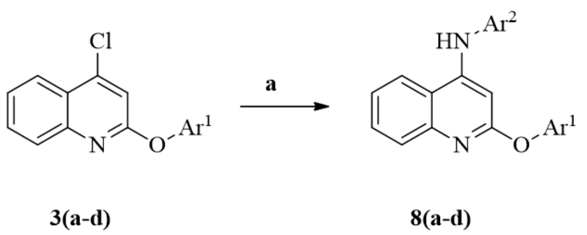

Compounds	Ar^1	Ar^2
5a	4-formylphenoxy	4-cyanophenyl
5b	2,6-dimethyl-4-formylphenoxy	4-cyanophenyl
5c	4-cyanophenoxy	4-cyanophenyl
5d	2,6-dimethyl-4-cyanophenoxy	4-cyanophenyl
6a	4-formylphenoxy	5-cyanopyridinyl
6b	2,6-dimethyl-4-formylphenoxy	5-cyanopyridinyl
6c	4-cyanophenoxy	5-cyanopyridinyl
6d	2,6-dimethyl-4-cyanophenoxy	5-cyanopyridinyl
7a	4,4′-dobis(4″-formyl)phenoxy-2,2′-biquinoline	
7b	4,4′-dobis(4″-formyl-2,6-dimethyl)phenoxy-2,2′-biquinoline	
8a	4-formylphenoxy	4-cyanophenyl
8b	2,6-dimethyl-4-formylphenoxy	4-cyanophenyl
8c	4-cyanophenoxy	4-cyanophenyl
8d	2,6-dimethyl-4-cyanophenoxy	4-cyanophenyl

Synthesis of 2-phenylamino-4-phenoxy-quinoline (**5a**–**5d** and **6a**–**6d**), biquinolines (**7a** and **7b**), 2-phenoxy-4-phenylamine-quinoline (**8a**–**8d**). Reagents and conditions: a. 4-aminobenzonitrile (Ar^2-NH_2, 1.3 eq.) for **5a**–**5d** and **8a**–**8d** or 2-amino-5-cyanopyridine (Ar^2-NH_2) for **6a**–**6d**, Cs_2CO_3 (1.5 eq), $Pd(OAc)_2$ (5 mol.%), SPhos (5 mol.%), DMF, 120 °C, sealed tube.

4-(4′-formylphenoxy)-2-(4′′-cyanophenyl)-aminoquinoline (5a)

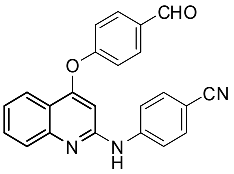

Analysis:

Mp. 267.1-268.0 °C

^{1}H-NMR (300 MHz, DMSO-d6): 6.42 (s, 1H, ArH-3), 7.43 (t, 1H, J = 7.1 Hz, ArH-6), 7.50-7.59 (m, 2H, ArH-2′′, 6′′), 7.66-7.88 (m, 4H, ArH-3′′, 5′′, 2′, 6′), 8.04-8.15 (m, 5H, ArH-5, 7, 8, 3′, 5′), 9.80 (bs, 1H, NH), and 10.10 (s, 1H, CHO)

^{13}C-NMR (75 MHz, DMSO-d6): 97.8, 102.0, 117.6, 117.9, 119.6, 121.2, 123.6, 126.7, 130.7, 132.1, 133.1, 133.5, 145.4, 148.1, 154.1, 159.1, 160.4 and 191.9

HRMS (+ESI): $C_{23}H_{16}N_3O_2$ $[M+H]^+$ requires 366.1237, found 366.1229

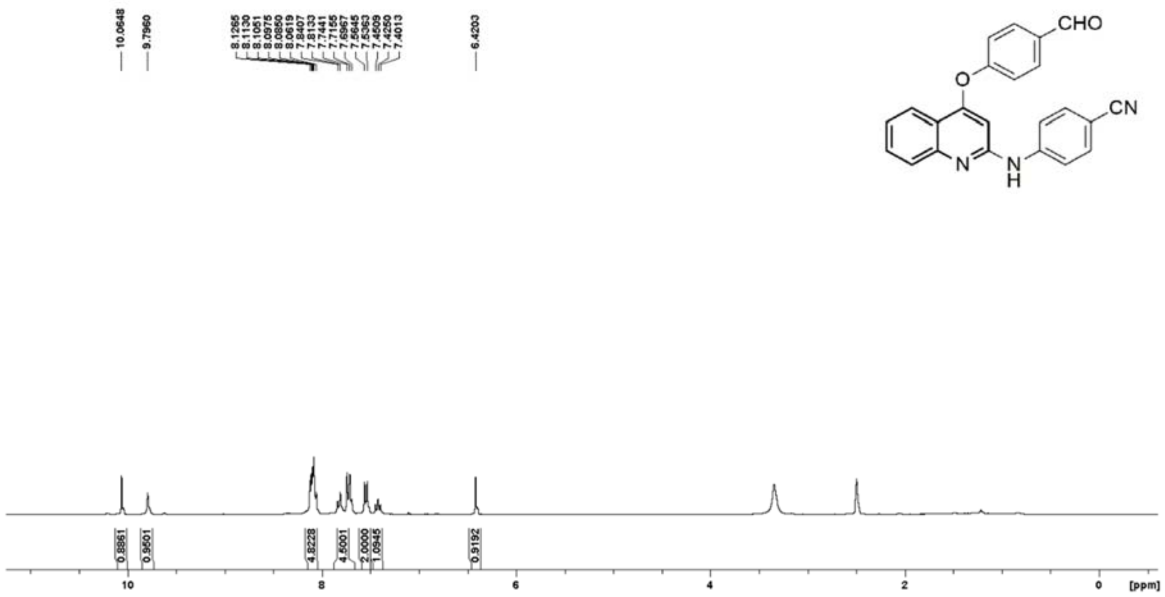

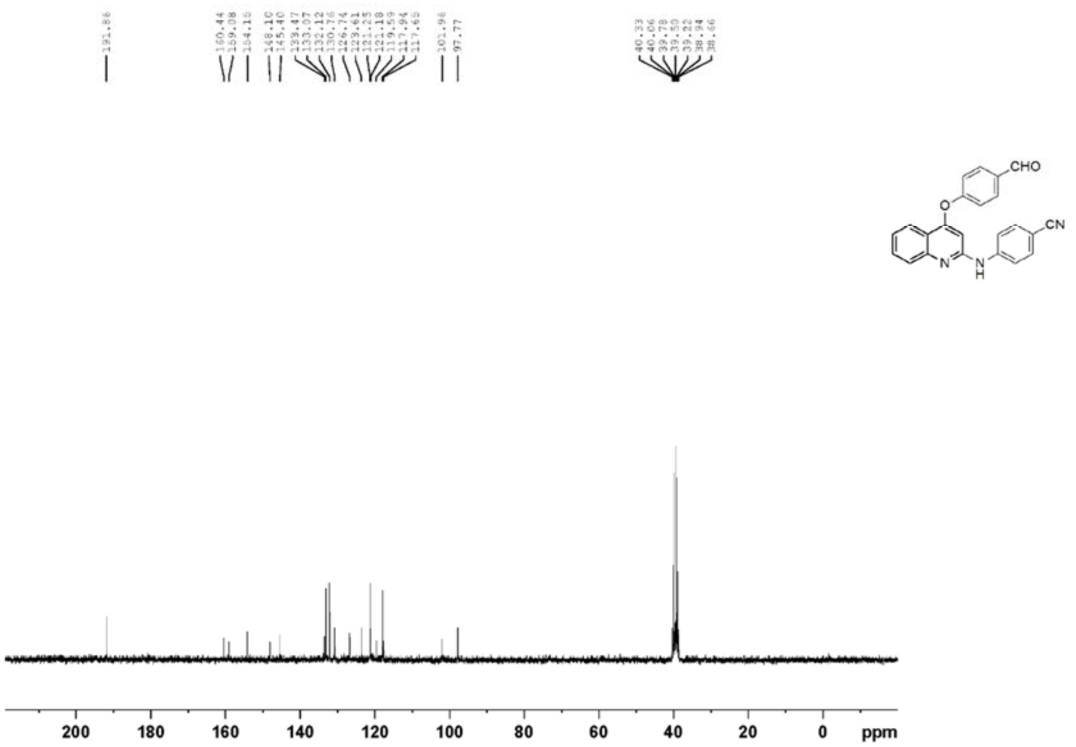

191.86
160.44
159.08
154.15
148.10
145.40
133.47
133.07
132.12
130.76
126.74
123.61
121.25
121.18
119.59
117.94
117.65
101.96
97.77
40.33
40.06
39.78
39.50
39.22
38.94
38.66
CHO
O
CN
N
N
H
200
180
160
140
120
100
80
60
40
20
0
ppm

4-(2′,6′-Dimethyl-4′-formylphenoxy)-2-(4′′-cyanophenyl)-aminoquinoline (5b)

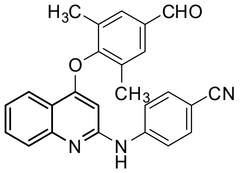

Analysis:

Mp. 291.3-292.2 °C

^{1}H-NMR (300 MHz, $CDCl_3$): 2.26 (s, 6H, $ArCH_3$-2′, 6′), 5.73 (s, 1H, ArH-3), 6.95 (bs, 1H, NH), 7.46 (td, 1H, J = 7.6, 1.0 Hz, ArH-6), 7.56 (m, 2H, ArH-2′′, 6′′), 7.70 (s, 2H, ArH-3′, 5′), 7.74 (m, 1H, ArH-7), 7.87 (m, 2H, ArH-3′′, 5′′), 7.91 (d, 1H, J = 8.6 Hz, ArH-8), 8.30 (dd, 1H, J = 8.2, 1.1 Hz, ArH-5), and 9.96 (s, 1H, CHO)

^{13}C-NMR (75 MHz, $CDCl_3$): 16.1, 93.1, 104.0, 117.6, 118.2, 119.5, 121.5, 123.9, 127.3, 130.9, 132.5, 133.2, 134.1, 144.6, 148.7, 153.5, 155.2, 160.7, and 191.4

HRMS (+ESI): $C_{25}H_{20}N_3O_2$ $[M+H]^+$ requires 394.1550, found 394.1559

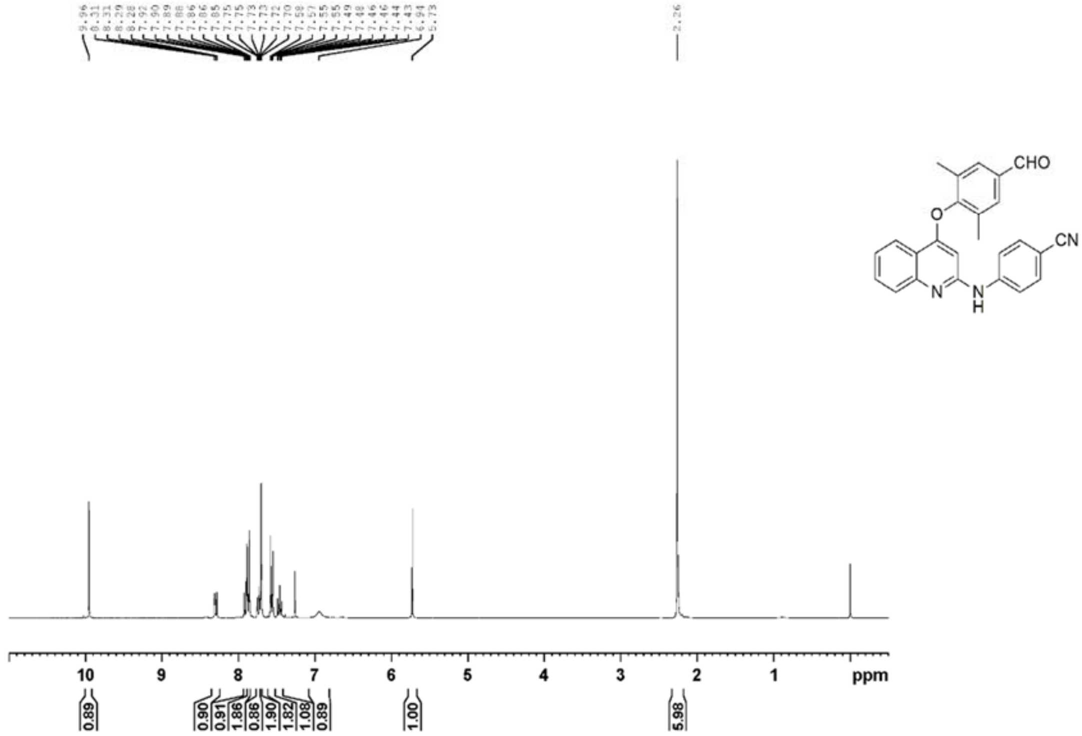

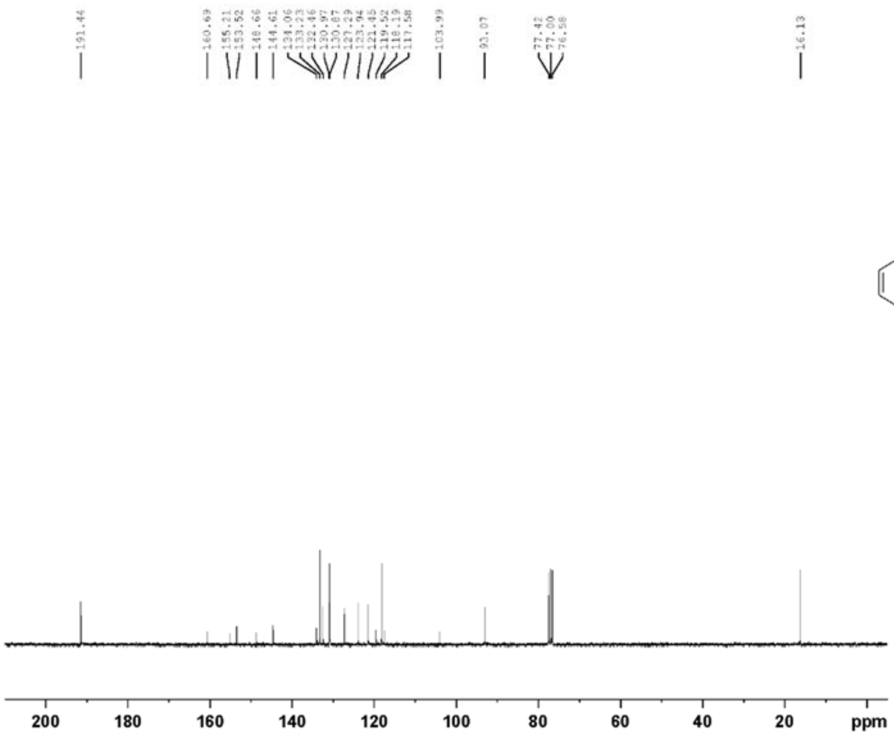
191.44
160.69
155.21
153.52
148.66
144.61
134.06
133.23
132.46
130.97
130.87
127.29
123.94
121.45
119.52
118.19
117.58
103.99
93.07
77.42
77.00
76.58
16.13
200
180
160
140
120
100
80
60
40
20
ppm

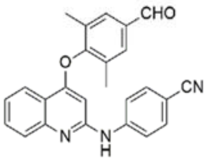
CHO
O
CN
N
N
H

4-(4′-cyanophenoxy)-2-(4′′-cyanophenyl)-aminoquinoline (5c)

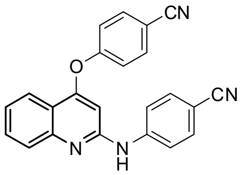

Analysis:

Mp. 278.5-278.7 °C

^{1}H-NMR (300 MHz, $CDCl_3$): 6.59 (s, 1H, ArH-3), 7.17-7.31 (m, 4H, ArH-2′′, 6′′, 2′, 6′), 7.47-7.54 (m, 1H, ArH-6), 7.58-7.80 (m, 7H, ArH-7, 8, 3′′, 5′′, 3′, 5′, NH) and 8.08 (d, 1H, J = 7.9 Hz, ArH-5)

^{13}C-NMR (75 MHz, $CDCl_3$): 103.9, 108.5, 118.1, 119.8, 119.9, 121.6, 125.9, 126.9, 128.0, 131.1, 133.2, 134.4, 147.8, 148.7, 156.0, 158.7, and 160.2

HRMS (+ESI): $C_{23}H_{15}N_4O$ $[M+H]^+$ requires 363.1240, found 363.1232

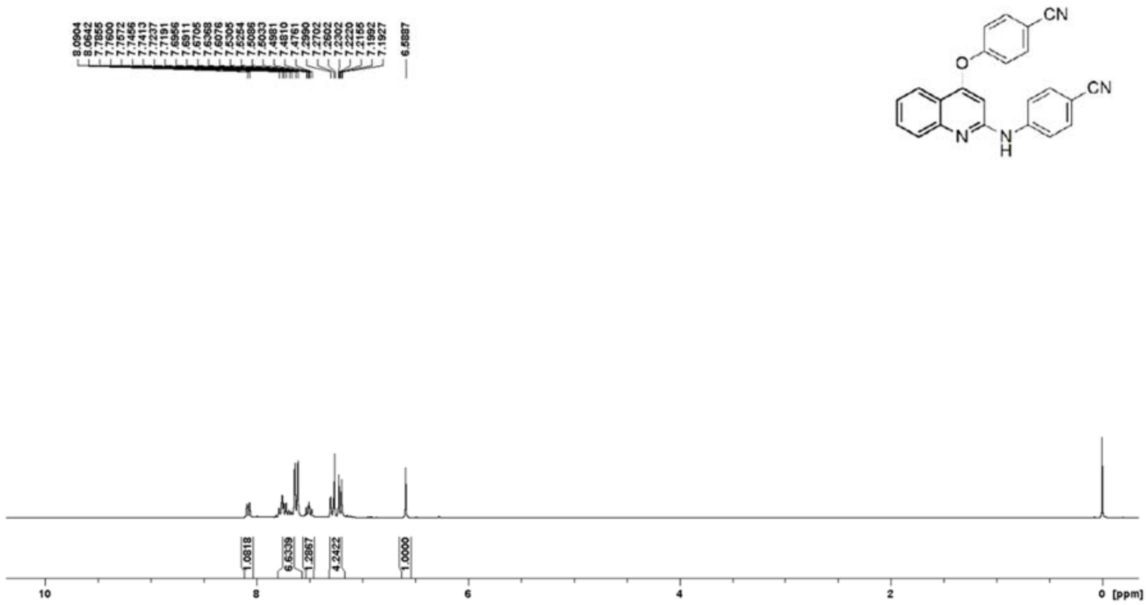

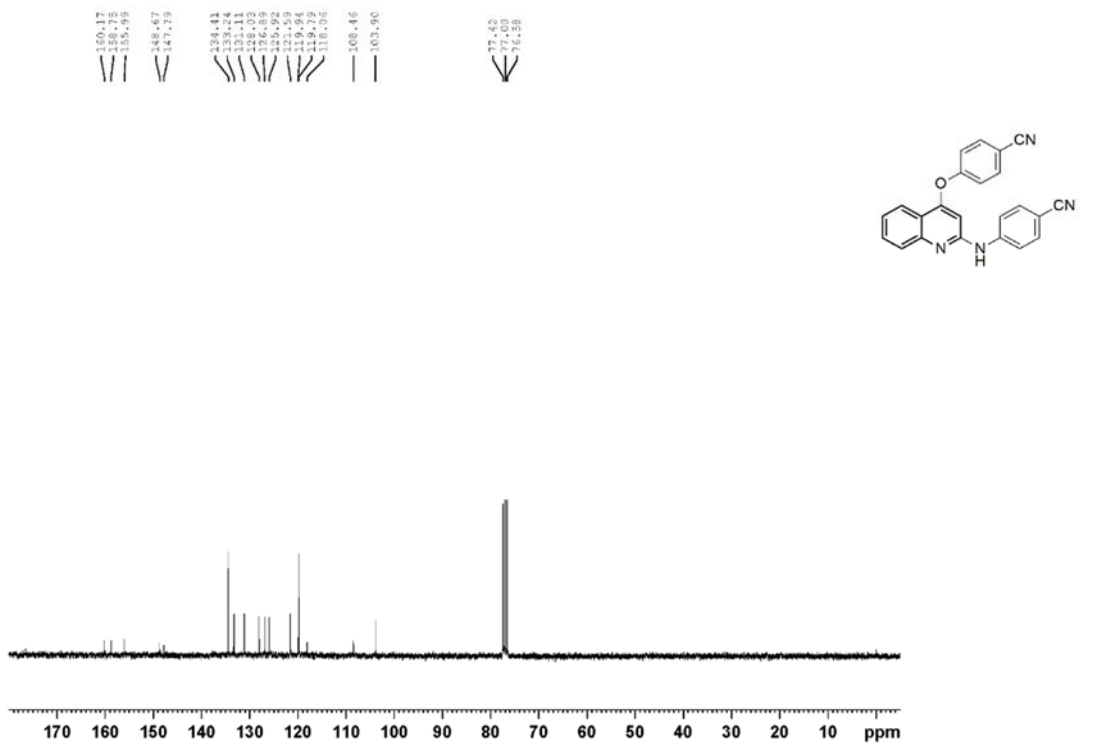

160.17
158.75
155.99
148.67
147.79
134.41
133.24
131.11
128.03
126.89
125.92
121.59
119.94
119.79
118.06
108.46
103.90
77.42
77.00
76.58
CN
O
CN
N
N
H
170
160
150
140
130
120
110
100
90
80
70
60
50
40
30
20
10
ppm

4-(2′,6′-Dimethyl-4′-cyanophenoxy)-2-(4′′-cyanophenyl)-aminoquinoline (5d)

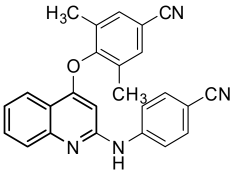

Analysis:

Mp. 256.5-257.0 °C

^{1}H-NMR (300 MHz, $CDCl_3$): 2.23 (s, 6H, $ArCH_3$-2′, 6′), 5.81 (s, 1H, ArH-3), 7.41-7.46 (m, 1H, ArH-6), 7.47 (s, 2H, ArH-3′, 5′), 7.56 (dd, 2H, J = 7.1, 1.8 Hz, ArH-2′′, 6′′), 7.71 (m, 2H, ArH-7, NH), 7.87-7.99 (m, 3H, ArH-8, 3′′, 5′′), and 8.27 (dd, 1H, J = 8.2, 1.1 Hz, ArH-5)

^{13}C-NMR (75 MHz, $CDCl_3$): 16.0, 93.3, 103.6, 109.4, 117.4, 118.2, 119.6, 121.3, 123.8, 127.2, 130.9, 133.0, 133.1, 133.2, 144.8,148.6, 153.8, 154.0, and 160.2

HRMS (+ESI): $C_{25}H_{19}N_4O$ $[M+H]^+$ requires 391.1553, found 391.1548

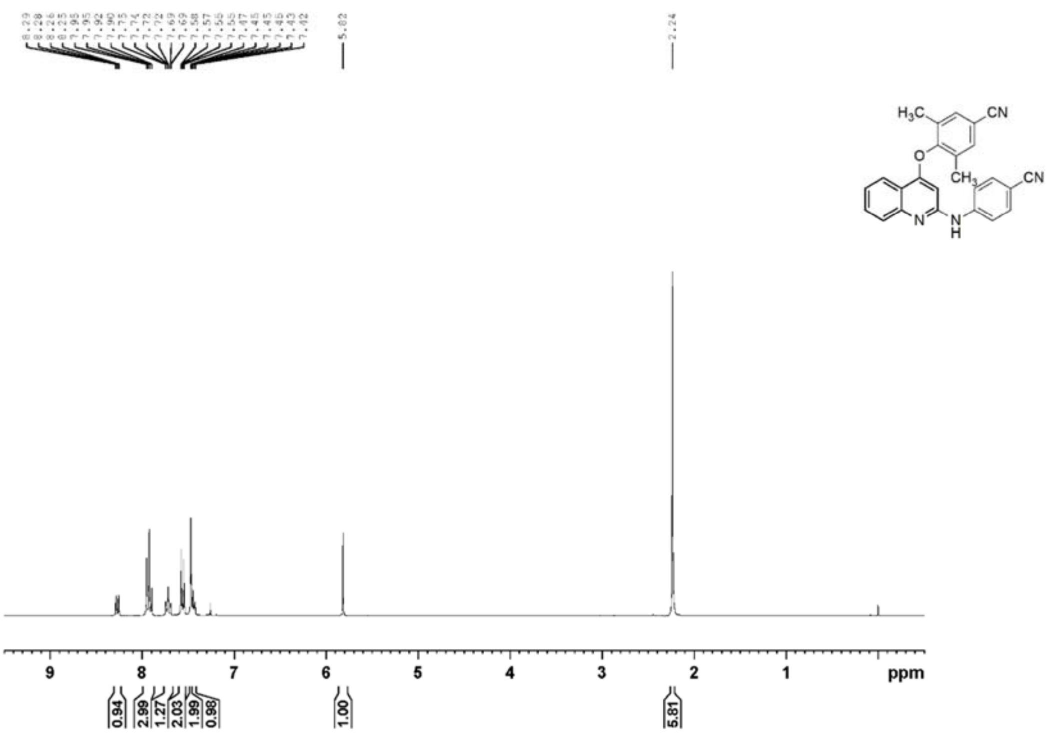

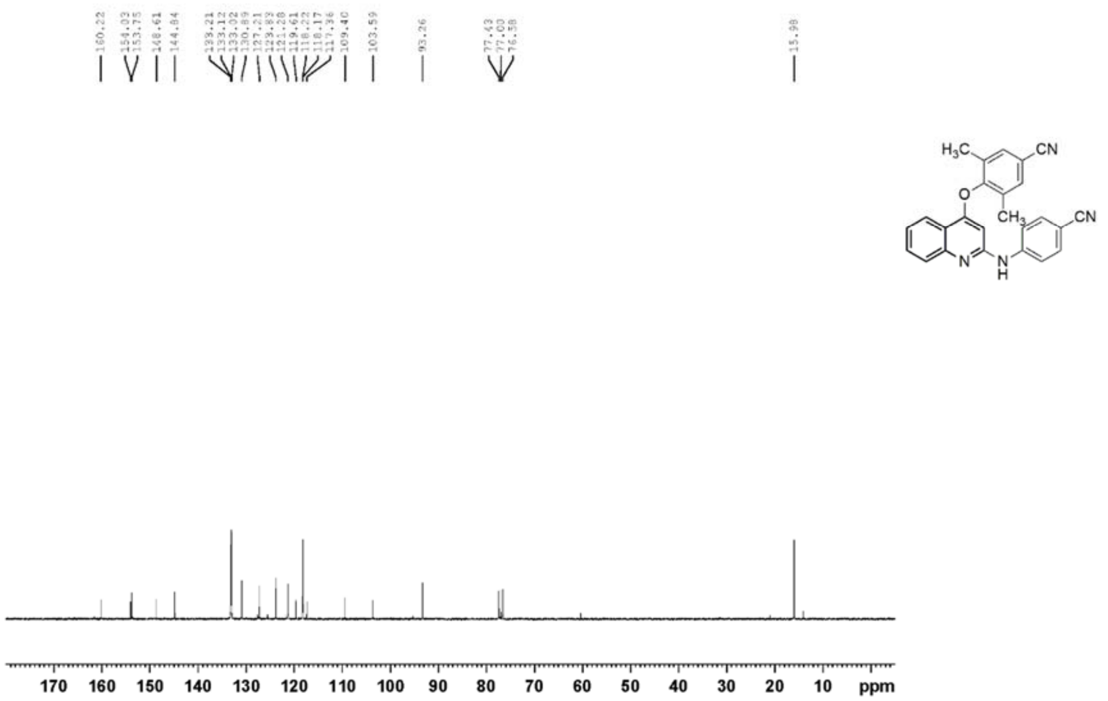
160.22
154.03
153.75
148.61
144.84
133.21
133.12
133.02
130.89
127.21
123.89
121.28
119.61
118.22
118.17
117.36
109.40
103.59
93.26
77.43
77.00
76.58
15.98
H_3C
CN
O
CH_3
CN
N
N
H
170
160
150
140
130
120
110
100
90
80
70
60
50
40
30
20
10
ppm

4-(4'-formylphenoxy)-2-(5''-cyanopyridin-2''ylamino)quinoline (6a)

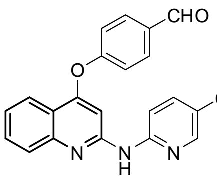

Analysis:

Mp. 248.0-249.0 °C

^{1}H-NMR (300 MHz, $CDCl_3$): 6.77 (s, 1H, ArH-3), 7.34 (d, 2H, J = 8.6 Hz, ArH-2′, 6′), 7.46 (td, 1H, J = 7.6, 0.8 Hz, ArH-3″), 7.74 (td, 1H, J = 7.7, 1.4 Hz, ArH-6), 7.83-7.95 (m, 3H, ArH-7, 8, NH), 7.97-8.06 (m, 2H, ArH-3′, 5′), 8.14 (dd, 1H, J = 8.6, 0.8 Hz, ArH-5), 8.33-8.49 (m, 2H, ArH-4″, 6″) and 10.0 (s, 1H, CHO)

^{13}C-NMR (75 MHz, $CDCl_3$): 98.6, 112.1, 117.5, 120.6, 121.7, 124.7, 127.2, 130.4, 131.3, 132.1, 133.5, 140.8, 151.8, 152.2, 161.7 and 190.5

HRMS (+ESI): $C_{22}H_{15}N_4O_2$ $[M+H]^+$ requires 367.1190, found 367.1176

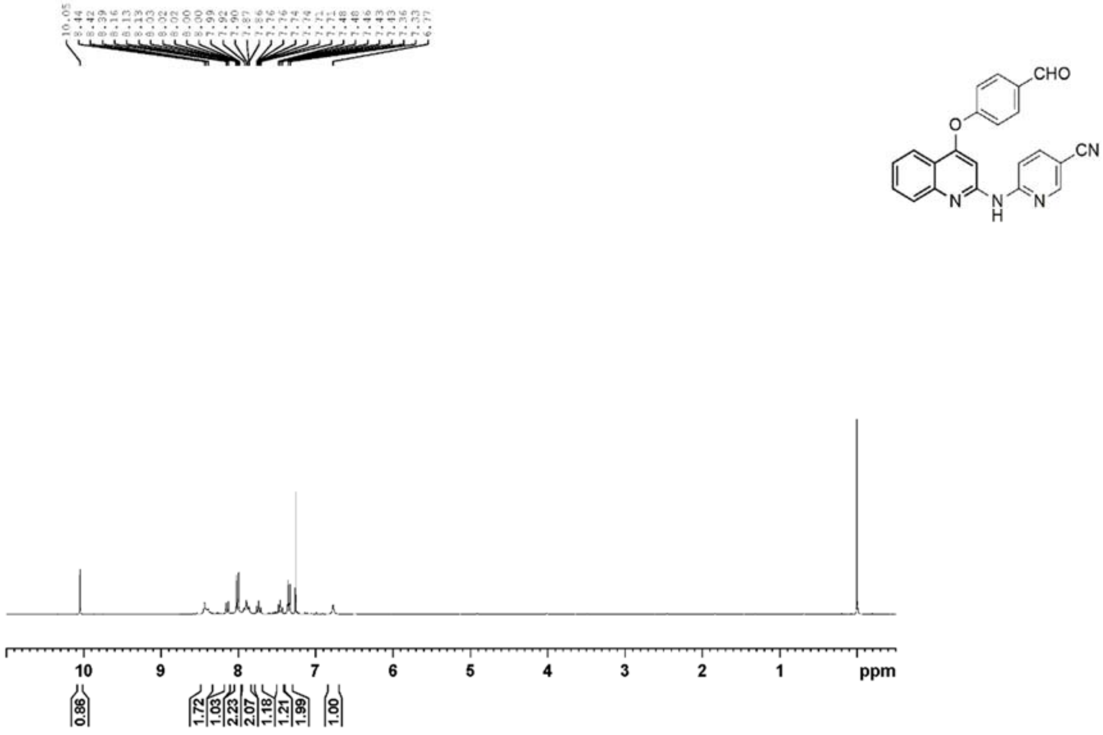

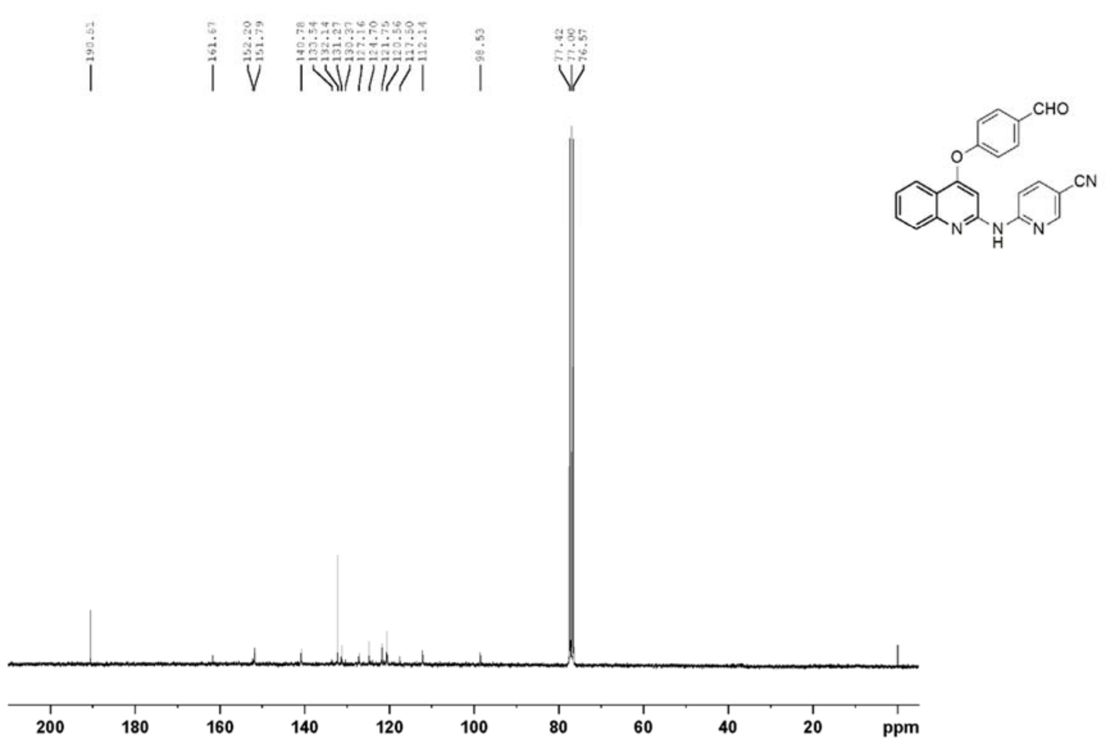
190.51
161.67
152.20
151.79
140.78
133.54
132.14
131.27
130.37
127.16
124.70
121.75
120.56
117.50
112.14
98.53
77.42
77.00
76.57
200
180
160
140
120
100
80
60
40
20
ppm
CHO
O
N
N
H
N
CN

4-(2',6'-Dimethyl-4'-formylphenoxy)-2-(5''-cyanopyridin-2''ylamino)quinoline (6b)

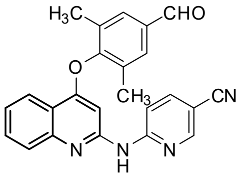

Analysis:

Mp. 275.7-276.5 °C

^{1}H-NMR (300 MHz, DMSO-d6): 2.20 (s, 6H, ArCH3-2′, 6′), 6.58 (s, 1H, ArH-3), 7.52 (t, 1H, J = 7 Hz, ArH-3′′), 7.77 (td, 1H, J = 7.8, 16 Hz, ArH-6), 7.82-7.91 (m, 3H, ArH-7, 3′, 5′), 8.14 (dd, 1H, J = 8.8, 2.3 Hz, ArH-8), 8.29 (d, 1H, J = 7.5 Hz, ArH-5), 8.57 (d, 1H, J = 1.9 Hz, ArH-4′′), 8.65 (d, 1H, J = 8.9 Hz, ArH-6′′), 10.03 (s, 1H, CHO) and 10.37 (s, 1H, NH)

^{13}C-NMR (75 MHz, DMSO-d6): 15.6, 94.8, 97.5, 100.3, 111.8, 117.2, 117.9, 121.3, 124.2, 126.8, 130.7, 130.9, 131.9, 134.1, 141.1, 145.9, 151.9, 153.4, 154.5, 155.9, 159.7 and 160.4

HRMS (+ESI): $C_{24}H_{19}N_4O_2$ $[M+H]^+$ requires 395.1502, found 395.1500

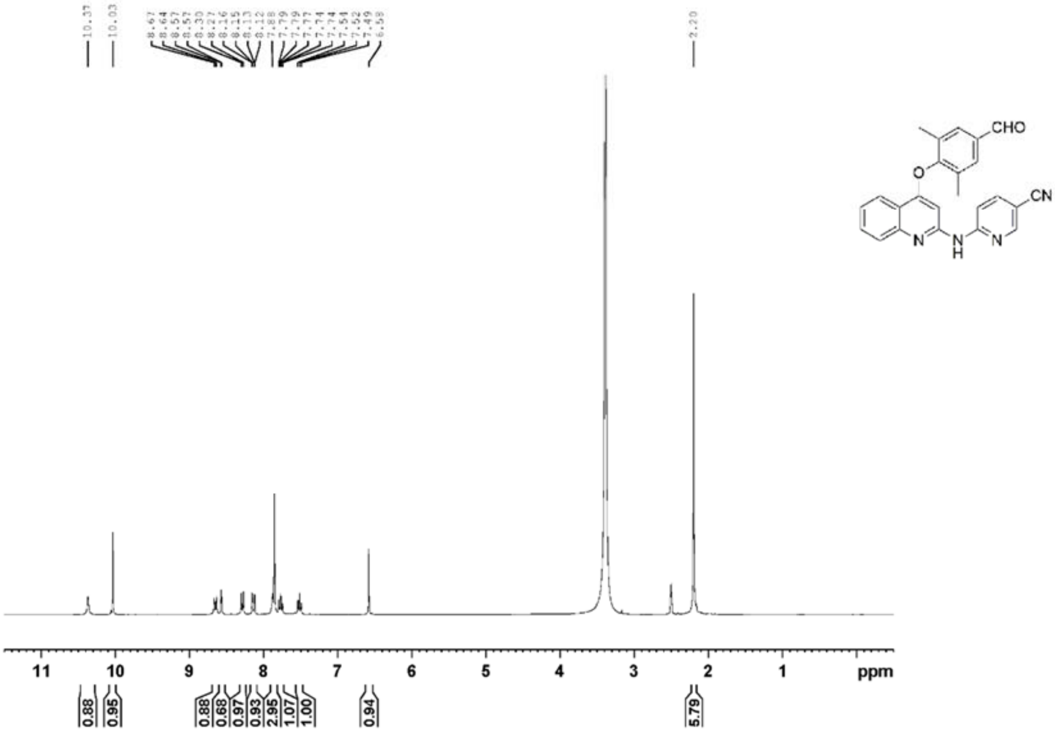

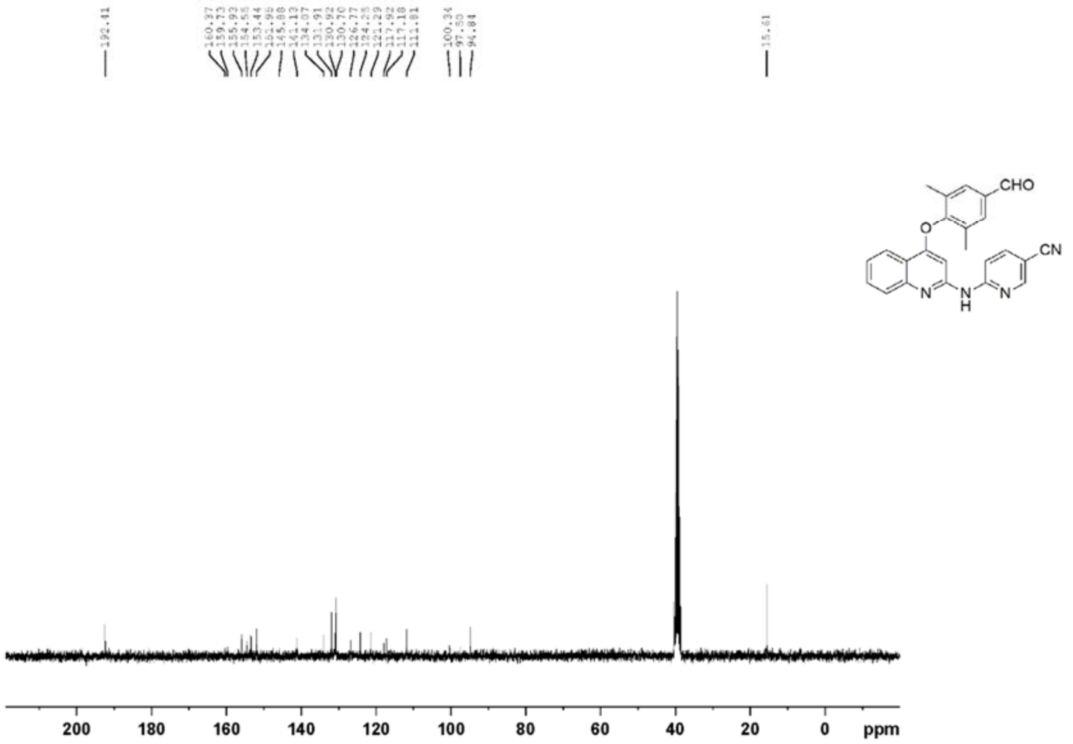

192.41
160.37
159.73
155.93
154.55
153.44
151.95
145.88
141.13
134.07
131.91
130.92
130.70
126.77
124.25
121.29
117.92
117.18
111.81
100.34
97.50
94.84
15.41
CHO
O
CN
N
N
H
N
200
180
160
140
120
100
80
60
40
20
0
ppm

4-(4′-cyanophenoxy)- 2-(5''-cyanopyridin-2''ylamino)quinoline (6c)

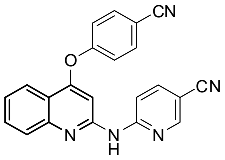

Analysis:

Mp. 225.7-225.9 °C

^{1}H-NMR (300 MHz, $CDCl_3$): 6.86 (s, 1H, ArH-3), 7.30 (d, 2H, J = 8.8 Hz, ArH-2′, 6′), 7.46 (td, 1H, J = 7.6 Hz, ArH-3″), 7.70-7.81 (m, 3H, ArH-6, 3′, 5′), 7.83-7.94 (m, 2H, ArH-7, 8), 8.11 (dd, 1H, J = 8.4, 0.7, ArH-5), 8.30 (bs, 2H, ArH-4″, NH) and 8.46 (d, 1H, J = 1.7 Hz, ArH-6″)

^{13}C-NMR (75 MHz, $CDCl_3$): 98.8, 102.0, 112.3, 117.4, 118.1, 120.9, 121.7, 124.9, 131.5, 134.6, 140.8, 140.9 and 151.7

HRMS (+ESI): $C_{22}H_{14}N_5O$ $[M+H]^+$ requires 364.1193, found 364.1193

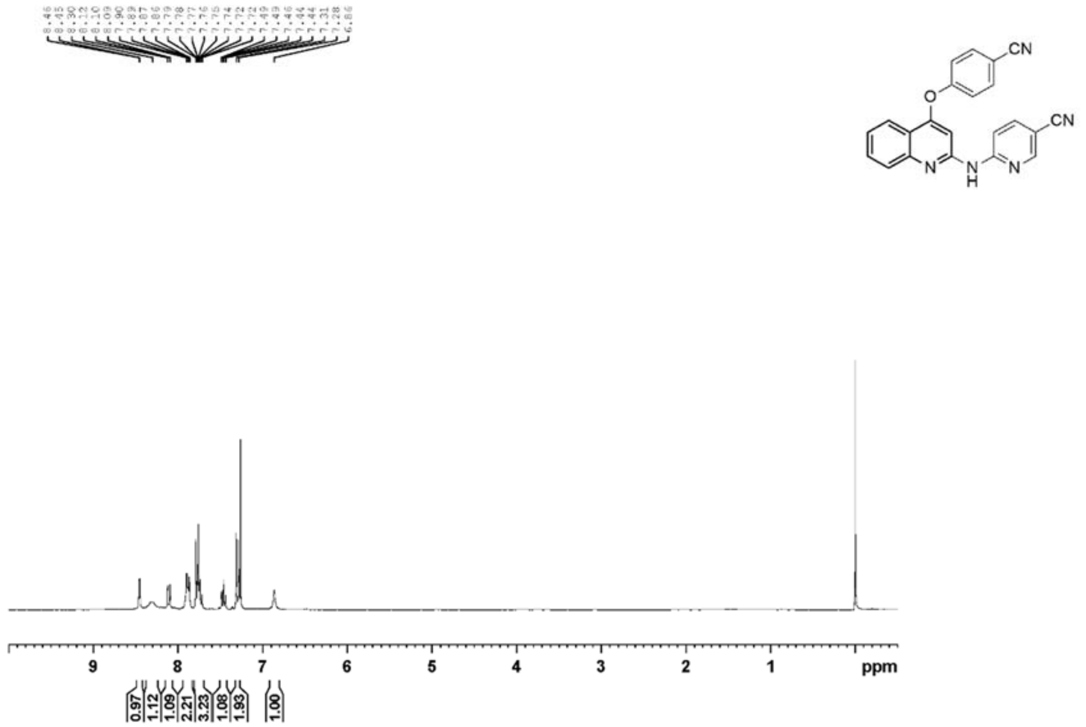

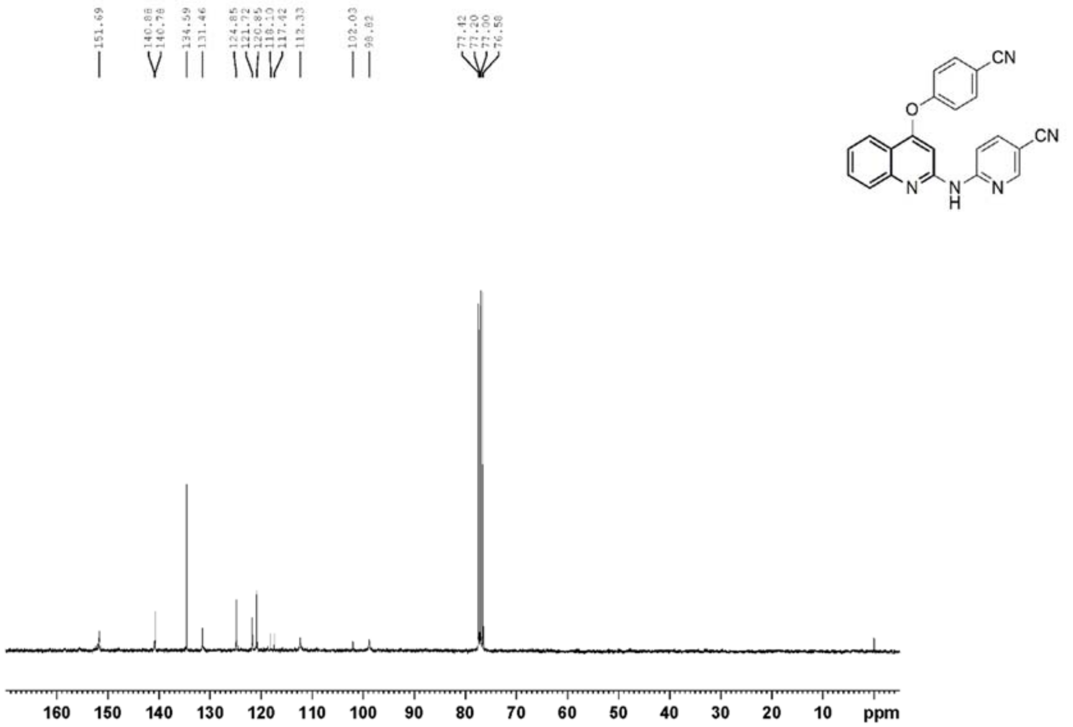

4-(2′,6′-Dimethyl-4′-cyanophenoxy)-2-(5''-cyanopyridin-2''ylamino)quinoline (6d)

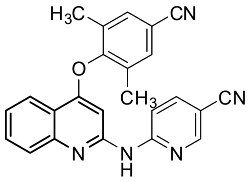

Analysis:

Mp. 229.4-230.3 °C

^{1}H-NMR (300 MHz, $CDCl_3$): 2.21 (s, 6H, $ArCH_3$-2′, 6′), 6.12 (s, 1H, ArH-3), 7.43-7.57 (m, 3H, ArH-3″, 3′, 5′), 7.75 (td, 1H, J = 7.8, 1.4 Hz, ArH-6), 7.82-7.97 (m, 2H, ArH-7, 8), 8.23-8.44 (m, 3H, ArH-5, 4″, NH) and 8.58 (d, 1H, J = 8.3 Hz, ArH-6″)

^{13}C-NMR (75 MHz, $CDCl_3$): 15.9, 93.8, 101.7, 110.0, 112.3, 117.5, 117.7, 118.2, 121.5, 124.5, 127.2, 131.1, 132.9, 133.1, 140.8, 148.3, 151.6, 152.5, 153.6, 155.7 and 160.6

HRMS (+ESI): $C_{24}H_{18}N_5O$ $[M+H]^+$ requires 392.1506, found 392.1494

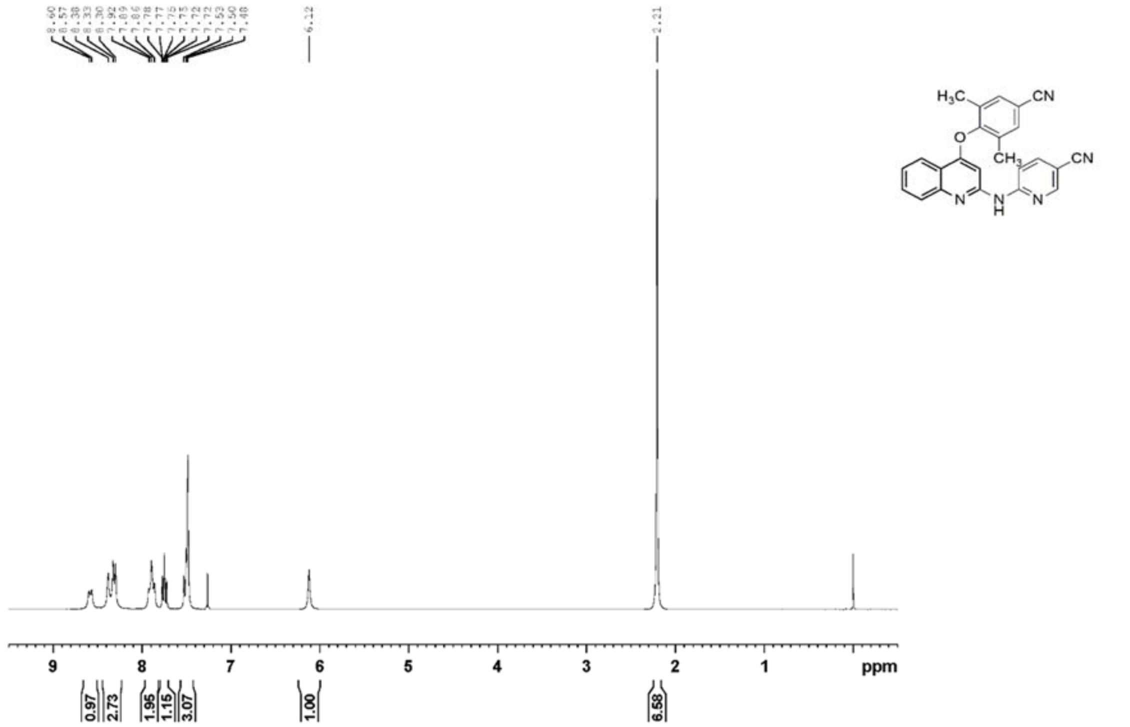

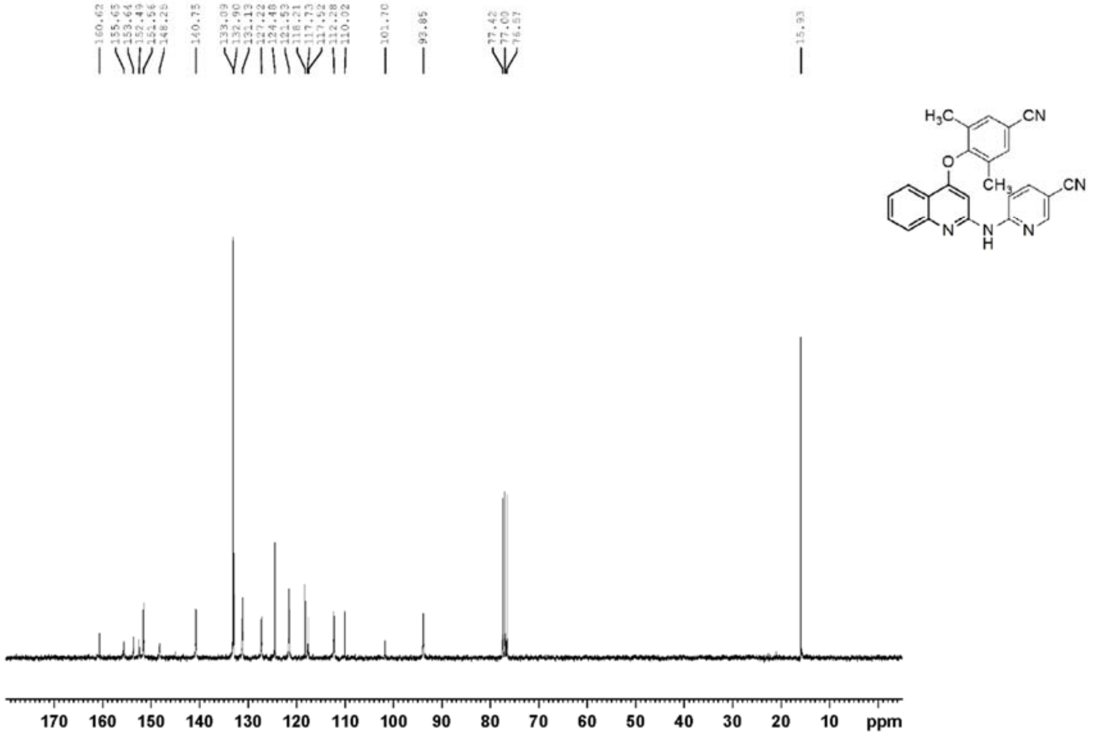
160.62
155.65
153.64
152.49
151.56
148.25
140.75
133.09
132.90
131.13
127.22
124.48
121.53
118.21
117.73
117.52
112.28
110.02
101.70
93.85
77.42
77.00
76.57
15.93
H_3C
CN
O
CH_3
CN
N
N
H
N
170
160
150
140
130
120
110
100
90
80
70
60
50
40
30
20
10
ppm

4,4′-di-(4′-formylphenoxy)-2,2′-biquinoline (7a)

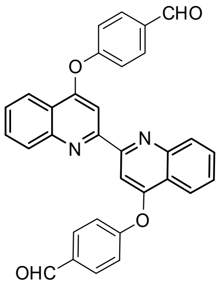

Analysis:

Mp. 288.7-289.3 °C

^{1}H-NMR (300 MHz, $CDCl_3$): 7.33-7.42 (m, 4H, 2×ArH-2′, 6′), 7.59 (td, 2H, J = 7.7, 1.1 Hz, 2×ArH-3), 7.76 (td, 2H, J = 7.7, 1.4 Hz, 2×ArH-6), 7.98-8.06 (m, 4H, 2×ArH-3′, 7′), 8.11 (d, 2H, J = 8.3 Hz, 2×ArH-8), 8.21-8.30 (m, 4H, 2×ArH-5, 5′) and 10.0 (s, 2H, 2×CHO)

^{13}C-NMR (75 MHz, $CDCl_3$): 105.1, 120.1, 121.7, 122.1, 127.2, 129.8, 130.4, 130.5, 132.2, 133.1, 149.6, 157.0, 160.4, 160.7 and 190.8

HRMS (+ESI): $C_{32}H_{21}N_2O_4$ $[M+H]^+$ requires 497.1496, found 497.1491

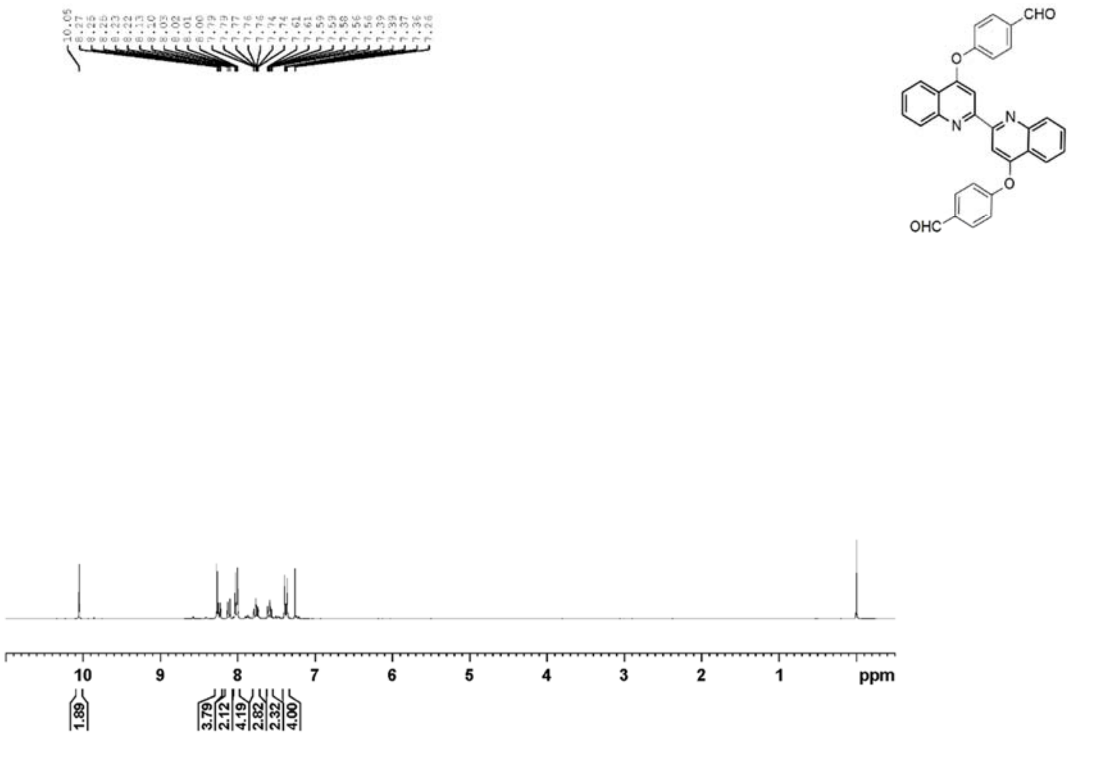

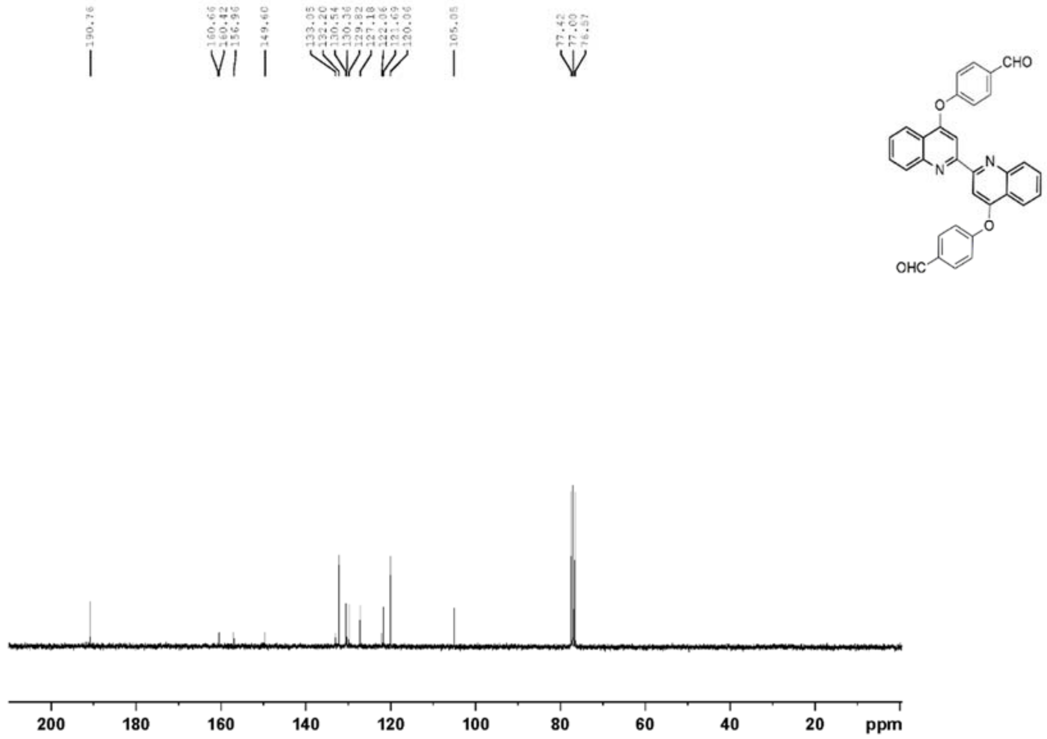
190.76
160.66
160.42
156.96
149.60
133.05
132.20
130.54
130.36
129.82
127.18
122.06
121.69
120.06
105.05
77.42
77.00
76.57
CHO
O
N
N
O
OHC
200
180
160
140
120
100
80
60
40
20
ppm

4,4′-di-(2',6'-Dimethyl-4'-formylphenoxy)-2,2′-biquinoline (7b)

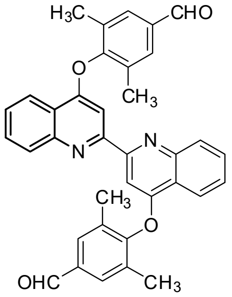

Analysis:

Mp. 300 °C (Decomposed)

^{1}H-NMR (300 MHz, $CDCl_3$ + CD_3OD): 2.27 (s, 12H, 2×$ArCH_3$-2′, 6′), 7.55 (s, 2H, 2×ArH-3), 7.59-7.69 (m, 2H, 2×ArH-6), 7.71-7.86 (m, 6H, 2×ArH-7, 3′, 5′), 8.04 (d, 2H, J = 8.3 Hz, 2×ArH-8), 8.45 (d, 2H, J = 8.1 Hz, 2×ArH-5) and 10.0 (s, 2H, 2×CHO)

^{13}C-NMR (75 MHz, $CDCl_3$ + CD_3OD): 16.1, 100.0, 120.5, 121.4, 126.7, 129.3, 130.3, 130.9, 132.4, 133.9, 149.0, 155.3, 157.1 and 160.1

HRMS (+ESI): $C_{36}H_{29}N_2O_4$ $[M+H]^+$ requires 553.2122, found 553.2116

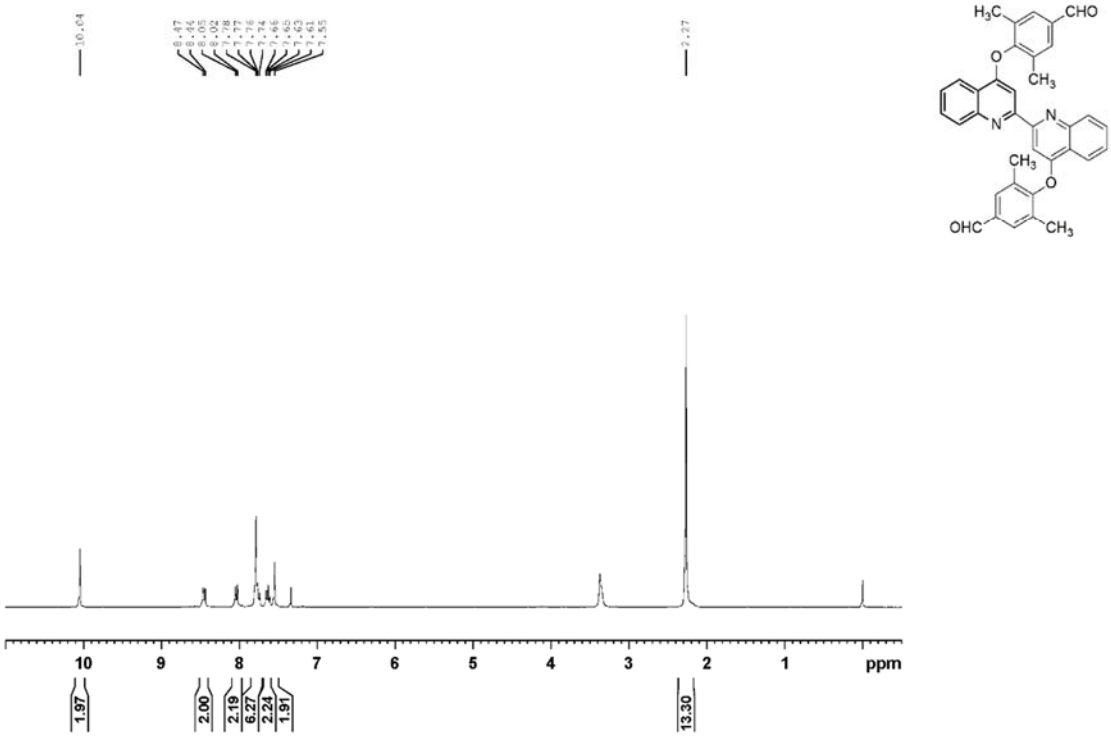

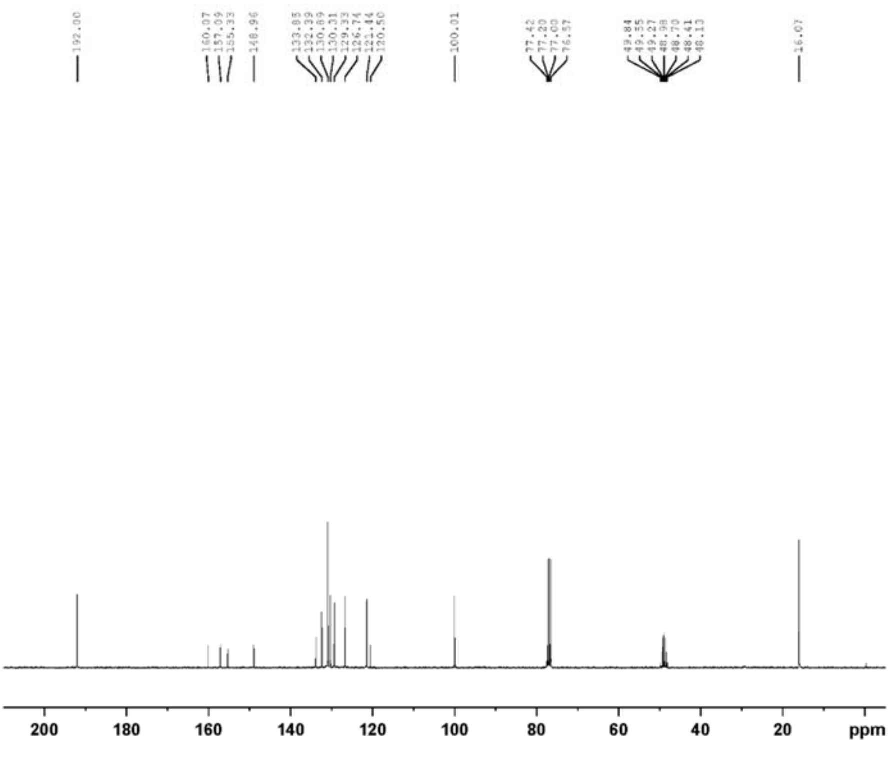
192.00
160.07
157.09
155.33
148.96
133.85
132.39
130.89
130.31
129.33
126.74
121.44
120.50
100.01
77.42
77.20
77.00
76.57
49.84
49.55
49.27
48.98
48.70
48.41
48.13
16.07
200
180
160
140
120
100
80
60
40
20
ppm

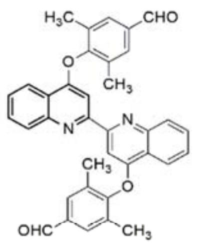
H3C
CHO
O
CH3
N
N
CH3
O
OHC
CH3

2-(4′-formylphenoxy)-4-(4″-cyanophenyl)-aminoquinoline (8a)

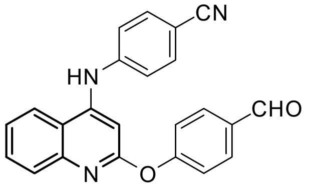

Analysis:

Mp. 238.5 °C – 238.9 °C

^{1}H-NMR (400 MHz, $CDCl_3$ + CD_3OD): 6.95 (s, 1H, ArH-3), 7.36-7.40 (m, 2H, ArH-2″, 6″), 7.41-7.45 (m, 3H, ArH-2′, 6′, NH), 7.50 (td, 1H, J = 6.0, 2.0 Hz, ArH-6), 7.64-7.71 (m, 3H, ArH-7, 3″, 5″), 7.79 (d, 1H, J = 8.0, 2.0 Hz, ArH-8), 7.95 (dt, 2H, J = 8.0, 2.0 Hz, ArH-3′, 5′), 8.13 (d, 1H, J = 8.0 Hz, ArH-5), and 9.97 (s, 1H, CHO)

^{13}C-NMR (75 MHz, $CDCl_3$ + CD_3OD): 95.0, 104.6, 113.8, 118.8, 119.2, 119.8, 120.2, 121.1, 124.6, 127.5, 130.3, 131.4, 132.1, 133.4, 145.3, 146.9, 149.5, 159.6, 161.3 and 191.4

HRMS (+ESI): $C_{23}H_{16}N_3O_2$ $[M+H]^+$ requires 366.1237, found 366.1244

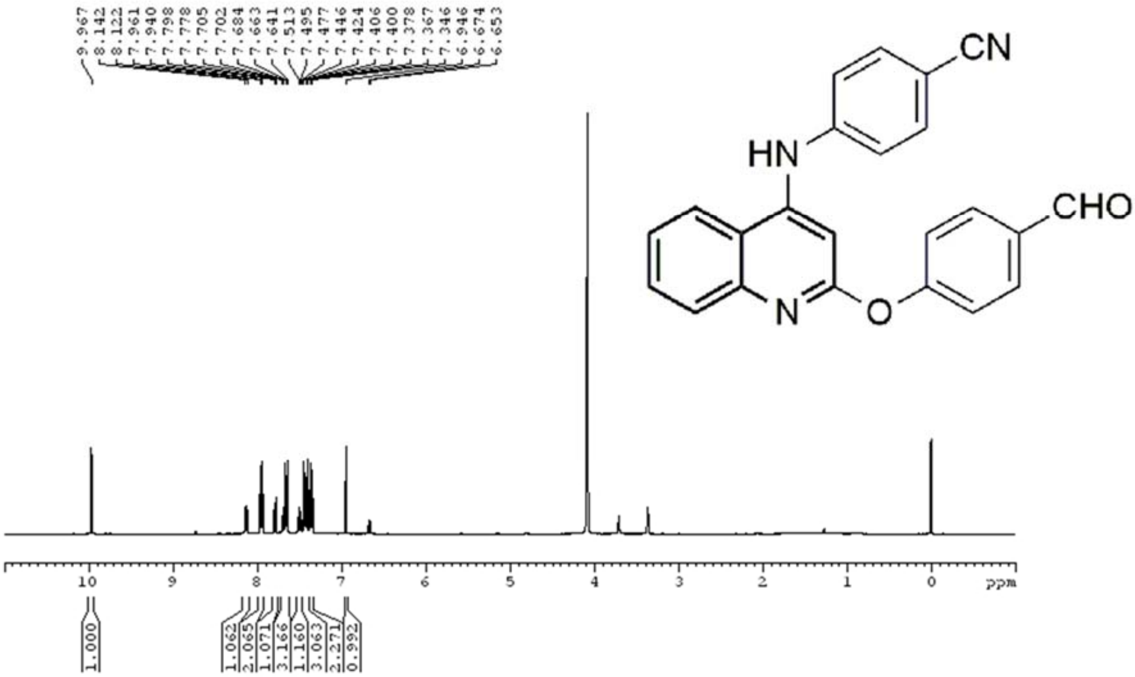

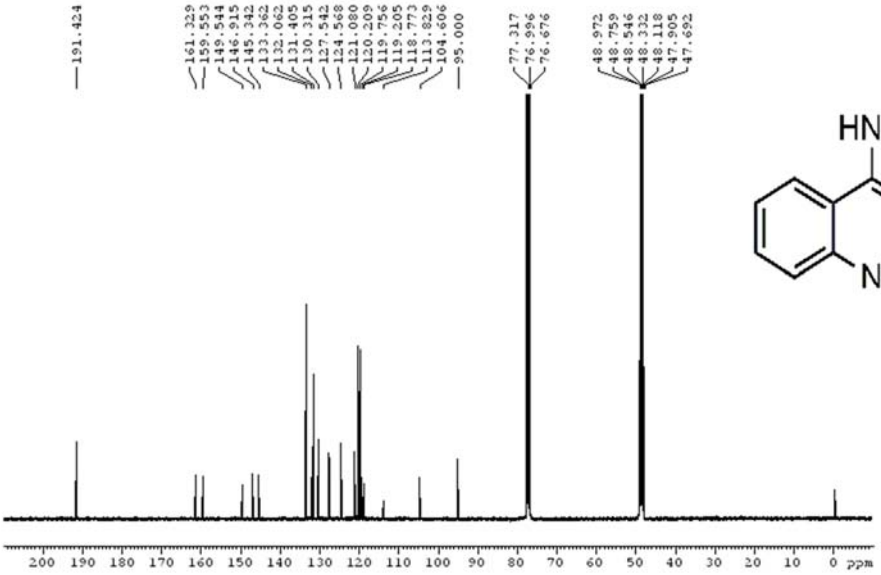
191.424
161.329
159.553
149.544
146.915
145.342
133.362
132.062
131.405
130.315
127.542
124.568
121.080
120.209
119.756
119.205
118.773
113.829
104.606
95.000
77.317
76.996
76.676
48.972
48.759
48.546
48.332
48.118
47.905
47.692
200 190 180 170 160 150 140 130 120 110 100 90 80 70 60 50 40 30 20 10 0 ppm
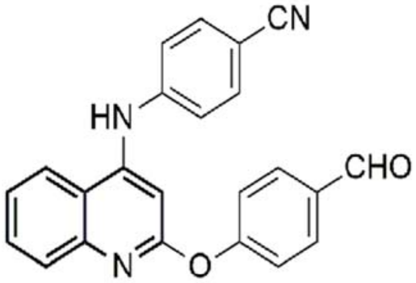
CN
HN
CHO
N
O

2-(2′,6′-Dimethyl-4′-formylphenoxy)-4-(4′′-cyanophenyl)-aminoquinoline (**8b**).

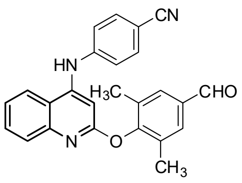

Analysis:

Mp. 200.0-201.3 °C

^{1}H-NMR (300 MHz, $CDCl_3$): 2.21 (s, 6H, $ArCH_3$-2′, 6′), 6.86 (s, 1H, NH), 6.98 (s, 1H, ArH-3), 7.35 (s, 1H, ArH-3′), 7.36 (s, 1H, ArH-5′) 7.42 (td, 1H, J = 7.6, 1.5 Hz, ArH-6′), 7.60 (td, 1H, J = 7.5, 1.2 Hz, ArH-7), 7.63-7.73 (m, 5H, ArH-8, 2′′, 3′′, 5′′, 6′′), 7.85 (d, 1H, J = 7.8 Hz, ArH-5), 9.97 (s, 1H, CHO)

^{13}C-NMR (75 MHz, $CDCl_3$): 16.8, 94.3, 105.9, 118.6, 118.9, 119.7, 119.8, 124.4, 128.9, 130.3, 132.6, 133.5, 133.9, 144.8, 147.7, 147.8, 155.7, 161.3 and 191.8

HRMS (+ESI): $C_{25}H_{20}N_3O_2$ $[M+H]^+$ requires 394.1550, found 394.1542

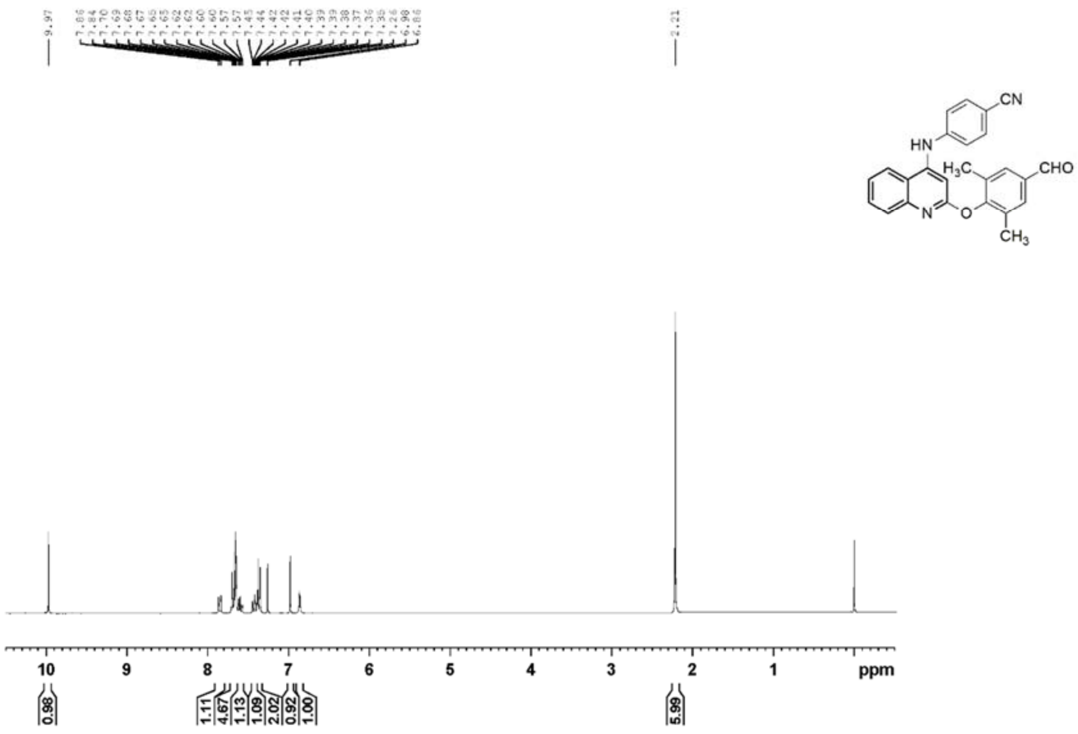

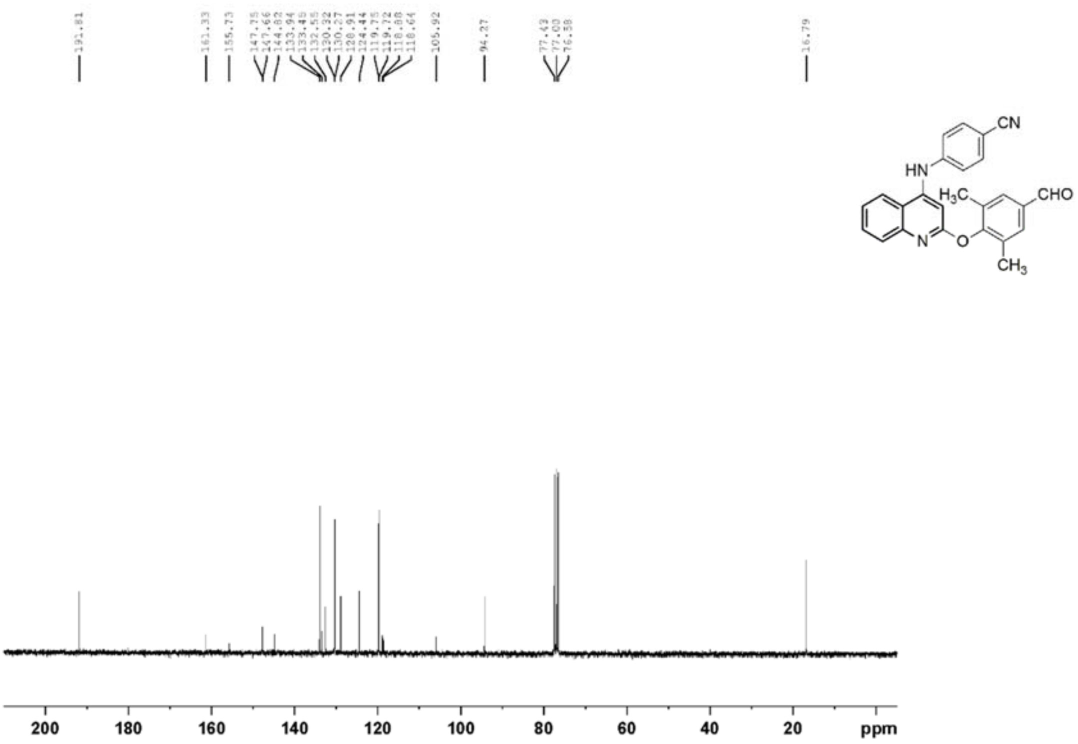

191.81
161.33
155.73
147.75
147.66
144.82
133.94
133.45
132.55
130.32
130.27
128.91
124.44
119.75
119.72
118.88
118.64
105.92
94.27
77.43
77.00
76.58
16.79
CN
HN
H3C
CHO
N
O
CH3
200
180
160
140
120
100
80
60
40
20
ppm

2-(4′-cyanophenoxy)-4-(4′′-cyanophenyl)-aminoquinoline (8c).

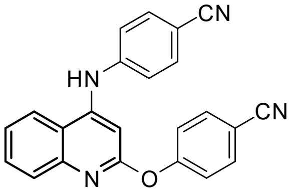

Analysis:

Mp. 187.5 °C – 188.0 °C.

^{1}H-NMR (400 MHz, CD_3OD): 6.87 (s, 1H, ArH-3), 7.37 (d, 2H, J = 8.8 Hz, ArH-2′′, 6′′), 7.45–7.47 (m, 1H, ArH-6), 7.49 (d, 2H, J = 8.8 Hz, ArH-2′, 6′), 7.56–7.58 (m, 1H, ArH-7), 7.61–7.65 (m, 1H, ArH-8), 7.76 (d, 2H, J = 8.8 Hz, ArH-3′′, 5′′), 7.85 (d, 2H, J = 8.8 Hz, ArH-3′, 5′), 8.25 (d, 1H, J = 8.0 Hz, ArH-5) and 9.49 (s, 1H, NH)

^{13}C-NMR (100 MHz, DMSO-d6): 95.1, 104.4, 107.3, 119.2, 119.7, 119.7, 120.5, 122.6, 122.9, 124.9, 128.2, 131.0, 134.2, 134.5, 146.1, 147.2, 149.7, 158.0 and 161.9

HRMS (+ESI) was $C_{23}H_{15}N_4O$ $[M+H]^+$; it requires 363.1240, but has 363.1245.

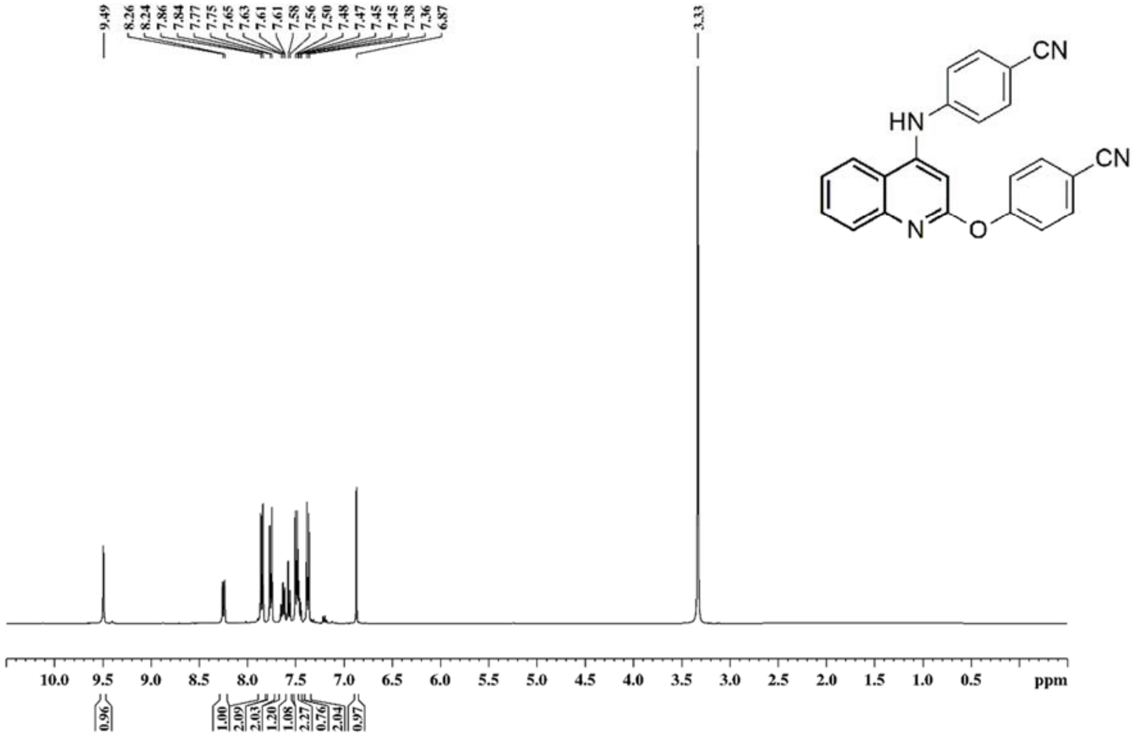

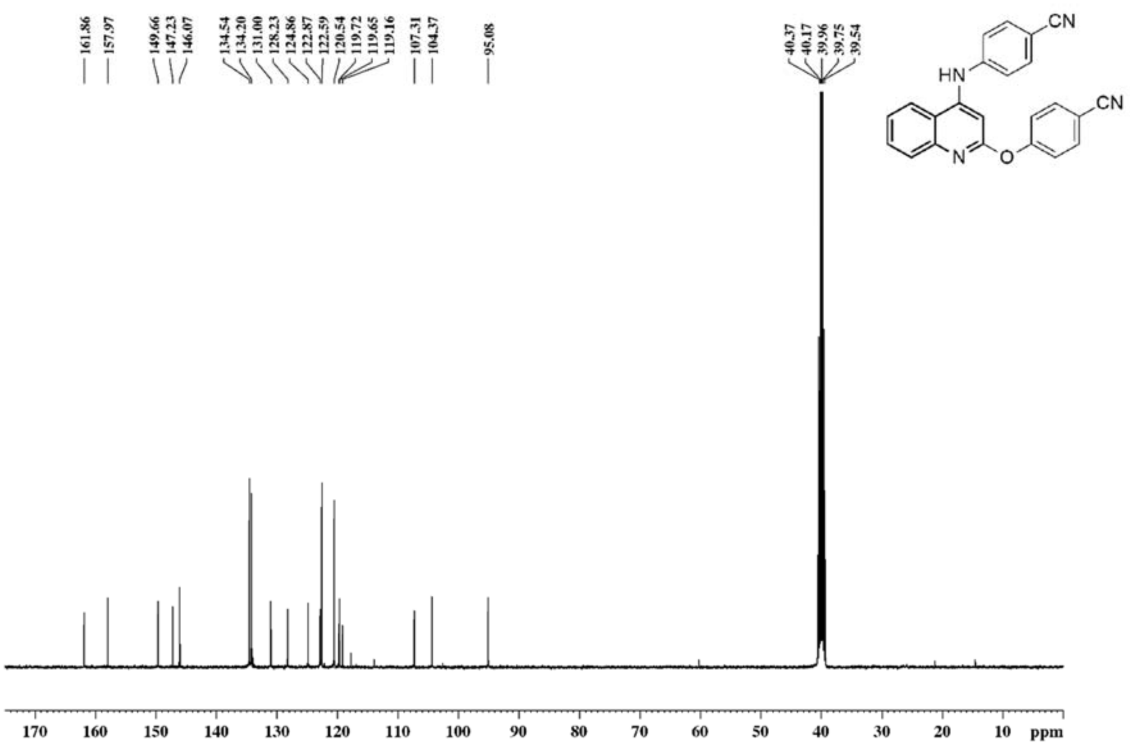
161.86
157.97
149.66
147.23
146.07
134.54
134.20
131.00
128.23
124.86
122.87
122.59
120.54
119.72
119.65
119.16
107.31
104.37
95.08
40.37
40.17
39.96
39.75
39.54
CN
HN
CN
N
O
170 160 150 140 130 120 110 100 90 80 70 60 50 40 30 20 10 ppm

2-(2′,6′-Dimethyl-4′-cyanophenoxy)-4-(4′′-cyanophenyl)-aminoquinoline (8d).

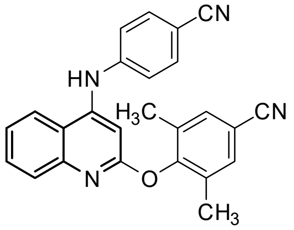

Analysis:

Mp. 201.3 °C – 202.0 °C

^{1}H-NMR (400 MHz, $CDCl_3$): 2.16 (s, 6H, $ArCH_3$-2′, 6′), 6.98 (s, 1H, ArH-3), 7.02 (bs, 1H, NH), 7.38-7.44 (m, 5H, ArH-6, 2′′, 3′′, 5′′, 6′′), 7.58-7.65 (m, 2H, ArH-7, 8), 7.69 (d, 2H, J = 12 Hz, ArH-3′, 5′) and 7.89 (d, 1H, J = 8.0 Hz, ArH-5)

^{13}C-NMR (100 MHz, $CDCl_3$): 16.0, 94.0, 105.8, 108.7, 114.4, 118.7, 118.9, 119.1, 119.8, 120.0, 124.5, 128.8, 130.3, 132.4, 133.1, 133.8, 133.8, 144.8, 147.5, 148.0, 154.3, and 161.1

HRMS (+ESI): $C_{25}H_{19}N_4O$ $[M+H]^+$ requires 391.1553, found 391.1561

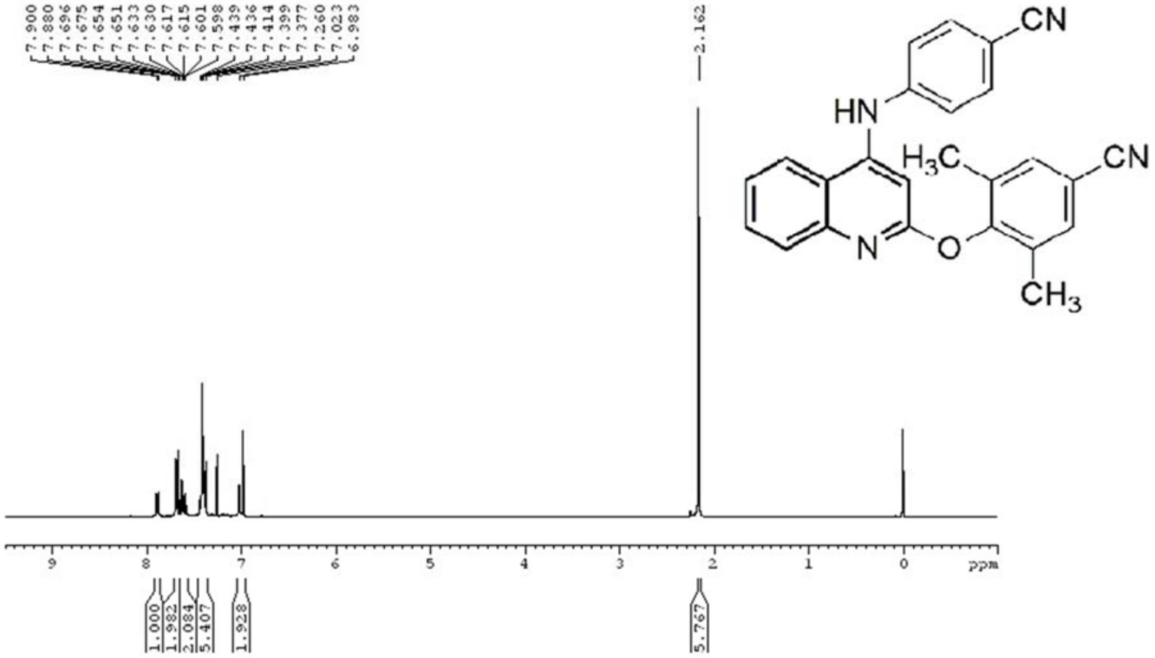

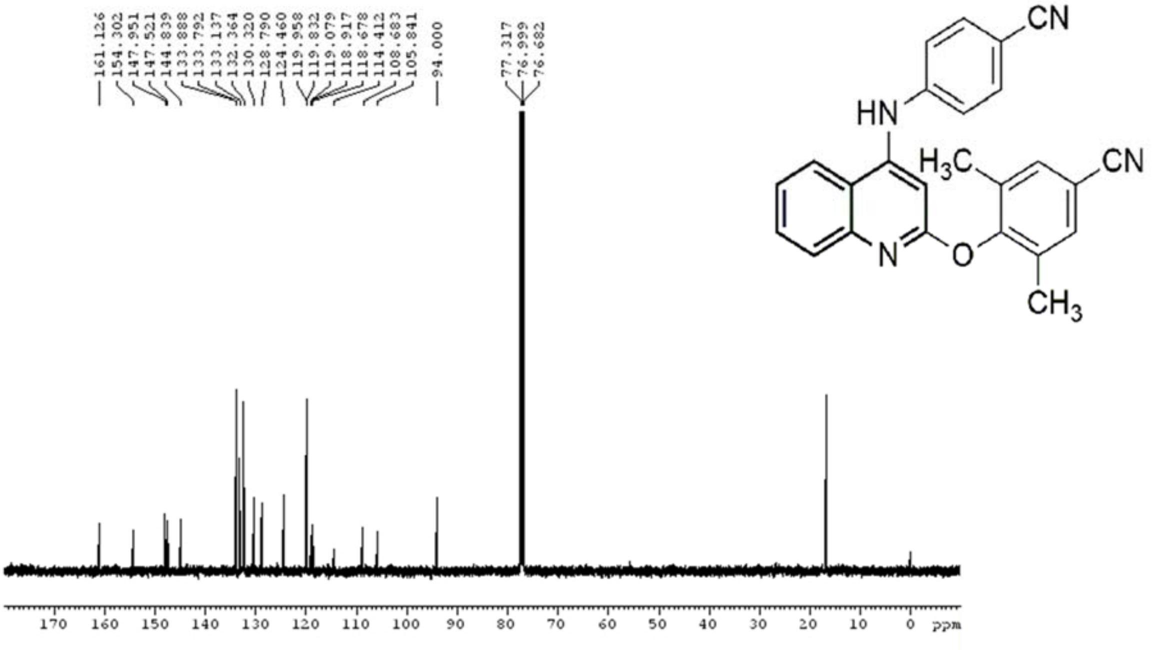
161.126
154.302
147.951
147.521
144.839
133.888
133.792
133.137
132.364
130.320
128.790
124.460
119.958
119.832
119.079
118.917
118.678
114.412
108.683
105.841
94.000
77.317
76.999
76.682
170 160 150 140 130 120 110 100 90 80 70 60 50 40 30 20 10 0 ppm
CN
HN
H_3C
CN
N
O
CH_3

Scheme 3

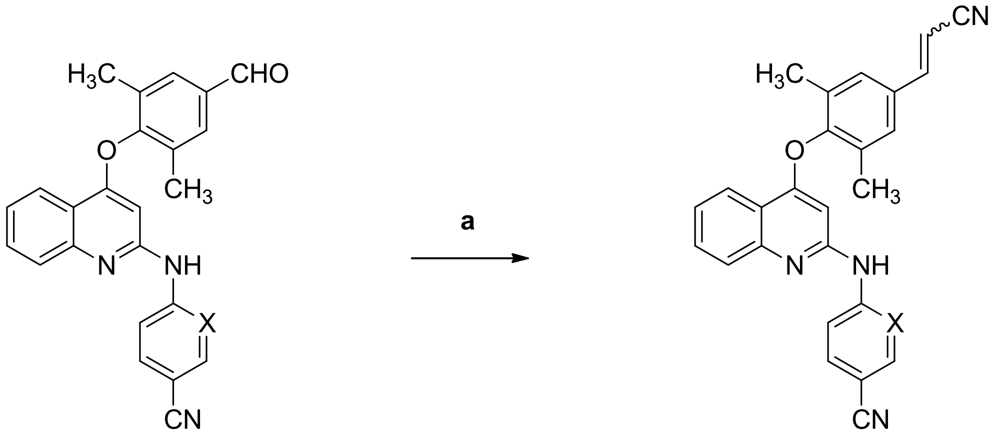

5b (X=C), 6b (X=N) **9b(X=C), 10b (X=N)**

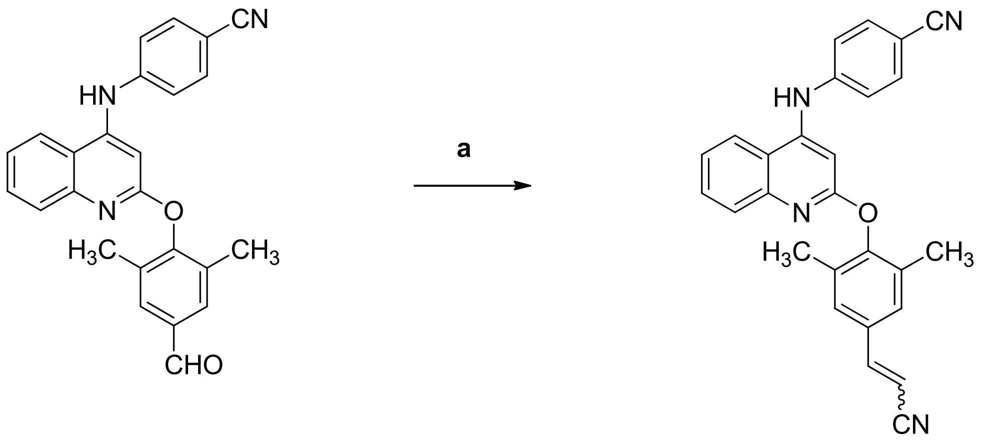

7b **11b**

Synthesis of **9b**, **10b**, and **11b**. Reagents and conditions: a Potassium tert-butoxide (1.5 eq.), diethyl cyanomethyl phosphonate (1.5 eq.), THF, 0–25 °C.

4-(4′-(2′′-(E)-cyanovinyl)-2′,6′-dimethyl-phenoxy)-2-(4′′-cyanophenyl)-aminoquinoline (9b-cis isomer).

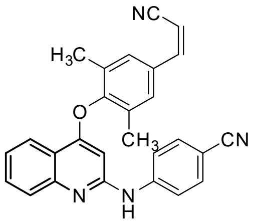

Analysis:

Mp. 217.3-217.9 °C

^{1}H-NMR (300 MHz, $CDCl_3$): 2.20 (s, 6H, $ArCH_3$-2′, 6′), 5.47 (d, 1H, J = 12.1, Vinyl-H), 5.74 (s, 1H, ArH-3), 6.78 (bs, 1H, NH), 7.12 (d, 1H, J = 12.1 Hz, Vinyl-H), 7.45 (td, 1H, J = 7.4, 1.1 Hz, ArH-6), 7.52-7.66 (m, 4H, ArH-3′, 5′, 2′′, 6′′), 7.71 (td, 1H, J = 7.7, 1.4 Hz, ArH-7), 7.81-7.94 (m, 3H, ArH-8, 3′′, 5′′) and 8.29 (dd, 1H, J = 8.3, 1.0 Hz, ArH-5)

^{13}C-NMR (75 MHz, $CDCl_3$): 16.1, 93.0, 95.2, 104.0, 117.3, 117.7, 118.2, 119.5, 121.6, 123.9, 127.2, 128.4, 130.1, 130.9, 131.5, 131.9,133.2, 144.6, 147.9, 148.6, 152.1, 153.5 and 161.1

HRMS (+ESI): $C_{27}H_{21}N_4O$ $[M+H]^+$ requires 417.1710, found 417.1712

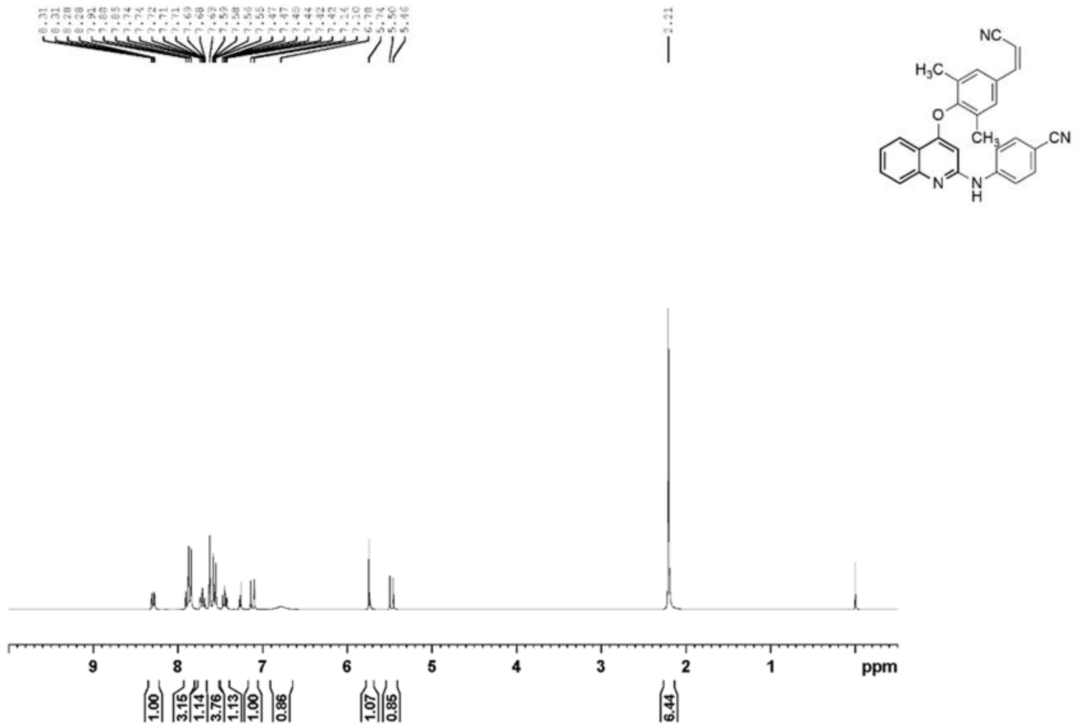

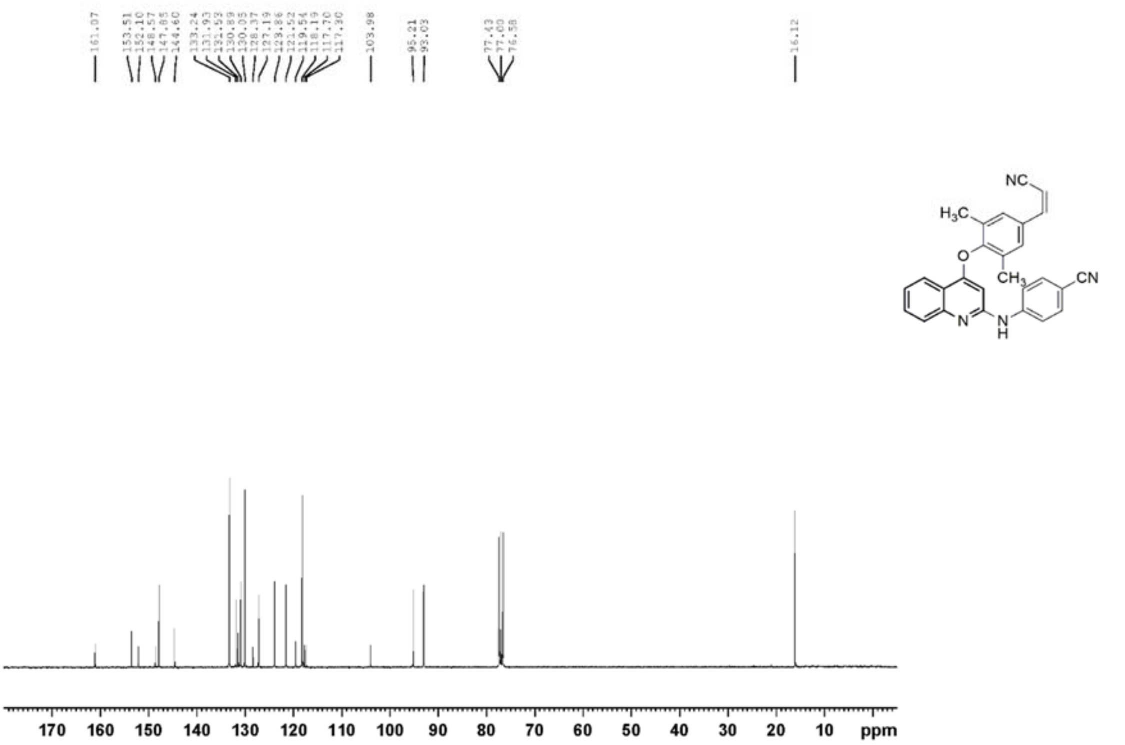
161.07
153.51
152.10
148.57
147.85
144.60
133.24
131.93
131.53
130.89
130.05
128.37
127.19
123.86
121.52
119.54
118.19
117.70
117.30
103.98
95.21
93.03
77.43
77.00
76.58
16.12
NC
H3C
O
CH3
CN
N
NH
170 160 150 140 130 120 110 100 90 80 70 60 50 40 30 20 10 ppm

4-(4′-(2′′-(E)-cyanovinyl)-2′,6′-dimethyl-phenoxy)-2-(4′′-cyanophenyl)-aminoquinoline (9b-trans isomer).

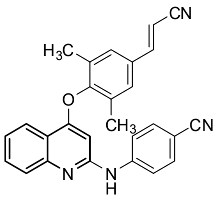

Analysis:

Mp. 214.4-215.1 °C

^{1}H-NMR (300 MHz, $CDCl_3$+CD_3OD): 2.20 (s, 6H, $ArCH_3$-2′, 6′), 5.82 (s, 1H, ArH-3), 5.92 (d, 1H, J = 16.6 Hz, Vinyl-H), 7.30 (s, 2H, ArH-3′, 5′), 7.41 (d, 1H, J = 16.6 Hz, Vinyl-H), 7.40-7.48 (m, 1H, ArH-6), 7.56 (d, 2H, J = 8.7 Hz, ArH-2′′, 6′′), 7.66-7.76 (m, 1H, ArH-7), 7.84-7.94 (m, 3H, ArH-8, 3′′, 5′′) and 8.28 (dd, 1H, J = 8.1, 1.0 Hz, ArH-5)

^{13}C-NMR (75 MHz, $CDCl_3$+CD_3OD): 15.8, 93.2, 95.9, 102.9, 117.48, 117.9, 119.6, 121.3, 123.5, 126.8, 128.2, 130.6, 131.2, 132.1, 133.0, 145.1, 148.4, 149.8, 152.5, 154.0 and 160.7

HRMS (+ESI): $C_{27}H_{21}N_4O$ $[M+H]^+$ requires 417.1710, found 417.1699

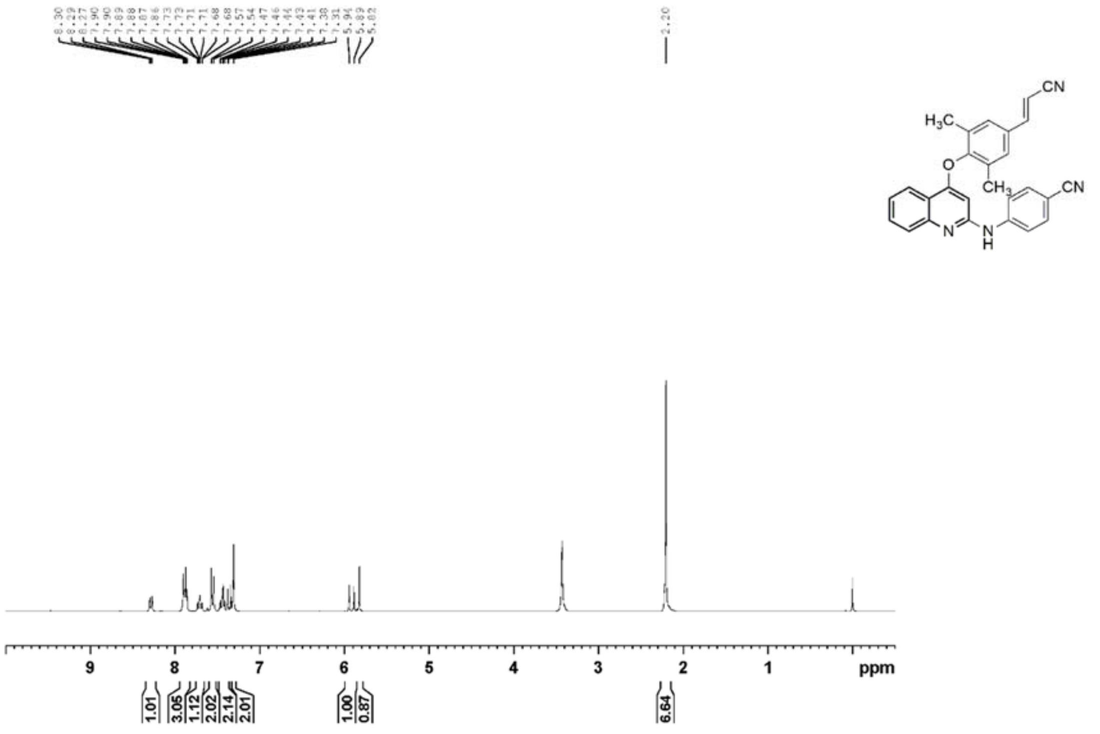

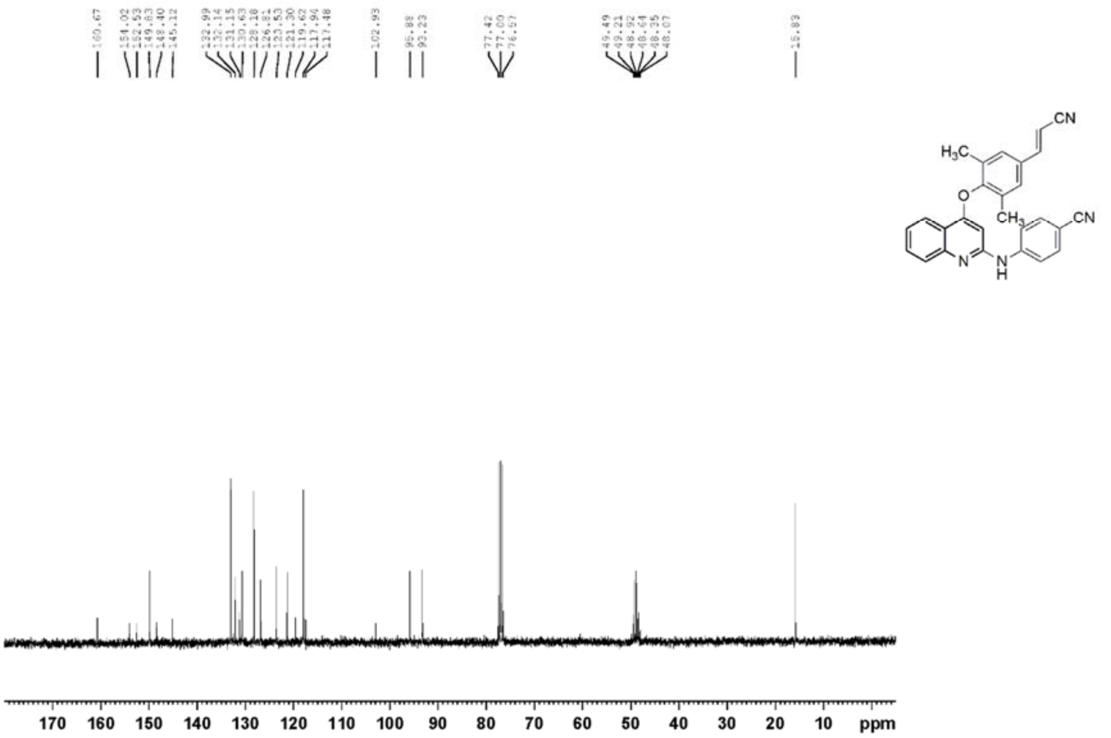
160.67
154.02
152.53
149.63
148.40
145.12
132.99
132.14
131.15
130.63
128.18
126.81
123.53
121.30
119.62
117.94
117.48
102.93
95.88
93.23
77.42
77.00
76.57
49.49
49.21
48.92
48.64
48.35
48.07
15.83
CN
H_3C
O
CH_3
CN
N
NH
170
160
150
140
130
120
110
100
90
80
70
60
50
40
30
20
10
ppm

4-(4′-(2′′-(Z)-cyanovinyl)-2′,6′-dimethyl-phenoxy)-2-(5′′-cyanopyridin-2′′ylamino)-aminoquinoline (10b-cis isomer).

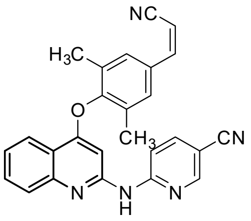

Analysis:

Mp. 239.6-239.9 °C

^{1}H-NMR (300 MHz, $CDCl_3$): 2.20 (s, 6H, $ArCH_3$-2′, 6′), 5.49 (d, 1H, J = 12.1 Hz, Vinyl-H), 6.11 (s, 1H, ArH-3), 7.13 (d, 1H, J = 12.1 Hz, Vinyl-H), 7.49 (t, 1H, J = 7.7 Hz, ArH-3′′), 7.64 (s, 2H, ArH-3′, 5′), 7.74 (td, 1H, J = 7.6, 1.2 Hz, ArH-6), 7.81-7.95 (m, 3H, ArH-5, 7, 8), 8.07 (bs, 1H, NH), 8.27-8.43 (m, 3H, ArH-5, 4′′) and 8.58 (d, 1H, J = 8.6 Hz, ArH-6′′)

^{13}C-NMR (75 MHz, $CDCl_3$): 16.1, 93.9, 95.2, 96.5, 112.2, 117.2, 117.6, 118.0, 121.7, 124.3, 127.2, 128.4, 130.1, 131.0, 131.6, 131.8, 140.7, 147.7, 148.3, 149.5, 151.6, 152.0, 152.5, 155.7 and 161.2

HRMS (+ESI): $C_{26}H_{20}N_5O$ $[M+H]^+$ requires 418.1662, found 418.1657

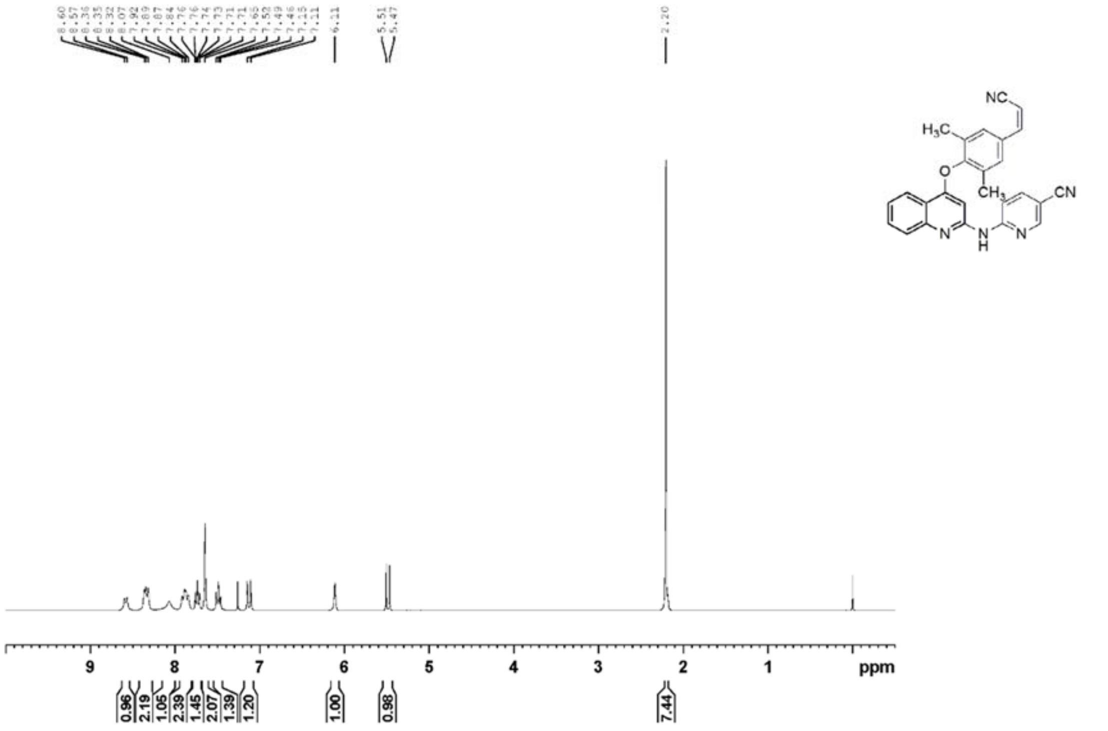

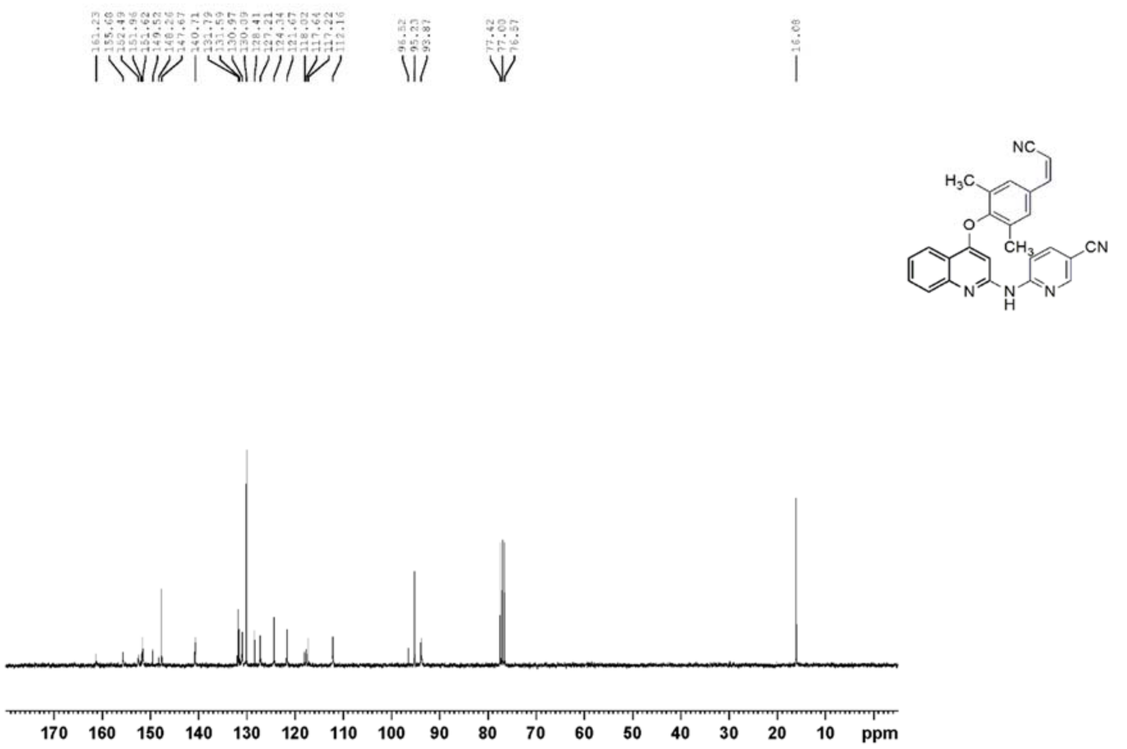

4-(4′-(2′′-(Z)-cyanovinyl)-2′,6′-dimethyl-phenoxy)-2-(5′′-cyanopyridin-2′′ylamino)-aminoquinoline (10b-trans isomer).

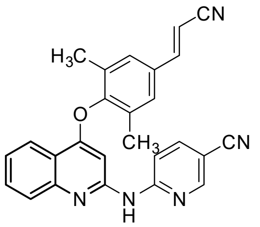

Analysis:

Mp. 236.6-237.4 °C

^{1}H-NMR (300 MHz, $CDCl_3$+CD_3OD): 2.21 (s, 6H, $ArCH_3$-2′, 6′), 5.93 (d, 1H, J = 16.7 Hz, Vinyl-H), 6.28 (s, 1H, ArH-3), 7.32 (s, 2H, ArH-3′, 5′), 7.41 (d, 1H, J = 16.7 Hz, Vinyl-H), 7.46-7.55 (td, 1H, J = 7.6, 1.0 Hz, ArH-3′′), 7.69-7.78 (m, 1H, ArH-6), 7.81-7.93 (m, 2H, ArH-7, 8), 8.28-8.39 (m, 2H, ArH-5, 4′′) and 8.53 (s, 1H, ArH-6′′)

^{13}C-NMR (75 MHz, $CDCl_3$+CD_3OD): 15.8, 96.0, 100.9, 117.5, 117.9, 121.5, 124.2, 128.2, 129.1, 130.8, 131.2, 131.9, 140.5, 149.7, 151.3, 152.3 and 160.9

HRMS (+ESI): $C_{26}H_{20}N_5O$ $[M+H]^+$ requires 418.1662, found 418.1657

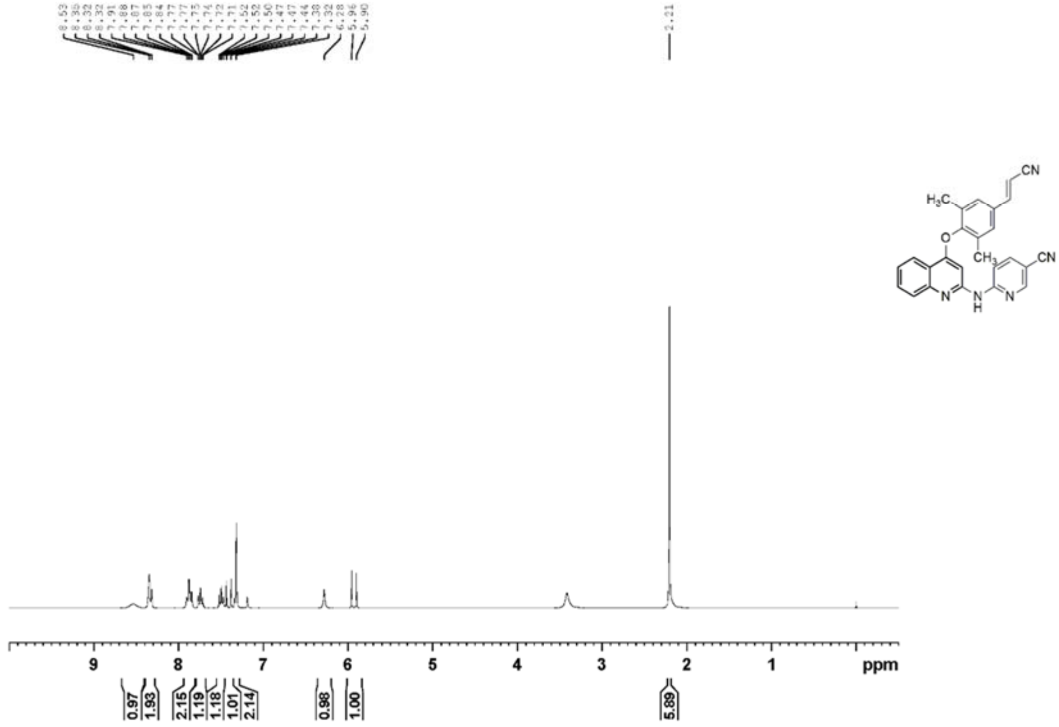

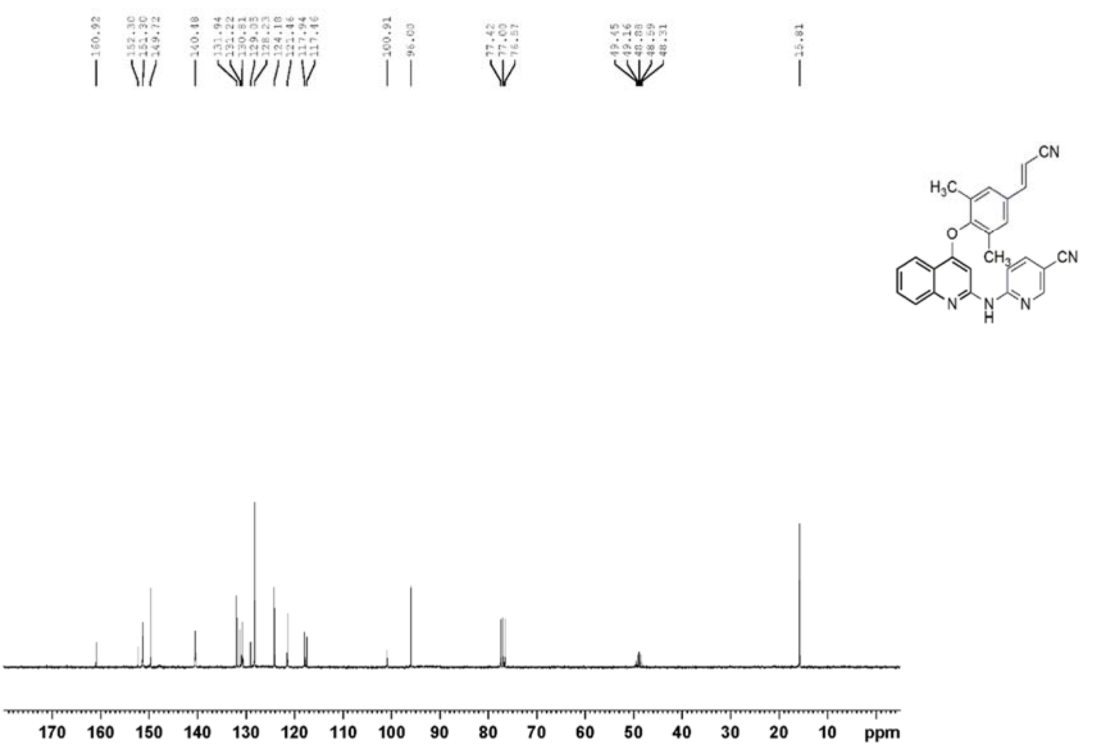
160.92
152.30
151.30
149.72
140.48
131.94
131.22
130.81
129.05
128.23
124.18
121.46
117.94
117.46
100.91
96.00
77.42
77.00
76.57
49.45
49.16
48.88
48.59
48.31
15.81
CN
H_3C
O
CH_3
CN
N
N
H
N
170 160 150 140 130 120 110 100 90 80 70 60 50 40 30 20 10 ppm

4-(4′-(2″-(E, Z)-cyanovinyl)-2′,6′-dimethyl-phenoxy)-2-(4″-cyanophenyl)-aminoquinoline (11b) (trans: cis isomers; 1: 0.33).

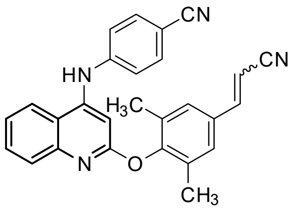

Analysis:

Mp. 180.7-181.0 °C

^{1}H-NMR (300 MHz, $CDCl_3$, E, Z mixture): 2.16 (s, 6H, $ArCH_3$-2′, 6′), 5.80 (d, 1H, J = 16.5, Vinyl-H), 6.89-6.99 (m, 1H, ArH-3), 7.20 (s, 1H, ArH-3′, 5′), 7.29-7.39 (m, 3H, Vinyl-H, ArH-2″, 6″), 7.54-7.63 (m, 2H, ArH-3″, 5″), 7.63-7.70 (m, 3H, ArH-6, 7, 8) and 7.86 (d, 1H, J = 8.3 Hz, ArH-5)

^{13}C-NMR (75 MHz, $CDCl_3$, E, Z mixture): 16.8, 93.8, 94.2, 94.3, 95.1, 105.6, 105.7, 117.6, 118.5, 118.7, 119.0, 119.6, 119.7, 119.9, 124.3, 127.8, 128.8, 129.5, 130.3, 130.4, 130.6, 132.1, 132.3, 133.9, 144.9, 147.7, 148.3, 150.4, 152.6, 153.1, 161.5 and 161.6

HRMS (+ESI): $C_{27}H_{21}N_4O$ $[M+H]^+$ requires 417.1710, found 417.1701

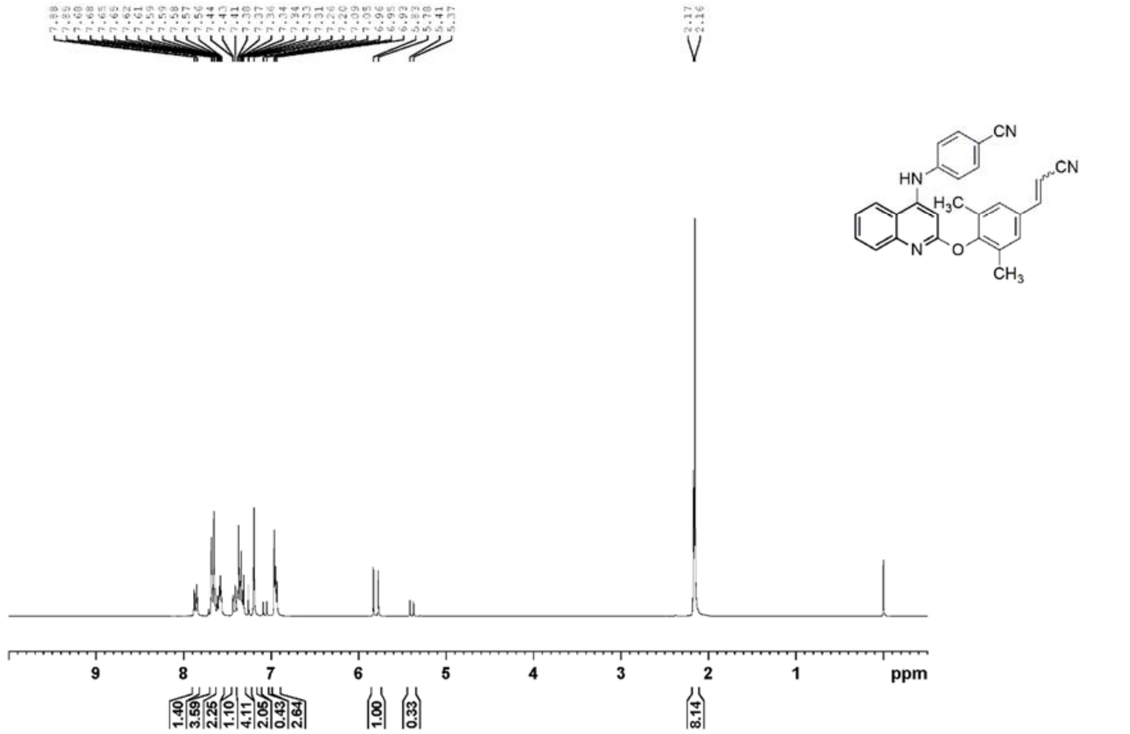

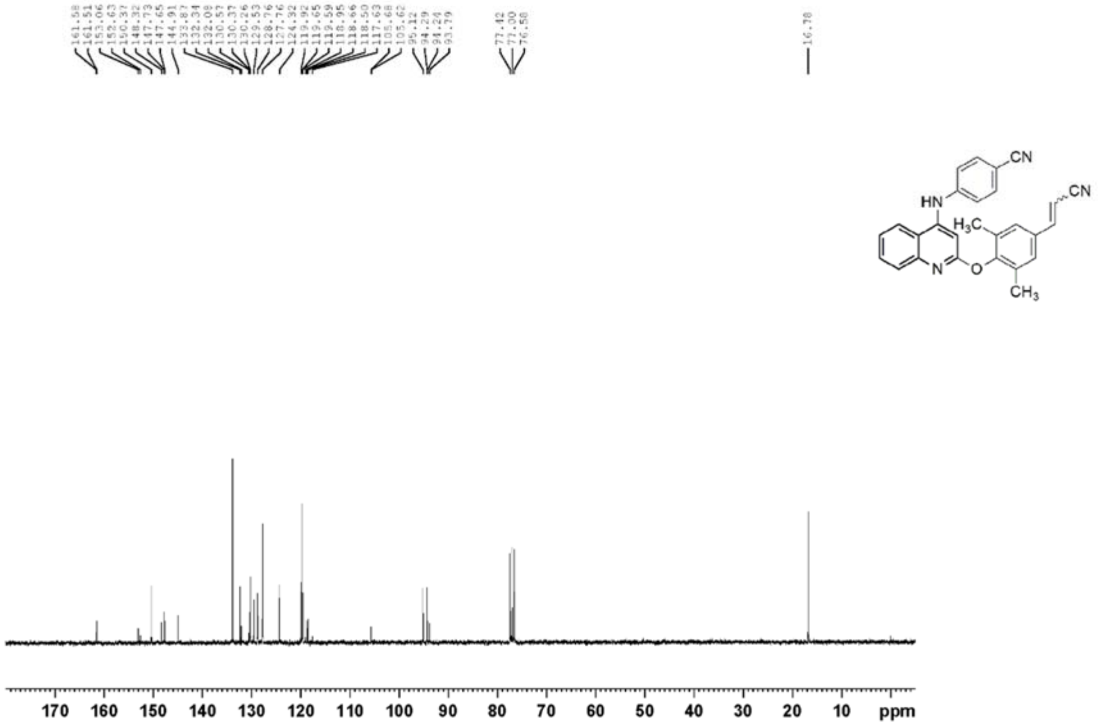

161.58
161.51
153.06
152.63
150.37
148.32
147.73
147.65
144.91
133.87
132.34
132.08
130.57
130.37
130.26
129.53
128.76
127.76
124.32
119.92
119.65
119.59
118.95
118.66
118.50
117.63
105.68
105.62
95.12
94.29
94.24
93.79
77.42
77.00
76.58
16.78
CN
HN
CN
H_3C
N
O
CH_3
170
160
150
140
130
120
110
100
90
80
70
60
50
40
30
20
10
ppm

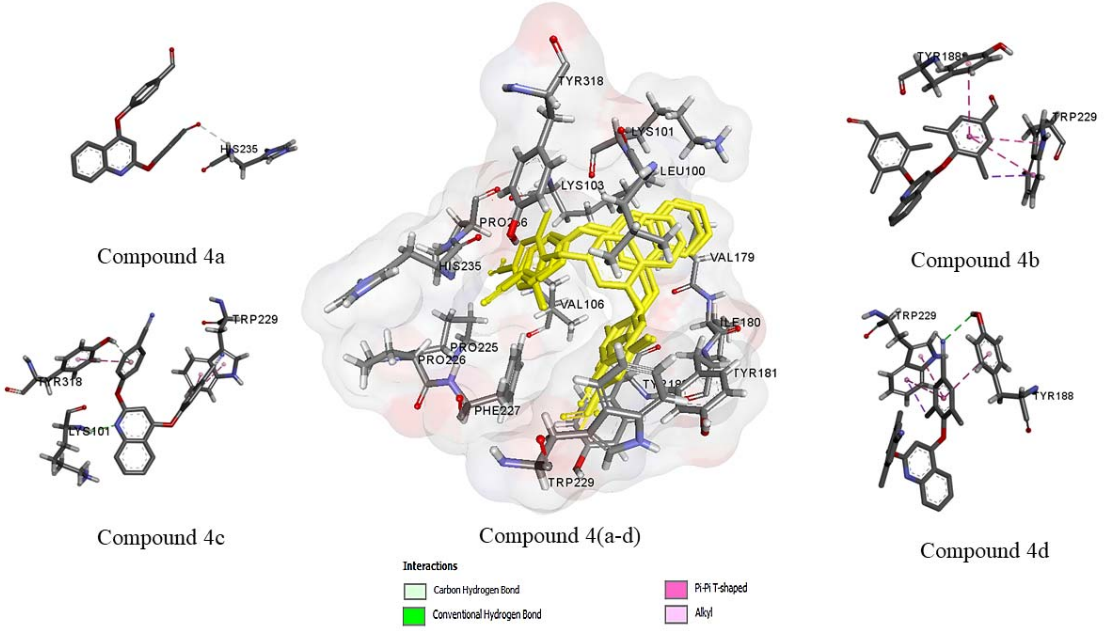

Figure 1S. The binding interaction between the compound 4(a-d) and HIV-1 RT using molecular docking.

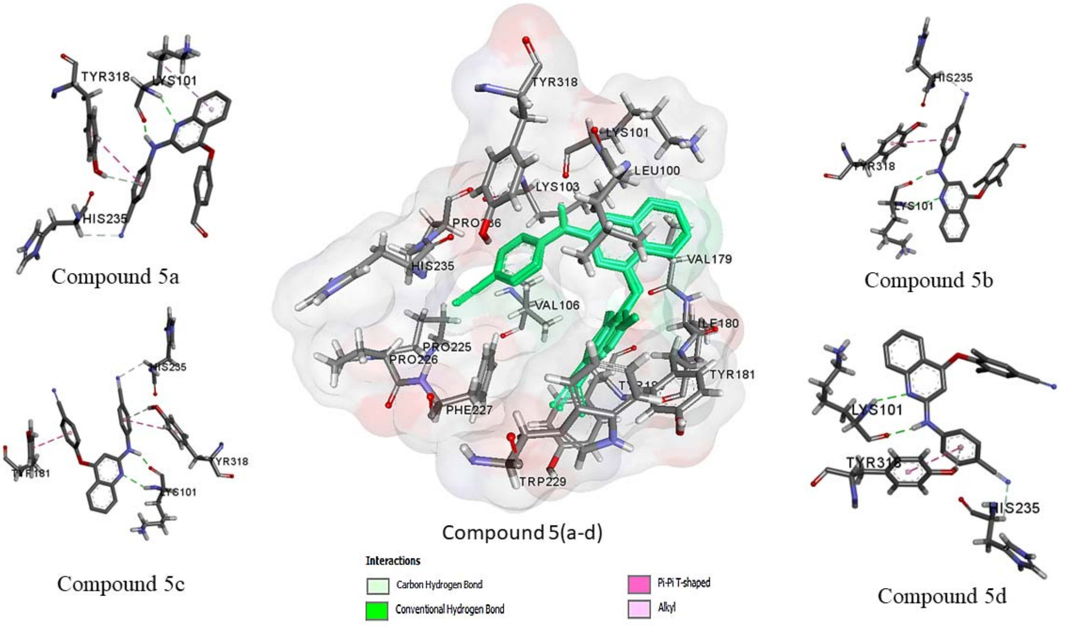

Figure 2S. The binding interaction between the compound 5(a-d) and HIV-1 RT using molecular docking.

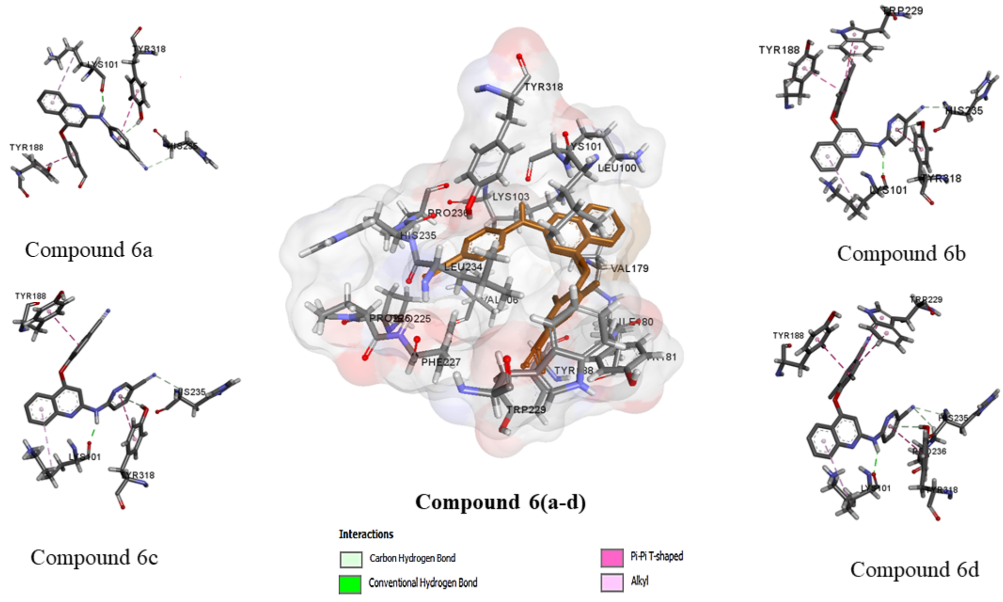

Figure 3S. The binding interaction between the compound 6(a-d) and HIV-1 RT using molecular docking.

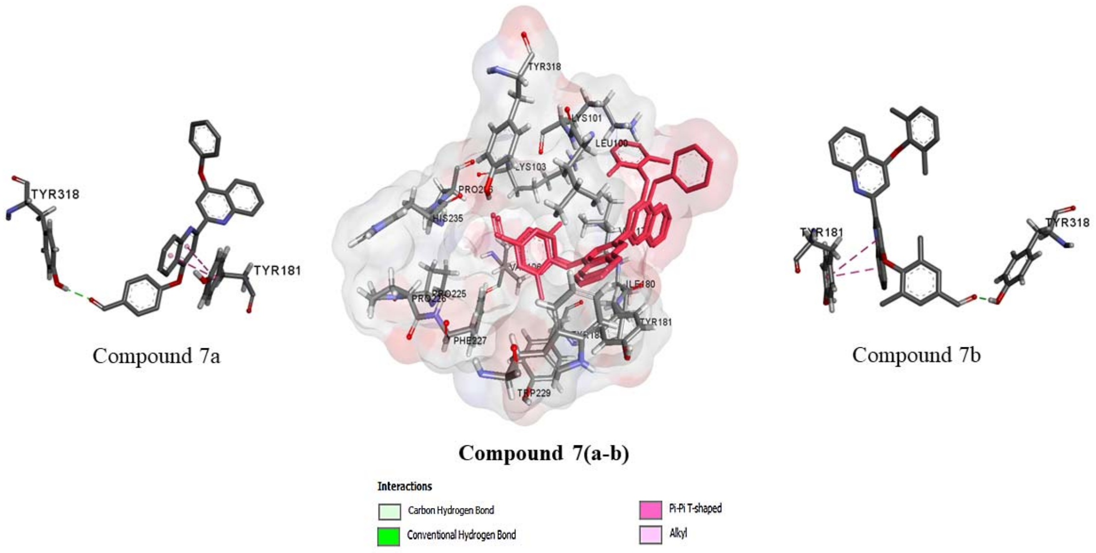

Figure 4S. The binding interaction between the compound 7(a-b) and HIV-1 RT using molecular docking.

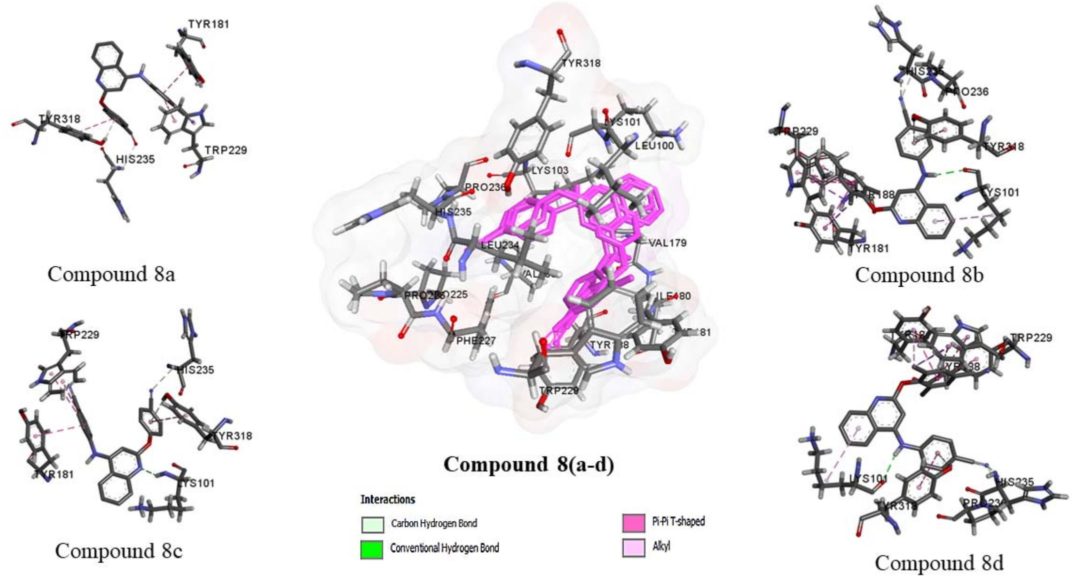

Figure 5S. The binding interaction between the compound 8(a-d) and HIV-1 RT using molecular docking.

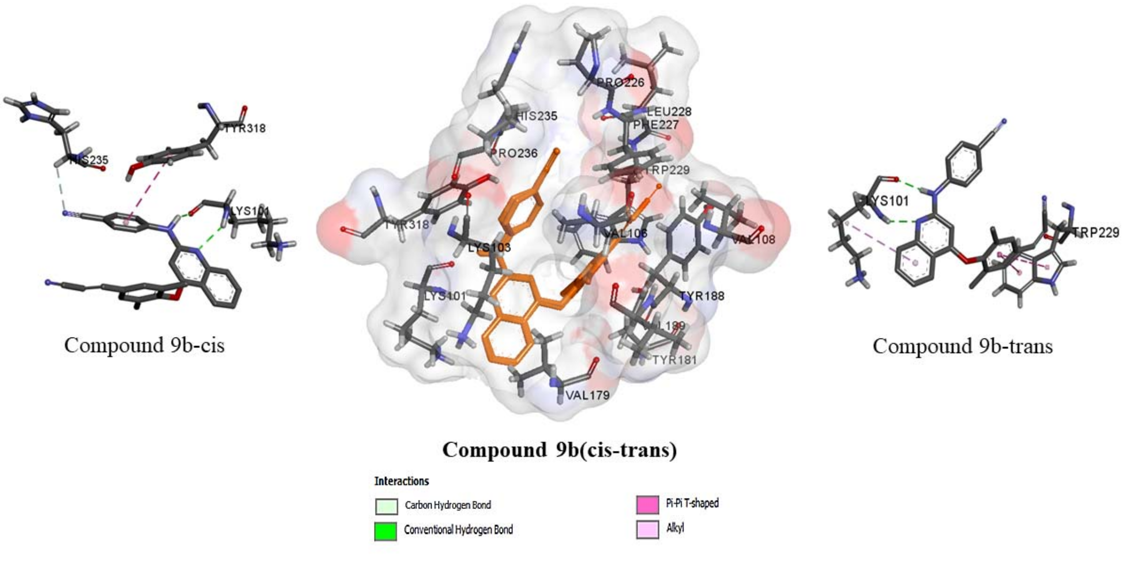

Figure 6S. The binding interaction between the compound 9b(cis-trans) and HIV-1 RT using molecular docking.

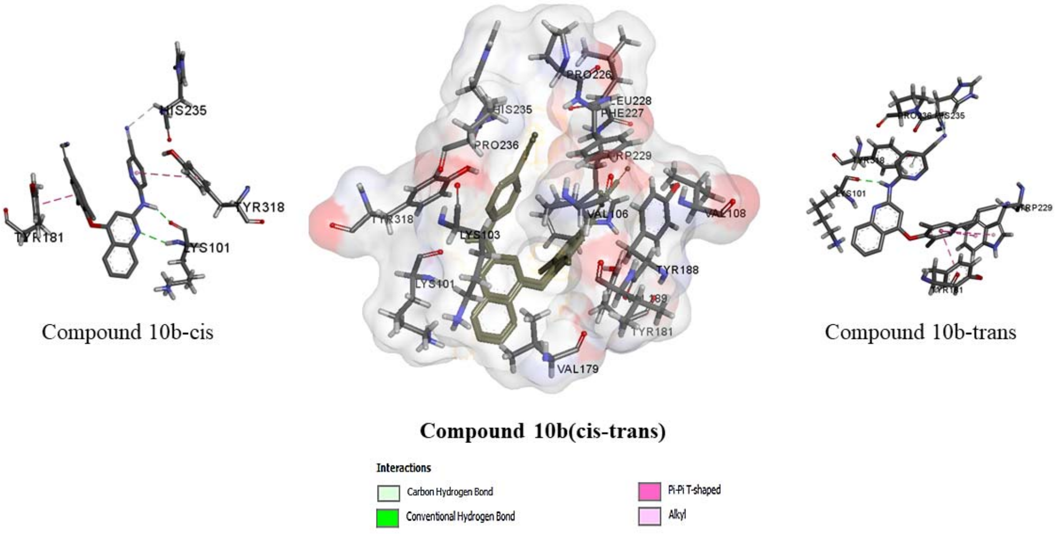

Figure 7S. The binding interaction between the compound 10b(cis-trans) and HIV-1 RT using molecular docking.

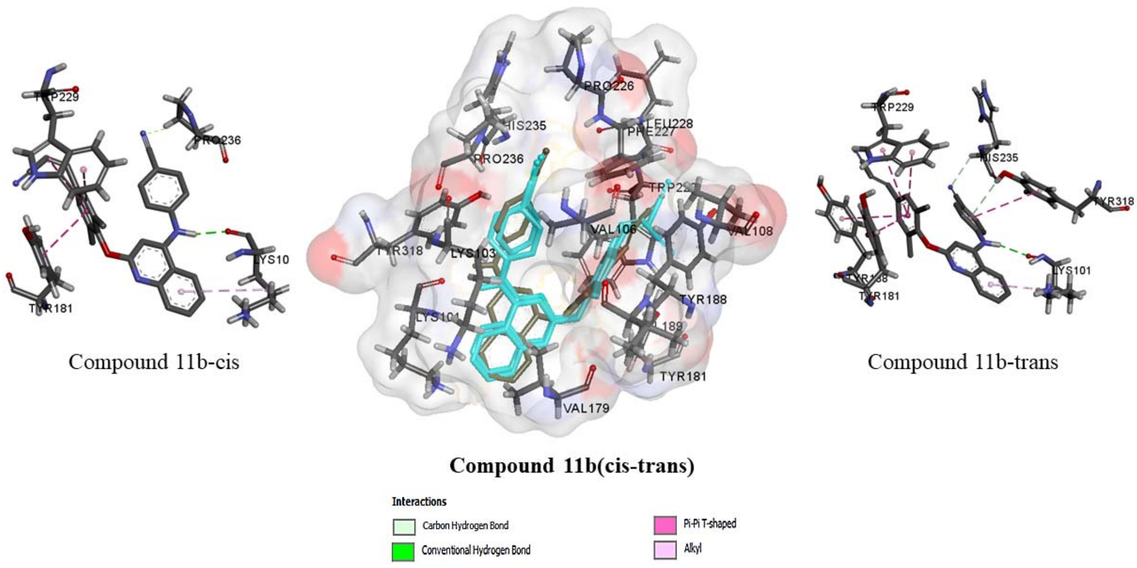

Figure 8S. The binding interaction between the compound 11b(cis-trans) and HIV-1 RT using molecular docking.

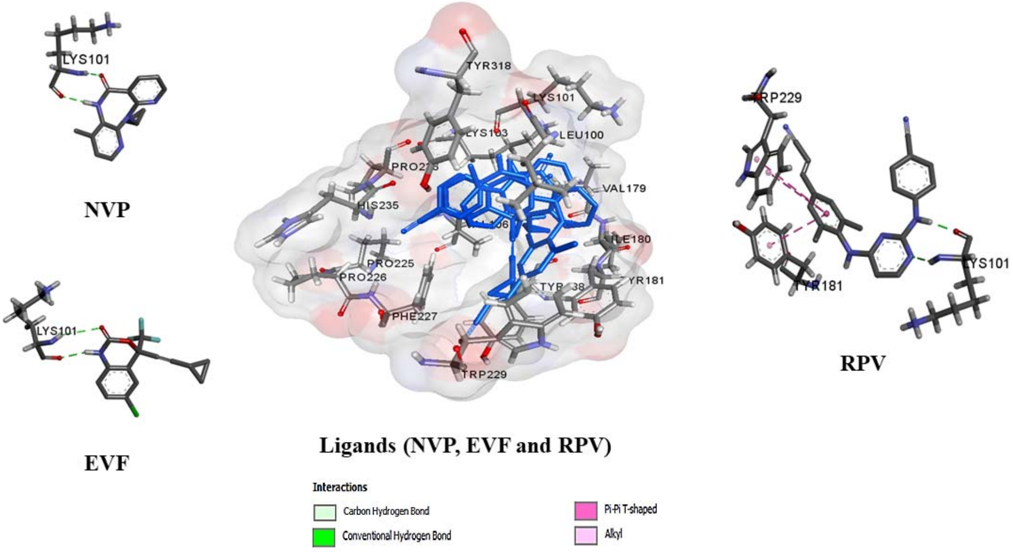

Figure 9S. The binding interaction between ligands (NVP, EVF and RPV) and HIV-1 RT using molecular docking.
